# Building a 4E interview-grounded theory model: A case study of demand factors for customized furniture

**DOI:** 10.1371/journal.pone.0282956

**Published:** 2023-04-27

**Authors:** Chengmin Zhou, Wenhui Gu, Xin Luo, Jake Kaner

**Affiliations:** 1 Jiangsu Co-Innovation Center of Efficient Processing and Utilization of Forest Resources, Nanjing, China; 2 College of Furnishings and Industrial Design, Nanjing Forestry University, Nanjing, Jiangsu, China; 3 School of Art and Design, Nottingham Trent University, Nottingham, United Kingdom; Sichuan Agricultural University, CHINA

## Abstract

With the lifestyle change, users’ demand for furniture has shown a trend for personalization and diversification. The customized furniture market is growing rapidly and gradually becoming an indispensable choice for lifestyle items. The present qualitative study sought to identify the influencing factors and relationships of user demand for customized furniture. This study constructed a 4E semi-structured interview guide, which means that interviews were conducted from 4 dimensions: essential information, information extraction, user experience, and product expectation. The interview results were coded and analyzed in combination with grounded theory. Based on the identified 38 concepts and 10 categories, we obtain 4 main categories: fundamental condition, operation behaviour, sensory value and emotional value. For the factors that affect the demand of customized furniture users, customized furniture enterprises can start from 2 levels of publicity and product design to meet user demand and improve the user purchase probability.

## Introduction

Furniture is closely related to human life and is an indispensable thing in daily life and social work [1]. People’s pursuit of the functionality and aesthetics of furniture in the living space has prompted continuous innovation in the home improvement industry. Consequently, the concept of customized furniture has manifested in consumer lifestyle choices. As an important part of furniture, customized furniture mainly contains customized kitchen cabinets, customized wardrobes and some other cabinets. With the improvement of production technology of furniture enterprises and the reduction of per capita living area, customized furniture can better meet the needs of contemporary users, make full use of indoor area and improve storage space [2]. According to the Research Report on market prospects and investment opportunities of China’s furniture industry in 2021 released by China Academy of commerce industry, the scale of customized furniture market in 2020 was about 381.1 billion RMB, and the growth rate of customized furniture market in recent seven years has always been maintained at more than 20%. Customized furniture is gradually becoming an indispensable aspect of furniture consumption in people’s daily life in China [3]. Customized furniture attaches importance to users’ needs, caters to their habits and lifestyles, making it the primary choice for young users [4]. In the face of the growing demand for customized furniture and the variety of styles to choose from, furniture enterprises need to explore how to transmit information to their target groups and meet the operational and emotional needs of users [5].

Current research on customized furniture mainly includes 4 directions: production and manufacturing, product design, plate selection and user demand. Alvin Toffler first proposed the concept of mass customization in the book Future Shock, which entails offering goods and services tailored to individual customers’ demand at the price and speed of mass production [6]. Industry 4.0 introduces new chances for businesses to attain mass customization by using technology in traditional industrial sectors like the furniture sector [7]. Under the umbrella of industry 4.0, significant advancements in the fields of intelligent manufacturing, 3D printing, and artificial intelligence have been made. David analyzed the future development direction of North American customized furniture from the perspectives of agility, lean manufacturing, and clustering, and proposed that the supply chains need to be configured to respond to changing consumer demand quickly [8]. Yan also proposed a rapid customization design method of a new furniture panel production line based on digital twin [9]. 3D printing technology is widely used in various fields. It serves as a method of personalized design in the furniture industry. However, its application mainly focuses on producing unique product series to meet customers’ personal preferences and realize personalized customization [10]. To ensure the convenience of customized furniture production, designers can use design syntax and parametric design methods to design and optimize furniture models [11, 12]. Industrial software, such as CAD, CAE and CAM, is also used to design and produce customized furniture for fast and accurate digital design and modification of furniture products [13]. Customized furniture needs to consider individual size variability and fully apply ergonomic methods to design. Wu tried to use 3D scanning system and motion capture to obtain user dimensions and apply ergonomic assessment methods to customize furniture design in a virtual environment [14]. In addition, some scholars are conducting research on customized furniture materials. The decorated panels are the main materials of customized furniture [15]. However, some decorative panels furniture (mainly chipboard furniture) emits a high level of formaldehyde, endangering human health and having several adverse effects [16].

Leung proposed that good furniture should meet the physical and psychological needs of users [17]. Customized furniture can fully use space to meet users’ functional needs [18]. Users can choose the style and materials of customized furniture to match the interior decoration style and meet their aesthetic needs of users [19]. Current studies have focused on the exploration of users’ demand for customized furniture. No one has explored the factors that influence the demand of customized furniture users and lacked the exploration of attribution of user demand. This paper aims to explore the influencing factors and internal laws of customized furniture demand using semi-structured interviews and grounded theory. Consequently, the current paper seeks to fill the gap in the literature and guide customized furniture enterprises to focus on the underlying factors and mechanisms that influence user demand.

The structure of the paper is organized as follows: Section 2 is a literature review on semi-structured interviews and customized furniture. Section 3 is three coding processes and theoretical saturation tests. Section 4 analyzes the findings that factors influence user demand. The findings are discussed in section 5. Finally, the conclusion is a summary of the full paper and the limitations.

## Methodology

### Related literature

To learn about user demand, user research frequently conducts interviews and questionnaires [20]. It is challenging to uncover every user demand with a questionnaire [21]. Interviews refer to the researchers obtaining the behavioural characteristics and needs of users in the form of question and answer [22]. Interviews are the most widely used and popular data collection method [23]. According to the interview interaction, interviews can be divided into face-to-face, telephone and video interviews [24]. According to the number of interviewees, they can be divided into individual interviews, focus groups, etc [25]. According to the degree of standardization of interviews, interviews can be divided into structured, semi-structured and unstructured interviews [26]. Structured interviews limit the content of questions and the format of answers. The information obtained is simple to process and analyze, but it is difficult to obtain information beyond the questions [27]. Unstructured interviews only set topics and are free to develop around them without the constraints of guides and formats, but are more difficult to conceptualize when used for qualitative analysis in the later stage [28]. The semi-structured interviews set the topic and guide, and the interview guide covers the main topic of the study [29]. It offers a centralized structure for free discussion during the interviews [30]. Semi-structured interviews are a common data collection method in qualitative research and the quality of the interview guide fundamentally influences the results of the study. An interview guide helpes to keep the interviews focused on the topic of the research. The form of a semi-structured guide have a certain degree of flexibility and freedom [31]. The interviewer can adjust the way and order of questions according to the actual situation during the interview [32], so as to obtain the personal feelings and thoughts of the interviewee [33], thereby generating new concepts. Semi-structured interviews typically adopt the traditional face-to-face interactions to accurately capture the participants’ emotional and subtle physical interactions to ask follow-up questions better. With the development of technology, the interviews are no longer limited to face-to-face interaction and have expanded to online communication, including telephone [34] and video chat [35]. For example, Paul suggested using Skype as an interview medium that can overcome access and distance issues and get the closest to the interaction in a face-to-face interview [24].

Oerther divided the semi-structured interview guide questions into warm up questions, core questions, probing questions, wrap up questions [36]. Scott developed a semi-structured interview guide including demographics, event details, Impact from this experience [37]. Young gathered information about interviewees using the APP from 4 directions: intervention characteristics, individual characteristics, inner setting, and outer setting [38]. The consolidated criteria for reporting qualitative research (COREQ) checklist was used to guide design, analysis, and reporting of qualitative results [39]. However, scientific research on semi-structured interviews has focused on the healthcare field, whilst interviews for furniture or product design have no fixed structure. That is, the researcher designs the interview outline by himself. For example, Knauf researched lightweight furniture in terms of general questions (price willing to pay, frequency and location of purchase, demographics), attitudes toward lightweight furniture and materials, and evaluation of materials from multiple perspectives [40].

Grounded theory was proposed by American sociologists Glaser and Strauss in the 1960s [41]. Grounded theory is not an entity theory, but a research path and methodology. Its research aims to generate, develop, and test theories from empirical data, rather than to describe and explain research phenomena [42]. Before conducting grounded theory data analysis, it is necessary to rely on observation and interaction to get relevant textual information about the research problem. It constructs concepts through qualitative coding analysis, induction and synthesis, then generalizes the conceptual categories to obtain theoretical models [43, 44]. It bridges original data and research results, providing a complete set of methods and steps for researchers to generalize and construct theories from raw materials. This theory offers a deeper and more scientific analysis of the influencing factors of customized furniture design. The grounded theory process consists of 3 steps: open coding, axial coding, and selective coding.

### Construction of 4E interview-grounded theory model

This paper adopts the semi-structured interview and coding method to construct the 4E interview-grounded theory model in an exploratory way. The 4E interview guide is to conduct semi-structured interviews with interviewees from 4 dimensions: essential information, information extraction, user experience, product expectation.

Essential information is to understand demographics of the participants such as gender, age, and occupation of interviewees [37]. Essential information often appears as the first few questions in the interview or questionnaire so that the interviewer can understand the interviewee’s background. Information extraction refers to the user’s understanding of the product and the way, such as the way to obtain product information, the degree of understanding of the product’s functions. In this era of information explosion, there are various ways for users to obtain information. The ways of accessing product information also differ in the degree of trust in the products [45]. The degree of understanding of product features also influences user demands and product selection to a certain extent [46]. User experience refers to the emotional change of information brought by the user when using and interacting with the product. Norman first proposed the concept of user experience, which has attracted extensive attention from scholars and business circles [47]. It has gradually developed from the initial computer industry to all walks of life. User experience refers to users’ intuitive feelings and feedback when using products, including not only physical and sensory feelings but also emotional and psychological feelings of users in the experience process [48]. Zhu took the government service mobile application as the main body, interviewed the user experience-related issues and formed the evaluation index system of the user experience of the government service mobile application [20]. Product expectation refers to the user’s deeper expectation and demand for the product’s function, material, style and production process. Shiba [49] raised five questions to obtain customer needs: 1)The association when customer using the product. 2)The problems that customer will encounter when using the product 3) The criteria that customer will consider when buying the product. 4) New features that better meet customer expectations. 5) Changes that customer will make to the product. Questions 1 and 2 refer to the user experience. Question 3 refers to the level of understanding of the product in information extraction. Questions 4 refer to the product expectations. Question 5 is not addressed in this study.

**Fig 1:**
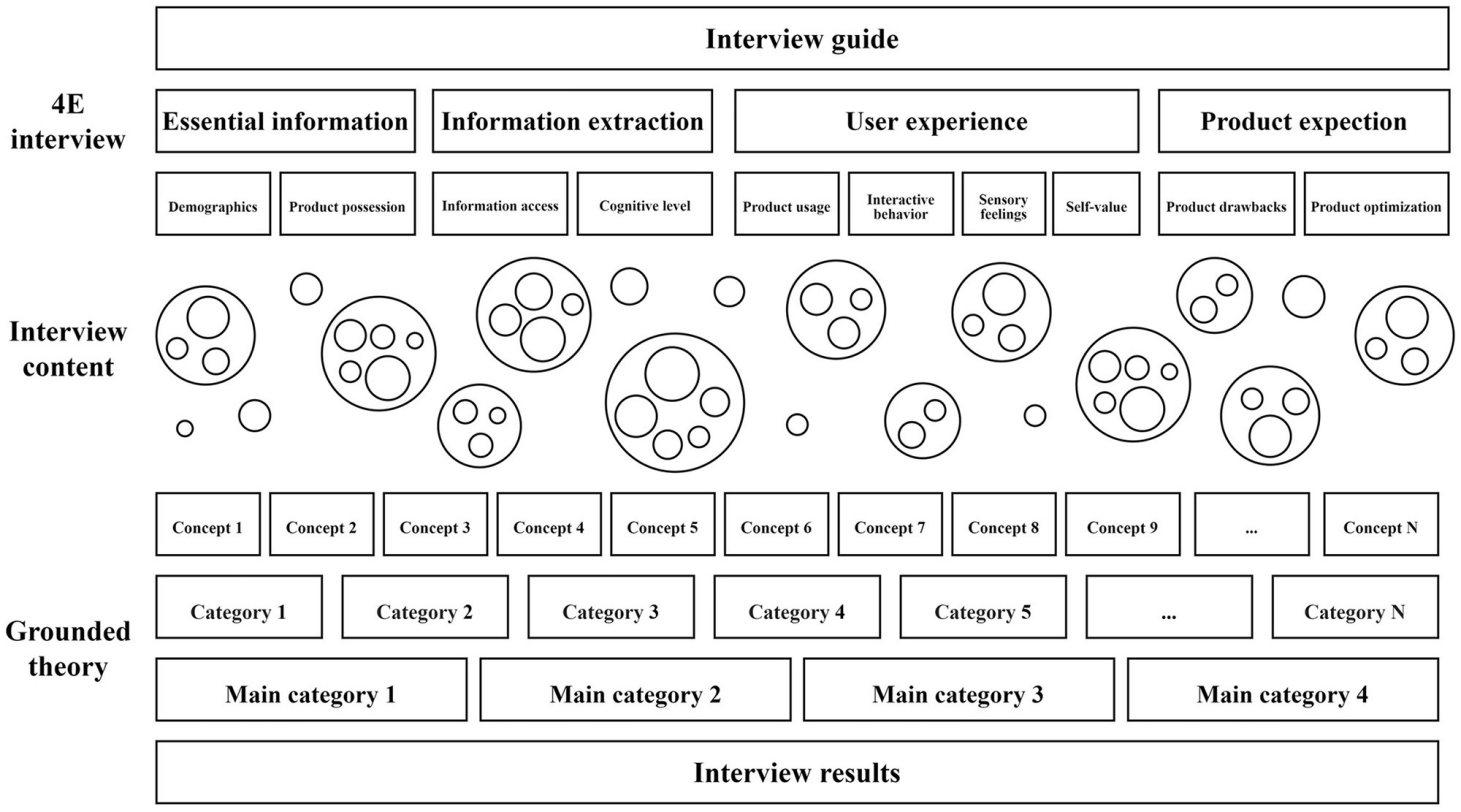
Illustration of 4E interview-grounded theory model.

The 4E interview guide was able to structure and organize the interview guide of the factors influencing the demand for customized furniture to ensure the integrity of the interview content. The initial coding and initial categories derived from the 4E guide have strong logic and adopted the grounded theory as the analysis method of the 4E interview guide interview text, as shown in Fig 1. The 4E interview-grounded theory model consists of 2 modules: 4E interview and grounded theory coding. 4E interview module is subdivided into 4 dimensions, mainly from 4 aspects: essential information, information extraction, user experience, and product expectations to obtain user information and product demand. The grounded theory coding module mainly processes the collected user information and product requirements. It will conduct data cleaning and coding and finally obtain the constituent factors and mechanisms that affect the user demands for customized furniture.

### Research process

**Fig 2:**
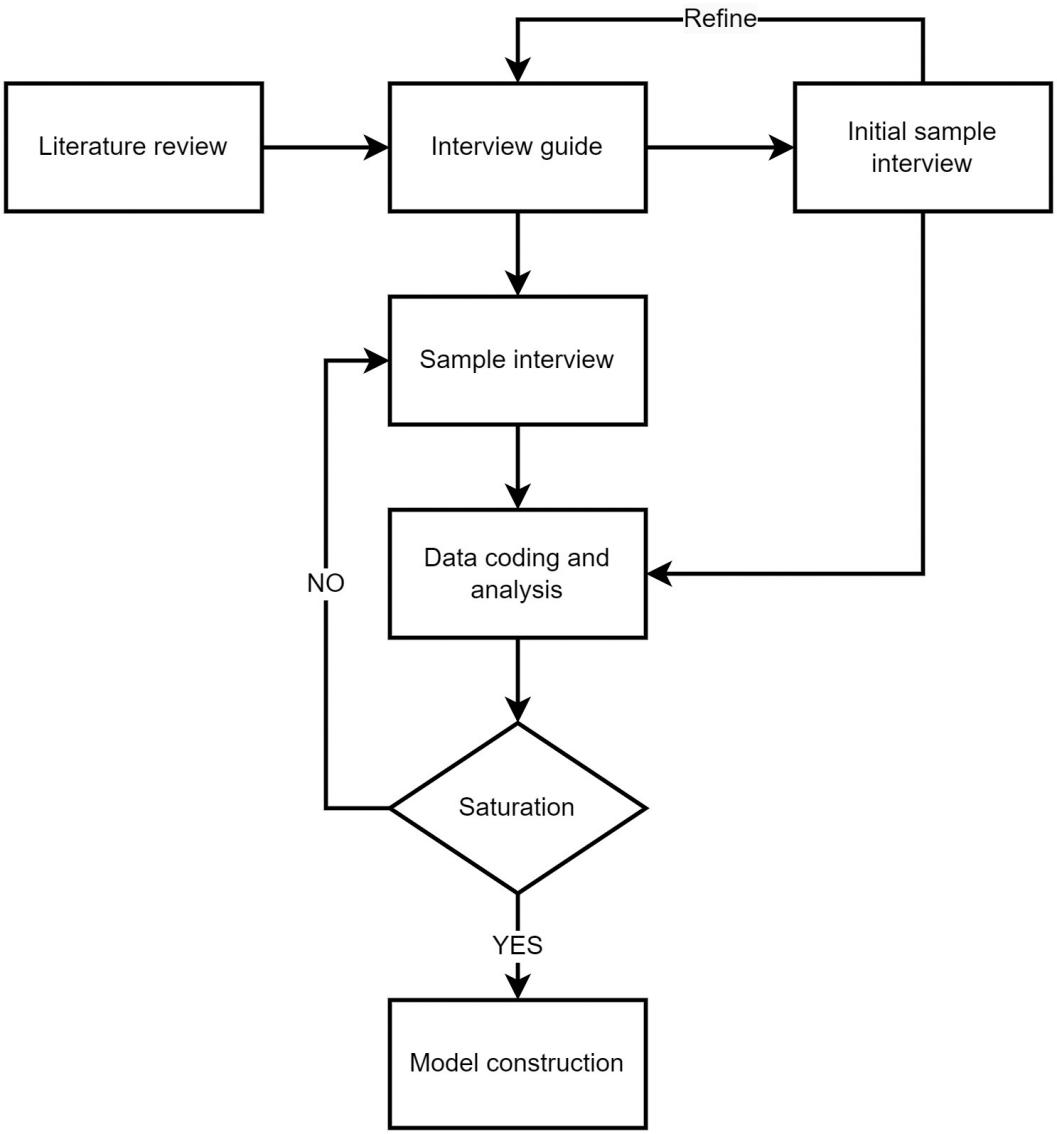
Research process.

Under the guidance of the 4E interview-grounded theory model, the research process of this study is conducted in the following 4 steps, as shown in Fig 2.

1. At the beginning of the study, the researcher searched relevant papers and analyzed current customized furniture cases to summarize other researchers’ interview questions.
2. Deepen the semi-structured interview questions based on the 4 dimensions in the 4E interview guide.
3. 2 interviewees were selected for the field sample interview test. The interview guide was adjusted and refined according to the interviewees’ responses, as shown in Table 1, which contains main themes and follow-up questions. The main themes grasp the development direction of the interview content, and the follow-up questions are used to gain an in-depth understanding of users’ thoughts and guide users who are too divergent to return to the main themes [50].
4. Interview 36 consumers who have purchased customized furniture or need to purchase customized furniture in recent six months. With the consent of the interviewees, the interview process was recorded. The interview places were the home, studio, or lounge of the interviewees with reasonably control environmental interference. This study has been approved for ethical review by the Science and Technology Office of Nanjing Forestry University. At the same time, all participants signed a written informed consent form before the interview, which included the basic information of this study, the right to voluntary withdrawal and the financial reward.
5. The results of the interviews were coded and analyzed by 2 researchers, and new concepts and categories were collected in a continuous induction process.
6. Saturation tests were conducted on the coding results to construct a model of the influencing factors of customized furniture. In this study, the principle of “theoretical saturation” of grounded theory was followed in the sampling process. In other words, when no new initial concepts, new categories and path relations appeared in the coding process of the original text data of the subsequent interviewees, the primary and secondary categories were determined to be complete and passed the theoretical saturation test.

**Table 1:** Customized furniture 4E interview guide.

Dimension	Main Theme	Follow-up Question
**Essential information**		
Demographics	How old are you?	
	What is your education and occupation?	
	What is the area of your house?	What is the house type like?
	How many people are in your family?	What is the family structure?
Product possession	Where is your customized furniture placed?	What things are mainly used to put?
	What is the style of customized furniture in your house?	
	How much did you spend on customized furniture?	How much does it cost to customized furniture in different spaces?
**Information extraction**		
Information access	What were your initial understanding channels about the customized furniture brand you chose?	What was your initial impression?
	How do you learn about customized furniture at present?	
Cognitive level	Do you care about the brand benefits of your products?	Did this influence your choice of customized furniture brands and styles?
	What do you think you should pay attention to when choosing customized furniture?	
	What are the advantages of customized furniture compared to finished furniture?	Why do you choose customized furniture?
**User experience**		
Product usage	How often do you use cabinets, wardrobes, and other customized furniture?	
	How ranked are your satisfaction with the use of all customized furniture in your home?	What are the reasons for satisfaction or dissatisfaction?
Sensory feelings	Does the current customized furniture meet your needs for product appearance?	Which appearance details meet your needs? Which needs are not being met?
	Does the current customized furniture meet your needs for product tactility?	Which tactile details meet your needs? Which needs are not met?
	Does the current customized furniture meet your needs for product functionality?	Which functions meet your needs? Which needs are not met?
	Does the current customized furniture meet your needs for product audibility or smell?	
Interaction behaviour	What are your main actions when using customized furniture?	
	What are the ways to open and close the doors of your customized furniture cabinets?	Which opening and closing method do you prefer? Why?
Self-value	Did the experience of customized furniture affect your self-recognition mood?	
	Did you share your successful renovation experience with others?	
**Product expectation**		
Product drawbacks	What are the shortcomings of the current customized furniture?	
Product optimization	What will the living space of the future look like?	
	What other functions can be added to customized furniture?	
	What aspects of customized furniture can provide users with more possibilities?	

### Participants

A total of 36 respondents were interviewed for this study, half of whom were male and half female. In order to make the interview results more typical, this study selected people with different occupations and levels of education. The interviewees ranged from 20–55, including 32 young people aged 20–35. This is consistent with the fact that China’s post-90s and post-85s rank first and second in preference for customized furniture in 2020, as mentioned in the Weekly Report of Light Industry and Textile Garment Industry [51].

## Coding process

### Open coding

The text description information of the interview materials is coded sentence by sentence in the open coding stage, which is a process of categorizing the interview content [52]. The first 28 interview materials were chosen for coding in this study. As shown in Table 2, 51 concepts and 19 categories were obtained through concept comparison and recombination, including price factors, ease operation, and quality of service.

**Table 2:** Partial open coding process.

Interview Dimension	Primary Data	Concepts	Category
**Essential information**	We customized 3 cabinets, one kitchen cabinet and 2 closets, and spent almost 60,000 RMB in total, a little higher than our initial budget.	Product price Cost-effectiveness Decoration budget Promotion	Price factors
	My family has 4 members, and the house is about 1000 square feet.	House area Family structure	Space size
	I like simple style, so I decorate my house in this style.	Personal preference	Style preference
	…	…	…
**Information extraction**	At first, I heard about OPPEIN on TV commercials, then I got an impression of the brand and thought it was a big brand and trustworthy.	Brand value Influence	Brand effect
	When choosing customized furniture, we went to their offline store to experience it. We consulted with friends who had already decorated, and then we went through the search engine and WeChat public number to learn about the information.	Advertising Offline activities Acquaintance recommendation	Publicity recommendation
**User experience**	At that time, we carefully chose the formaldehyde-free board to prevent formaldehyde from affecting our body’s safety. For children’s sake, the furniture corners are required to be rounded and smooth, and the drawers are selected with damping slides to prevent touching.	Material safety Drawer bumping Corner touching	Safe operation
	When we customize the cabinets, we choose materials that are easy to clean to prevent oil penetration.	Easy to clean	Convenient operation
	There are children in the family, so the cabinet doors need to be easy to open and close, easy to operate and convenient for storage.	Easy to open and close Easy to store	Ease operation
	I hope the furniture material can resist deformation and the hardware is durable.	Material deformation resistance Hardware durability	Product durability
	The main reason for choosing OPEIN is its appearance and the texture of the panels.	Beautiful modeling Natural pattern	Visual perception
	…	…	…
**Product expectation**	Customized furniture can add some functions such as antivirus sterilization, auxiliary lighting, diet matching and clothing matching.	Auxiliary lighting Intelligent matching Health elimination	Comfort operation
	…		

### Axial coding

In order to determine the internal logical relationships between the inductive categories and the categories created through open coding, the main axial coding is used [53]. As indicated in Table 3, the major axial coding is done based on the results of open coding to create the 4 main categories of fundamental condition, operation behaviour, sensory value, and emotional value.

**Table 3:** Partial axial coding process.

Main category	Category	Explanation
Fundamental condition	Price factors	The fit between product price and user budget
	Space size	Determinants of the external dimensions of customized furniture
	Style preference	Significant factors influencing the appearance and style of customized furniture
	Cultural literacy	Factors affecting the user’s personal characteristics and demands
	Brand effect	Brand influence, brand communication and brand recognition
	Publicity recommendation	Measures to attract users for in-depth understanding or offline experience
Operation behaviour	Safe operation	Health and life safety of users when using customized furniture
	Convenient operation	Simple procedures to improve operation efficiency
	Ease operation	Can be operated by all age groups without complicated teaching
	Product durability	Can be utilized for a very long period and has a lengthy service life
	Comfortable operation	Ergonomic and positive user experience
Sensory value	Visual perception	User’s perception of the appearance, form and color of the furniture
	Smell perception	User’s perception of the odor emitted by the furniture
	Auditory perception	User’s perception of the sound produced when operating the furniture
	Tactile perception	Feedback from hands and bodies touching furniture
	Space utilization	The layout and storage of the interior space of customized furniture
Emotional value	Self-satisfaction	Recognition of product quality, self-recognition and pleasure during use
	Emotional resonance	Brand culture and stories arouse emotional resonance of users

### Selective coding

**Fig 3:**
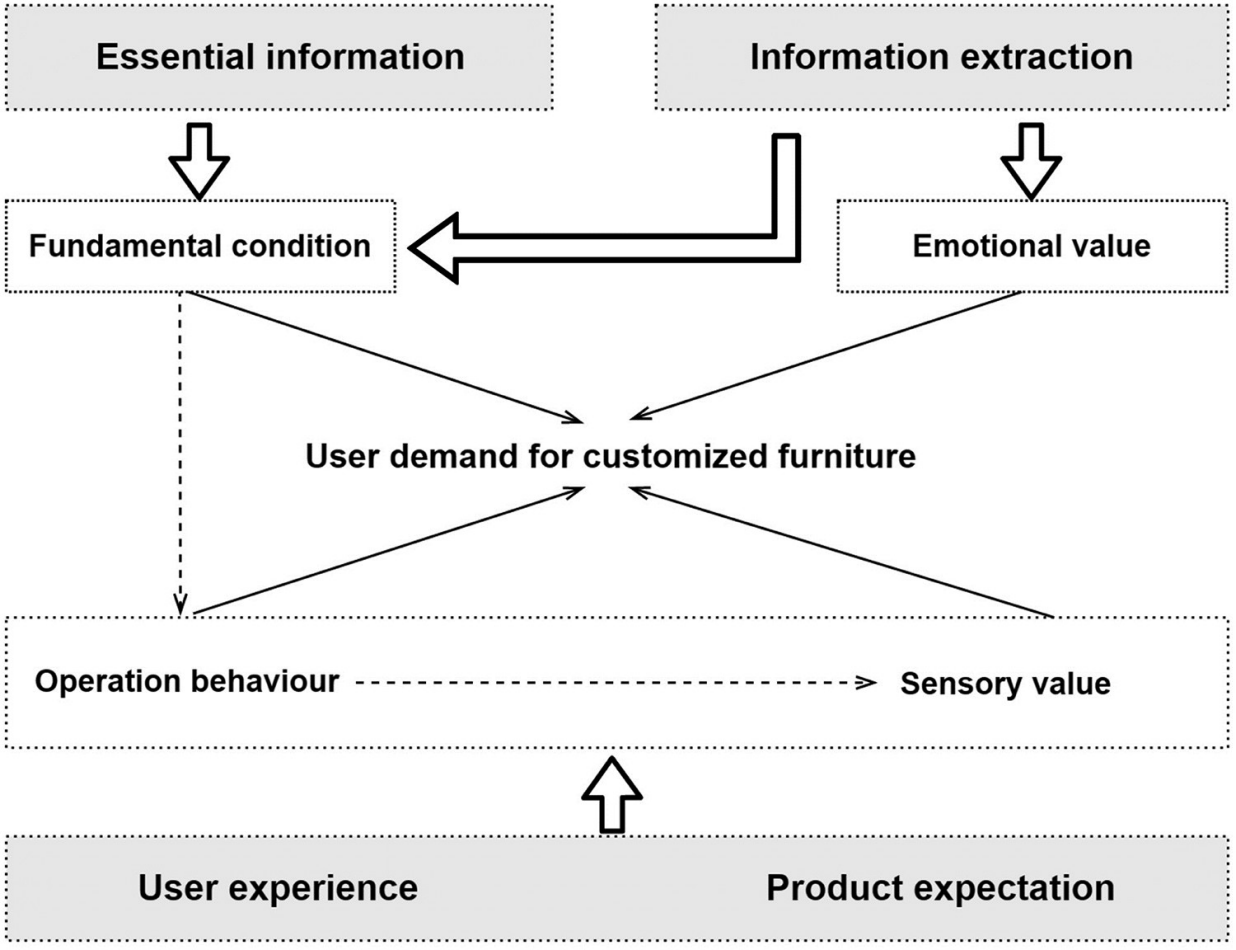
Demand factors for customized furniture.

The primary goal of selective coding is to separate the core category from the main categories. The core category of factors influencing user needs of customized furniture is finally extracted through further analysis, comparison, and induction of the 4 main categories obtained from axial coding [53]. The goal of selective coding is to create a story line that describes the relationship between the central category and each main category, as shown in Fig 3.

### Theoretical saturation test

Theoretical saturation test refers to the point at which fresh theoretical understandings and test categories can no longer be gleaned from recently gathered data [54]. The study found that no new concepts, categories and relationships were found in the follow-up analysis of the materials of the 29th interviewee. As a result, it is determined that the theoretical research model has reliability and has reached the theoretical saturation state.

## Findings

Based on 4E semi-structured interviews and grounded theory, this study reveals that user demand for customized furniture is mainly influenced by 4 factors: fundamental condition, operation behaviour, sensory value, and emotional value.

### Fundamental condition

Through the 3-layer coding based on grounded theory, the sources of fundamental condition can be divided into essential information and information extraction. The fundamental condition obtained from the essential information of user interviews include age, occupation, area, style preference, etc. User essential information plays a vital role in customized furniture design. On the one hand, it is the externalization of the user’s personal needs; on the other hand, it acts as a “filter” of the vast amount of resources and target resources. The fundamental condition derived from information extraction are of a specific social nature. The direct impact of information extraction on customized furniture design is mainly reflected in the social appeal of the internal environment, the display and promotion of the external environment. The social demand of the internal climate mainly refer to the social environment in which the user lives, such as interpersonal relationships, social interaction. The promotion display of the external environment mainly refers to all kinds of media display and platform promotion, which are all kinds of print and video advertisements or promotional information appearing on search engines [55].

The research findings show that: 1) When the recommended information is extracted from strong relationships (friends and family), it will directly stimulate users to experience and purchase offline. 2) When the recommendation information comes from media display or weak relationship recommendation (e.g. forum, twitter), it will stimulate users’ desire for in-depth understanding and indirectly guide users to conduct online and offline brand product queries and other activities. If the information matches the user’s fundamental information, it increases the likelihood that the user will buy. Conversely, when the user’s essential information does not match the enterprise sales positioning, the user chooses to save time costs and does not continue to learn more. 3) When the user’s fundamental condition matches the positioning of the enterprise and products, the possibility of the user’s purchase is greatly increased. Price matching is the most critical factor.

Therefore, the matching degree between the data obtained by users and users’ fundamental condition determines users’ choice of customized furniture. For example, when the product price matches the user’s affordable price, users are most likely to have a benign attitude toward the enterprise and experience the purchase in depth. At the same time, if the information extraction comes from the strong relationship of users, it will greatly increase users’ probability of buying such products.

### Operation behaviour

Safe operation, ease operation, product durability and comfortable operation are all included in the category of operation behaviour. All customized furniture operations are predicated on safety, with simplicity of use serving as the fundamental building block, durability of the furniture serving as a guarantee, convenience serving as the core value, and comfort serving as the end aim. Therefore, the operation behaviour is closely related to the user’s functional requirements, which is the consideration of product practicality. User operation behaviour reflects the unique qualities of the user and is highly correlated with usage patterns and prior experiences [56]. That is, the behaviour is generated based on existing cognitive guidance before using the product.

During the interview, more than 90% of the respondents paid close attention to the safety performance of customized furniture. The coded content of the interviews by grounded theory led to the following findings: 1) The influence of the operation behaviour on the demand of customized furniture users is mainly divided into 3 aspects: the user’s physiological condition (height or weight), user habits, and on-site user experience. 2) The on-site user experience is the main factor affecting the user’s choice of customized furniture. For example, when users choose customized kitchen cabinets, they usually choose the kitchen environment of the offline showroom to experience and simulate the cooking process to judge the comfort of the operation. 3) The operation behaviour can directly influence the user’s demand for customized furniture and act as an intermediary factor to trigger the user’s sensory experience to influence the demand.

### Sensory value

The furniture contains the connotation of a multi-level sensory system. All the sensory and associative factors related to furniture constitute the complete feeling of a piece of furniture. Vision is the primary source of perception of external things. The visual perception of the product is related to the appearance, color and texture of the customized furniture. Beautiful customized furniture can not only attract users’ attention and bring pleasant feelings, but also stimulate users’ enthusiasm for using furniture. People’s instinctive response to furniture is not just about visual perception, but also the combined appeal of touch, hearing, and smell. When using customized furniture, the material texture and surface finish directly affect the user’s tactile experience. The tactile sensation of the furniture is related to the material texture and surface treatment process. The furniture material is friendly to touch, and this comfortable and pleasant feeling will bring an excellent emotional interaction. The smell or sound emitted by customized furniture is often related to the safety of the furniture panels. For example, the sound brought by the furniture when it is not used smoothly will make consumers think that the product technology is not up to the standard and that there are potential safety hazards. Customized furniture design needs to focus on the integration of multi-sensory to meet users’ living needs and improve user satisfaction. Therefore, the user’s sensory experience determines the user’s demand for the appearance and color, material & finishing of customized furniture.

The findings of the research show that: 1) Based on the ranking choices of the respondents, visual perception is the most influential factor affecting the purchasing power of users in the sensory value. The visual impact often leaves a first impression on users. During the interview process, it was found that the first impression quality can most affect subsequent users’ desire for in-depth experience and purchase demands. 2) The tactile sense is also one of the decisive factors influencing users to purchase customized furniture products. Chinese consumers prefer wooden customized furniture and the warm touch.

### Emotional value

Emotional value significantly affects the loyalty, reputation and popularity of customized furniture brands. Based on meeting the fundamental requirements, functional needs and sensory experience of users, it is also important for customized furniture to give emotional value to users. The story behind the customized furniture can arouse the emotional resonance of users. The furniture is used as a medium to convey the brand culture and life philosophy to users. The cultural value and attributes of the product are further transmitted through emotional communication so that users can quickly receive the cultural information and emotional value conveyed by the product. So as to establish a good brand image, create a brand effect, increase user goodwill and viscosity, and improve user purchase probability. Research findings show that brand or product story and brand effect can influence users’ choice of customized furniture. A good brand and product story can give users a deeper understanding of the brand and its products and attract customers’ attention.

## Discussion

This research found that the demand of users for customized furniture is mainly affected by 4 factors: fundamental condition, operation behaviour, sensory value and emotional value. Our research shows that when the user’s affordability matches the selling price of a product, the user is likely to have a positive attitude toward the product and have a purchase intention. This finding was also reported by Chun and Nyam-Ochir (2020) [57], Ryu and Han (2010) [58], Liu et al. (2020) [59], who proposed that price affects consumer satisfaction. In addition, our study finds that user acceptance of a product or brand is influenced by affinity. When the recommendation information originates from a strong relationship, users are likelier to go for the brand’s products. This finding is consistent with Breitenbach and Brandão (2019) [60], Cui et al. (2019) [61].

Previous studies have reported the relationship between user habits and user behaviour. Liu et al. (2022) [62], Hagger (2020) [63], Zhang et al. (2022) [64] proposed that user operation behavior is affected by user habits. Based on previous research results, we creatively point out that user operation behaviour mainly affects user demand from 3 aspects: user physiological condition, user habits, and on-site user experience. Our analysis shows that the impact of the on-site user experience is the main factor in the user’s choice of customized furniture, which is similar to Srivastava and Kaul (2016) [65], Cachero-martinez and Vazquez-casielles (2017) [66], Hou and Jiang et al. (2021) [67].

Sensory value is generated by users in the process of use and experience, which affects users’ demand for products. The research findings support the results proposed by Cachero-martinez and Vazquez-casielles (2017) [66], Andrew (2013) [68], who believe that sensory value is an essential factor affecting shopping satisfaction. The visual senses are the most influential factor in the sensory value that affects the user’s purchase. This result is consistent with the findings of Mugge and Schoormans (2012) [69], Artacho and Alcántara and Martínez (2020) [70]. Artacho (2020) [70] suggested that vision is the dominant organ when purchasing tiles, followed by touch, which is consistent with our findings.

Ryu et al. (2019) [71], Huang and Guo (2021) [72], Hong et al. (2022) [73], Huang et al. (2022) [74] proposed that there is a positive correlation between the brand story and brand image, brand trust. Graser and Reisinger (2021) [75] points out that product story is associated with brand attitude and product choice. These findings are similar to our viewpoint that brand or product story and brand effect can influence users’ choice.

In response to the above findings, we suggest that customized furniture enterprises should start from 2 levels of publicity and product design to meet user demands and increase purchase probability. Customized furniture enterprises should fully utilize the benefits of media communication and influence to develop a distinctive publicity mechanism. They should also convey the brand concept and culture to users and trigger the emotional resonance of consumers. Investigate cross-border integration and other multi-level, multi-channel, and multi-mode publicity modes, increase brand exposure and popularity to attract customers’ attention [76]. At the same time, for the target users of the brand, multi-channel distribution of content is carried out according to the user’s preferences to improve the promotion efficiency. Furniture enterprises should guide users to build corresponding social circles and utilize solid connections for community marketing to provide reciprocal recommendations between friends and family. At the level of product design, designers should take ergonomics as the design method under the premise that the product meets the fundamental functions, focus on the visual and tactile characteristics of the product, and enhance the user experience during the use of the product. In customized furniture design, enterprises need to use the beauty of form and the laws of shape to design furniture elements, so as to achieve the proportion of form as well as color harmony. At the same time, designers can grasp the trend of users’ behaviour through observation or interviews and use it for personalized services. Through continuous training, induction and fusion to achieve demand integration and establish an operation behaviour demand library [77]. Strengthen the connection between user demands and product values to form product features and brand recognition. Design according to the user’s operation behaviour to improve the user experience while using the product. At the same time, the user’s following behaviour can be predicted from the perspective of the entire operation behaviour. When users have clear behaviour goals, it assists users in acting efficiently. Such as intelligent design, in line with the premise of user behaviour habits, embeds intelligent equipment to reduce the operation process or automatic operation so as to reduce the user’s operation load.

## Conclusion

The 4E semi-structured interview guide constructed in this research conducts interviews with users from 4 dimensions: essential information, information extraction, user experience, and product expectation. The interview guide can be used for both the study of furniture and of other daily necessities. The interview content is combined with grounded theory to mine user demends and draw the following conclusions:

1. The factors that influence the demand of customized furniture users are: fundamental condition, operation behaviour, sensory value and emotional value.
2. The matching degree between the data obtained by users and users’ fundamental condition determines users’ choice of customized furniture. If the information extraction comes from the strong relationship of users, it will significantly increase the purchase probability.
3. The influence of the operation behaviour on the demand of customized furniture users is mainly divided into 3 aspects: the user’s physiological condition, user habits, and on-site user experience. Among them, the on-site user experience is the main factor affecting the user’s choice of customized furniture.
4. Visual perception is the most influential factor affecting the purchasing power of users in the sensory value, followed by tactile perception.
5. Brand or product story and brand effect will affect the user’s choice of customized furniture.

This study contributes to the follow-up research in different ways. First, this study provides a 4E semi-structured interview guide, which is conducted from 4 dimensions: essential information, information extraction, user experience, product expectation. This guide provides a reference for the demand acquisition of furniture or other products. Second, this paper combines the grounded theory with the 4E interview guide, constructs a 4E-grounded theory model to study user demand affecting customized furniture, and provides a new methodology for qualitative research. Third, this study focuses on analysing consumer demand influencing factors of customized furniture, proposes 4 factors, and points out the interaction mode and mechanism. It provides a basis for subsequent research and supplements the demand theory of customized furniture. At the same time, it also provides a theoretical reference for customized furniture enterprises to conduct demand mining and product design.

There are several limitations in this study, which may be considered in future research. First, this study adopts the qualitative research method to construct a consumer demand influencing factor and mechanism for customized furniture. However, the results of semi-structured interviews were inevitably influenced by the habits and preferences of the interviewers. Since purely qualitative research can only be used for theory construction, the generalizability and applicability of the findings need to be tested in subsequent quantitative research. In the future, the 4E interview guide will be combined with the quantitative analysis to measure the factors affecting user demands and ensure the accuracy of the research. Second, this study only examined the factors affecting user demand from the user’s perspective and did not explore the macro perspective such as economy and industry. Therefore, incorporating national economic and industrial policies into our model would be another research direction. Third, the users we interviewed were limited to China, and we cannot be sure whether our findings are applicable to other countries or regions. Therefore, the applicability of the findings in other cultural contexts will be further verified in future research work.

## Supporting information

S1 File (ZIP)
