# Supplementary material for: Building a 4E interview-grounded theory model: A case study of demand factors for customized furniture
Source: PLoS One. 2023 Apr 27;18(4):e0282956. doi: 10.1371/journal.pone.0282956 (PMC10138260; doi:10.1371/journal.pone.0282956)
Supplement: S1 File — (ZIP) [file pone.0282956.s001.zip › transcript/transcript 001.pdf]

Informant : 001

Please note that the original transcript is in Simplified Chinese. The English translation is for internal communication among the author of this research, and it is not proofread. Potential linguistic errors may exist in the English translation.

Researcher

Thank you for your willingness to participate and be interviewed here. My name is XXX, and I'm a PhD student in the XXX University. Currently, I am working on a research project that focuses on collecting information about user demand when purchasing and using customized furniture. Throughout the interview, I will ask you a series of questions and you are encouraged to express your opinions and views freely. During the interview, I will ask you if I have questions about what you have said or if I need you to clarify a topic or concept.

感谢您愿意参加并在此接受采访。我叫XXX，我是家居设计与工程专业的研究生，现在我在XXX大学就读。目前，我正在开展一个研究项目，主要收集在使用定制家具时的用户体验资料。在整个访谈中，我会问您一系列问题，我们鼓励您自由表达您的意见和观点。在访谈过程中，如果我对你所说的内容有疑问或需要您澄清一个主题或概念，我会向您询问。

Researcher

Are you ready?

您准备好了吗？

Informant 001

Yes.

准备好了。

Researcher

First, some questions about yourself. How old are you now?

首先是关于您个人的一些问题。请问您现在的年龄是多少？

Informant 001

I am 27 years old.

我今年27岁。

Researcher

What kind of work are you doing now?

请问您现在从事什么工作呢？

Informant 001

It's something to do with design.

是和设计有关的工作。

Researcher

The nature of your work should require a lot of high quality products in your life to meet your aesthetic needs.

那您的工作性质应该会在生活中需要很多高质量的产品来满足您的审美需求。

Informant 001

Yes.

是的。

Researcher

good.Do you have a house now?

好的。那请问您现在是否有房子呢？

Informant 001

Just got my own place last year.

去年刚有自己的房子。

Informant 001

We are going to live a family of four in this 102m^2 house.

我们准备一家四口一起住在这个102m^{2}的房子里。

Informant 001

I was so busy with work last year that I just got around to decorating recently.

去年工作太忙，最近才有空开始装修。

Researcher

Have you selected the decoration style?

已经选定装修风格了么？

Informant 001

It's probably settled. The overall decoration style is more prone to simple style.

大概已经确定了。整体的装修风格更偏向简约的风格。

Researcher

Ok. Would you consider custom furniture when decorating?

好的。请问您在装修时会考虑定制家具么？

Informant 001

Yes.

是的。

Researcher

Ok.What is your custom furniture style? Is it consistent with the home decor?

您家定制家具风格是什么样？和家中装修风格一致吗？

Informant 001

Basically the same, are biased towards simple style.

基本一致，都偏向简约的风格。

Researcher

Where do you keep your custom furniture?

您家的定制家具放置在哪里?

Informant 001

Mainly in the kitchen and bedroom.

主要是放在厨房和卧室中。

Researcher

Which cabinets are the main ones.

主要是哪些柜体？

Informant 001

I have three custom cabinets so far, with one cabinet and two closets.

我目前就定制了3个柜子，有一个橱柜和两个壁橱。

Researcher

How much would you spend on custom furniture?

您会花多少钱在购买定制家具上？

Informant 001

It came to nearly 60,000 yuan, a little over our original budget.

总共花了近60000元,，略高于我们最初的预算。

Researcher

Will you continue to buy other custom furniture in the future?

后续还会继续购买其他定制家具么？

Informant 001

Yes.

应该会。

Researcher

Sometimes I always want them, but I haven't bought them.

您购买的定制家具品牌是什么？

Informant 001

A brand called OPPEIN.

一个叫OPPEIN的品牌。

Researcher

How did you get the information about this brand?

您是从什么渠道了解到有关这个品牌信息的呢？

Informant 001

Friends who have bought this brand also say it is good. I learned more about him through Baidu and the official account.

买过这个品牌的朋友说还不错。我就通过百度和公众号了解到了有关他的更多信息。

Researcher

How did you get the information about this brand?

您对您选择的品牌最初印象是什么?

Informant 001

I saw OPPEIN ads on TV and thought it was a big brand and trustworthy.

我在电视上看到OPPEIN广告，认为这是一个大品牌，值得信赖。

Researcher

What is your experience with custom furniture now?

您认为相比成品家具，定制家具的优势是什么？

Informant 001

Custom furniture will make my furniture more personal.

定制家具会使我的家具更加个性化。

Informant 001

Can very well fit my space structure, to meet my life needs.

能很好适配我的空间结构，满足我的生活需求。

Researcher

What do you think you should pay attention to when choosing custom furniture?

您觉得在选择定制家具时应该注意什么问题？

Informant 001

Functional fitness to the environment.

与所处环境的功能适配度。

Informant 001

Whether it is environmentally friendly, healthy, cost-effective and in line with their aesthetic needs. All the things I think I need to consider when buying custom furniture.

是否环保健康，经济划算并符合自己的审美需求。都是购买定制家具时我觉得需要考虑的。

Researcher

How often do you use cabinets, closets, and other custom furniture?

您使用橱柜、衣柜、和其他定制的家具的频率是如何的？

Informant 001

It's used every day.

每天都在使用。

Researcher

What is the first factor you consider when buying these custom furniture? Is it the appearance?

当时您在购买这些定制家具最先考虑的因素是什么呢？是外观么？

Informant 001

Appearance is important. But my first concern is the safety of the materials used.

外观是很重要。但是我首先考虑的还是所用材料的安全性。

Researcher

Like what?

比如说呢？

Informant 001

When we bought custom cabinets, we chose formaldehyde-free panels. It has no effect on the body. Healthier.

当时购买定制橱柜的时候我们就选择了无甲醛的板材。不会对身体有影响的。更加健康。

Researcher

Ok, besides the material needs to be healthy and environmentally friendly what else would you consider when buying custom furniture?

好的，除了材质需要健康环保还有什么是您在购买定制家具时会考虑的呢？

Researcher

Is it security?

是安全性么？

Informant 001

It is indeed. Security is very important. Because I have a very small child.

确实是。安全性非常重要。因为我有一个很小的孩子。

Informant 001

He often runs up and down the house. To prevent him from getting hurt, I rounded the corners of all the cabinets. The drawers are also damped to prevent collisions.

他经常在房子中跑跑跳跳。为了防止他受伤，我就将所有柜子的边角都设计成圆角。抽屉也会加上阻尼，防止碰撞。

Researcher

good In addition to safety and environmental protection, I would also like to know whether you are satisfied with the appearance design of customized furniture at present?

好的。除了安全性和环保性这两点，我还想知道的是当前您对于定制家具的外观设计是否满意？

Informant 001

It was pretty satisfying. At that time, I participated in the whole process of designing cabinets, and I was able to communicate with designers in time to express my needs and ideas. This may be the difference between custom furniture and finished furniture.

还挺满意的。因为当时我全程参与了设计柜子的过程，能够及时与设计师沟通，说出自己的需求和想法。这可能就是定制家具与成品家具的不同吧。

Researcher

What requirements did you put forward for personalized customized furniture?

那您当时对于个性化定制家具提出了哪些需求呢？

Informant 001

Let me see. The material is healthy and safe as I mentioned.

让我想想。材质健康，安全性高刚才已经提过了。

Researcher

Yes..
是的。

Informant 001
I want to be able to meet the functional requirements that are appropriate for the environment in which the custom furniture is built.
我希望可以满足与定制家具所处环境相适配的功能需求。

Researcher
Good. Like what?
好的。比如说呢？

Informant 001
For example in the kitchen cabinets should be easy to clean. I communicated with the designer that the selection was easy to clean and could reduce or even avoid oil penetration.
比如在厨房的橱柜应该易于清洁。我就和设计师沟通希望选择易于清洁，能够减少甚至避免油渗透的情况出现。

Researcher
good Is this need for ease of cleaning being met?
好的。您说的这个易于清洁的需求现在有被满足么？

Informant 001
Yes. Compared to the cabinets I used before, they are much easier to clean.
是的。相比较我之前所使用的橱柜来说已经方便清洁了很多。

Researcher
Do current custom furniture products meet your needs with tactile details?
当前定制家具产品触觉细节满足您的需求吗?

Informant 001

Yes, I chose this material among many samples at that time. It feels like a baby's skin, smooth, delicate and easy to clean.
是的，我当时在很多样品之中选择了现在这款材质。摸起来的手感就像婴儿的皮肤，光滑细腻而且还易于清洁。

Researcher
Ok. How do you open and close your custom furniture? How do you like to open and close the door?
好的。您家定制家具开关门方式是什么样的？您喜欢哪种开关门方式？

Informant 001
Nothing special. It's usually just like normal.
没什么特别的。一般就是和正常的一样。

Informant 001
But some features can be customized to suit your needs.
不过有些功能可以根据自己需求定制.

Researcher
Ok, Like what??
好的，如说呢？

Informant 001
That is ...I made the children's closet door slightly harder to open to keep them safe.
我把孩子的壁橱门设置为稍微不易打开，保障小朋友的安全。

Researcher
So do you share your success stories with others? ??
那么你与别人分享你的成功改造经验吗?

Informant 001

Yes.
会的。

Researcher
Does the current custom furniture fit your needs for product usability or smell?
当前定制家具是否符合您对产品可用性或气味的需求？

Informant 001
It fits well. There is no noise or smell when it is used.
挺符合的，使用的时候基本没有噪音与气味。

Researcher
good What do you think are the disadvantages of custom home?
好的。请问您认为定制家具有什么缺点吗?

Informant 001
Let's see. Feeling more than a little pricey.
我想想。感觉除了价格偏贵没什么了。

Researcher
What other features do you think could be added to custom furniture?
您认为还有什么其他功能可以添加到定制的家具中吗?

Informant 001
What I want most is bactericidal function. In this way, the clothes of children and the elderly can be stacked to kill bacteria. Could we have a UV lamp inside or something.
我最想要的是杀菌功能。这样小朋友和老人的衣服叠放好就可以杀菌。在内部可以安装一个紫外线灯什么的。

Researcher

Ok. What aspects of customized furniture do you think can provide more possibilities for users?
好的。那您认为定制家具的哪些方面可以为用户提供更多的可能性?

Informant 001
Custom furniture can continue to develop in the future. It may provide some emotional value to the user.
定制家具未来可以继续发展。或许可以为用户提供一些情绪价值。

Researcher
I'm listening.
洗耳恭听。

Informant 001
Like personalized touch and smell. Meet the private needs of users.
比如触感和嗅觉上的个性化定制。满足用户的私人需求。

Researcher
What is private demand?
什么是私人需求?

Informant 001
TLike whether you prefer strawberry and frosted textures, or irregular but durable materials. You can talk to the designer about anything you want and get it done.
比如你喜欢草莓味和磨砂质感，还是不规则但耐用的材料。你想要什么都可以和设计师沟通，并能全部实现。

Researcher
These are all the questions. Thank you very much for participating in our research.
好的谢谢。这就是全部的问题。非常感谢您参与我们的研究。

Informant 001

You are welcome.

不用谢。
