# Supplementary material for: Building a 4E interview-grounded theory model: A case study of demand factors for customized furniture
Source: PLoS One. 2023 Apr 27;18(4):e0282956. doi: 10.1371/journal.pone.0282956 (PMC10138260; doi:10.1371/journal.pone.0282956)
Supplement: S1 File — (ZIP) [file pone.0282956.s001.zip › transcript/transcript 002.pdf]

Informant : 002

Please note that the original transcript is in Simplified Chinese. The English translation is for internal communication among the author of this research, and it is not proofread. Potential linguistic errors may exist in the English translation.

Researcher

Thank you for your willingness to participate and be interviewed here. My name is XXX, and I'm a PhD in the XXX University. Currently, I am working on a research project that focuses on collecting information about user demand when purchasing and using customized furniture. Throughout the interview, I will ask you a series of questions and you are encouraged to express your opinions and views freely. During the interview, I will ask you if I have questions about what you have said or if I need you to clarify a topic or concept.

感谢您愿意参加并在此接受采访。我叫 XXX，是 XXX 大学的博士。目前，我正在开展一个研究项目，主要收集在使用定制家具时的用户体验资料。在整个访谈中，我会问您一系列问题，我们鼓励您自由表达您的意见和观点。在访谈过程中，如果我对你所说的内容有疑问或需要您澄清一个主题或概念，我会向您询问。

Researcher

Are you ready?

您准备好了吗?

Informant 002

Yes.

准备好了。

Researcher

First, some questions about yourself. How old are you now?

首先是关于您个人的一些问题。请问您现在的年龄是多少?

Informant 002

I am 34 years old.

我今年 34 岁。

Researcher

What kind of work are you doing now?

请问您现在从事什么工作呢？

Informant 002

I am a construction worker.

我是一名建筑工人。

Researcher

What is the size of the house you are currently living in?

您现在所居住房子的面积是多少?

Informant 002

Construction area of 141 square meters (three rooms, one living room, one kitchen and one bathroom, The interior is a staggered structure)

建筑面积 141 平方米（三室一厅，一厨一卫，错台式结构）

Researcher

What is your family structure like?

您的家庭结构是什么样的？

Informant 002

Three people. and the relatives live separately.

三口人。亲戚们各自居住，不在一起。

Researcher

What style of furniture is in the home?

家中家具是什么样式的？

Informant 002

The bed is old-fashioned, the structure is high and low type of third class, the bed surface is the canal wood, the surface, the paint color, the head of the bed is with the bookcase of three, there is a fan below, can store clothes. The head of the bed is sliding door, can store items, and the head of the bed with a small bookcase.

Bed length: 2.2m x 1.4m

Head height: 1.4m × 1.4m × 0.028m

Bottom side of bed: 140cm×4.3cm×50cm

The bed is an old-fashioned wood frame lacquer, Boshan lacquer

床是老式的，结构是高低式三等的，床面是水渠木材、综面、大漆色，床头是带书橱的三格，下面有开扇的，可存放衣物。床的低头是推拉门，可存放物件，另外床头另加左右小书橱格。

床长：2.2 米×1.4 米

床头高：1.4 米×1.4 米×0.028 米

床底侧：140cm×4.3cm×50cm

床是老式的木结构大漆，博山大漆

Researcher

Uh-huh, are there any other pieces of furniture besides these?

嗯嗯，除了这些还有其他的家具吗？

Informant 002

Table is the old square table (eight Immortals table), square chair, strip. There are two sets of large lacquer

桌子是旧式方桌（八仙桌），方椅，条几。大漆色　　共两套

Researcher

OK, do you have cabinet furniture in your home?

OK，您家中有柜类家具吗？

Informant 002

Comprehensive wardrobe, four groups of structure, can be combined, the cabinet is about the wardrobe, the middle two groups is the bookcase. The top is four boxes, It is a detachable structure. Length 3.3 meters, width 0.5 meters, height 1.8 meters + box 0.6 meters, total height 2.4 meters. A pair of antique frames are 2.0 meters high, 1 meter wide and 3.45cm wide

综合衣橱，四组结构，可组合，橱子左右是衣橱，中间二组是书橱。顶部为四个箱子，是可拆装的结构。长 3.3 米，宽 0.5 米，高 1.8 米+箱子 0.6 米，总高 2.4 米。博古架一对　　高 2.0 米，宽 1 米，宽 3.45cm

Researcher

So where is your custom furniture placed? Which cabinets are the main ones?

那您家定制家具放置在哪里？主要是哪些柜体？

Informant 002

The closet is in the bedroom (two bedrooms).

table (square table several), four combination bookcases, in the study room.

The living room also has a TV cabinet, bogu shelf, coffee table

壁橱在卧室（两卧室）

桌子，组合书橱四个，在书房

客厅还有电视柜，博古架，茶几

Researcher

What is your custom furniture style like? Is it consistent with the decoration style of the home?

您家定制家具风格是什么样？和家中装修风格一致吗？

Informant 002

Classical Chinese style (old Chinese style), consistent with the decoration

古典中式风格（老式中式），与装修一致

Researcher

How much do you spend on custom furniture?

你花多少钱在定制家具上?

Informant 002

In 2009, it was about 20,000-30,000 yuan.

2009 年时候差不多 2-3 万元左右。

Researcher

You can't do much at that price now.

现在这个价钱可做不了什么了。

Informant 002

Yes, prices are rising very fast now.

是啊，现在物价涨得飞快啊。

Researcher

Can you tell us about your understanding of custom furniture?

您可以浅谈一下自己对定制家具的理解是什么？

Informant 002

I think that custom furniture is related to the living needs of individual families, living conditions, etc., and residential conditions. This is also directly related to economic conditions, depending on what the requirements of the family and the owner are.

我觉得定制家具这与个人家庭的生活需求、生活条件等以及住宅条件相关。这也与经济条件有直接的关系，要看家庭及主人的要求是什么。

Researcher

How did you learn about custom furniture?

您是怎么了解定制家具相关内容?

Informant 002

Family is mainly physical stores and TV commercials. I mainly visit the Internet, public accounts, and so on.

家人主要是实体店和电视广告等。我主要是网络，公众号等，实体店参观比较少。

Researcher

What was your initial impression of the brand you chose? What was the initial understanding?

您对您选择的品牌最初印象是什么？最初的理解是什么？

Informant 002

The furniture in the home is basically vintage furniture or local purchase, not famous brands, mainly affordable and beautiful.

家里的家具基本是老式家具或者当地购买，并不是著名品牌，主要是经济适用以及美观。

Researcher

Why did you choose the brand's bespoke furniture?

您选择该品牌的定制家具的原因是什么？

Informant 002

Economical and practical, beautiful and durable.

经济实用，美观，耐用。

Researcher

What do you think are the advantages of custom-made furniture over finished

furniture?

您认为相比成品家具，定制家具的优势是什么?

Informant 002

According to the actual situation of each family to customize the required size style, combined with the actual economic situation with different materials and workmanship, more humanized, but also conducive to attract different levels of customers.

根据每个家庭实际情况定制所需的尺寸款式等，结合实际的经济情况采用不同的材料和做工，更有人性化，也有利于吸引不同层次的客户。

Researcher

What do you think you should pay attention to when choosing custom furniture?

您觉得在选择定制家具时应该注意什么问题？

Informant 002

Compare more, look more, research more people who have bought the evaluation and experience.

多比较，多看，多调查已购人群的评价和经验。

Researcher

How often do you use cabinets, wardrobes, and other custom furniture?

您使用橱柜、衣柜、和其他定制的家具的频率是如何的？

Informant 002

Relatively frequent

较为频繁

Researcher

Does the current custom furniture product look meet your needs?

当前定制家具产品外观满足您的需求吗?

Informant 002

Basically satisfied

基本满足

Researcher

Does the tactile detail of current custom furniture products meet your needs?

那当前定制家具产品触觉细节满足您的需求吗?

Informant 002

satisfy

满足

Researcher

Does the current custom furniture fit your needs for product functionality? Which need is not being met?

当前的定制家具是否符合您对产品功能的需求？哪一个需求没有得到满足？

Informant 002

We are basically satisfied.

我们基本都挺满意的。

Researcher

What is the way your custom furniture opens and closes doors? Which way do you prefer to open and close doors?

您家定制家具开关门方式是什么样的？您喜欢哪种开关门方式？

Informant 002

Sliding door, ordinary roller. Push-pull type, open and close type like, depending on the

actual situation and use scenario.

推拉门，普通滚轮。推拉式、开合式都喜欢，看实际情况和使用场景。

Researcher

Will you share your renovation success with others?

您会与别人分享您的装修成功经验吗?

Informant 002

Yes, we will share our experience with relatives and friends around us who are preparing to decorate, and we will also recommend the store we bought to them.

会啊，会向身边准备装修的亲戚朋友们分享我们的经验，也会推荐我们购买的店给他们。

Researcher

What do you think are the disadvantages of current custom furniture?

您觉得当前的定制家具的缺点是什么?

Informant 002

Popularity, affordability need to continue to work hard, let more people use custom furniture

普及度，实惠度需要继续努力，让更多人用到定制家具

Researcher

What other features do you think custom furniture can add?

您觉得定制家具可以添加什么其他功能？

Informant 002

Integrated, intelligent, temperature control, lighting control. In the modern home, intelligence has tended to be popular, and various smart devices are constantly emerging. Custom furniture can also be integrated into smart devices to achieve

personalized customization to meet our needs. For example, I think it's good to equip the cabinet with intelligent lighting that can automatically sense and turn on the light.
整体化，智能化，温控，灯光控制。在现代家居中，智能化已经趋于普及，各种智能设备也在不断地涌现。定制家具也可以融入智能设备，实现个性化定制，满足我们的需求。比如，在柜子内部配备智能照明，能够自动感应开启灯光这种，我觉得就挺好的。

Researcher
What aspects of custom furniture can provide users with more possibilities?
定制家具的哪些方面可以为用户提供更多的可能性?

Informant 002
More personalized needs to meet and design. You can choose different materials, styles and designs according to your preferences and needs, so as to create a piece of furniture that perfectly meets your requirements. It can provide more possibilities and personalized design, which can meet the more refined and personalized needs of users, and create a better home environment and comfortable use experience.
更具有个性化的需求满足和设计。可以根据自己的喜好和需求挑选不同的材料、样式和设计，从而创造出一件完全符合自己要求的家具。能够提供更多的可能性和个性化的设计，可以满足用户更为精细和个性化的需求，营造更出色的家居环境和舒适的使用体验。

Researcher
Okay, thank you for receiving our interview.
好的，谢谢您接收我们的访谈。
