# Supplementary material for: Building a 4E interview-grounded theory model: A case study of demand factors for customized furniture
Source: PLoS One. 2023 Apr 27;18(4):e0282956. doi: 10.1371/journal.pone.0282956 (PMC10138260; doi:10.1371/journal.pone.0282956)
Supplement: S1 File — (ZIP) [file pone.0282956.s001.zip › transcript/transcript 003.pdf]

Informant : 003

Please note that the original transcript is in Simplified Chinese. The English translation is for internal communication among the author of this research, and it is not proofread. Potential linguistic errors may exist in the English translation.

Researcher

Thank you for your willingness to participate and be interviewed here. My name is XXX, and I'm a PhD in the XXX University. Currently, I am working on a research project that focuses on collecting information about user demand when purchasing and using customized furniture. Throughout the interview, I will ask you a series of questions and you are encouraged to express your opinions and views freely. During the interview, I will ask you if I have questions about what you have said or if I need you to clarify a topic or concept.

感谢您愿意参加并在此接受采访。我叫XXX，是XXX大学的博士。目前，我正在开展一个研究项目，主要收集在使用定制家具时的用户体验资料。在整个访谈中，我会问您一系列问题，我们鼓励您自由表达您的意见和观点。在访谈过程中，如果我对你所说的内容有疑问或需要您澄清一个主题或概念，我会向您询问。

Researcher

Are you ready?

您准备好了吗？

Informant 003

OK.

Researcher

How old are you?

您多大了？

Informant 003

35 years old.

35岁。

Researcher

What is your occupation?

您的职业是什么?

Informant 003

I am an engineer.

我是一名工程师。

Researcher

What is the size of the house you live in?

您居住的房子的面积是多少?

Informant 003

Approximately 80m^2.

大概80m^2。

Researcher

What is the size of your family? What is the family structure?

您的家庭人数？家庭结构是什么样的？

Informant 003

3 people, my husband and I, and we have a 3 year old child

3个人，我和我丈夫，我们还有一个3岁的孩子。

Researcher

Where is the custom furniture placed? Which cabinets are the main ones?

您的定制家具放置在哪里? 主要是哪些柜体？

Informant 003

我们的定制家具主要是壁柜与橱柜，都是贴墙安装，这样比较能利用空间。

Researcher

What style of custom furniture do you have?

您的定制家具是什么风格的呢?

Informant 003

More simple style, more practical and durable

比较简单的风格，比较实用且耐看

Researcher

What are your motivations for choosing to purchase a custom home rather than standard-size furniture?

您选择购买定制家居而不是标准规格的家具的动机是什么？

Informant 003

More in line with my needs.

更加符合我的需求。

Researcher

Can you tell me more?

能说得详细一些吗？

Informant 003

Is more with their own style and preference ah, pure purpose

就是更具有自己的风格和偏好呀，纯粹的目的

Informant 003

Better Match

更匹配

Researcher

How do I understand the word "better match"?

我怎么理解"更匹配"这个词？

Informant 003

It's to make you feel like you've got more needed features after spending money, such as when I need auxiliary lighting, which can be added when customizing your home.

就是让你感觉你花了钱之后可以获得更需要的功能，例如我需要辅助照明，那定制家居的时候就可以加上去。

Researcher

Good. So what do you think your initial understanding of custom homes was?

好的。那么您觉得您对定制家居最初的理解是什么呢？

Informant 003

It must meet some of my specific needs more than other furniture, have a certain sense of exclusivity, reflect my personal preferences, and reflect that this is my house.

必须相较于其他家具，要更能满足我的一些特定需求，有一定的专属感，能体现我的个人偏好，体现出这是我的房子。

Researcher

Very interesting description. Let's move on to the next topic. How did you learn about custom furniture?

很有意思的描述。我们继续下一个话题。您是如何了解到定制家具的？

Informant 003

At first, I heard the advertisement on TV, and then I had an impression of the brand and thought it was a big brand and trustworthy. Before choosing custom furniture, we

consulted friends who had already decorated, and learned information through search engines and WeChat public.
起初,我在电视上听到广告,然后我有一个对这个品牌的印象,认为这是一个大品牌,值得信赖。在选择定制家具之前我们咨询已经装修的朋友,并且通过搜索引擎和微信公众了解信息。

Researcher
So relevant branding and information dissemination channels are very important, right?
所以相关的品牌宣传和信息传播渠道是非常重要的是吗？

Informant 003
I think so.
我觉得是的。

Researcher
And do you share your custom furniture experience with others?
那您会与别人分享您的定制家具经验吗？

Informant 003
They do, and usually talk about it for a long time.
会的，并且通常以此为话题谈论很久。

Researcher
Tell us more about it?
详细讲讲呢？

Informant 003
I will share it with people around me and will tell my friends around me the pros and cons of the process of this set of custom home.
我会跟身边的人分享，会告诉身边朋友我这一套定制家居的过程中的优点和缺点。

Informant 003

It will involve many aspects, while it will be about price, quality, and all aspects of after-sales service

会涉及到很多方面，一边会有关价格、品质、还有售后服务各个方面

Informant 003

Sometimes they also share their experiences on public social media platforms.

有时候还会在一些公开的社交平台分享相关经验。

Informant 003

There will be a lot of Internet users can share together, before deciding to choose custom furniture I will also search the Internet to see the relevant experience.

会有很多网友可以一起交流分享，在决定选择定制家具之前我也会在网上搜索相关经验来看。

Researcher

Okay. Can you please describe the pros and cons of your custom furniture process this time around?

好的。能否请您描述一下您这次定制家具过程中的优缺点？

Informant 003

Overall the whole experience was more in line with what we had asked for at the beginning.

总体来说整个体验还是比较符合我们一开始提出的要求的。

Informant 003

At that time, we carefully chose the formaldehyde-free board. For the sake of children, the corners of furniture need to be rounded and smooth to prevent collisions.

那时,我们谨慎选择了无甲醛板。为了孩子,家具角落需要圆润光滑,防止碰撞。

Informant 003

Safety is the primary aspect we wish to consider, and this aspect is still relatively well accomplished, with large rounded corners on all furniture, and there will be measures to correspond to the consideration

安全是我们首要希望考虑的方面，这个方面完成的还是比较好的，所有家具都采用大圆角，并且会有对应考虑的措施

Informant 003

In addition to that we were instructed about cleaning issues and they were all addressed positively for us.

除此之外我们还叮嘱了有关清洁方面的问题，并且都给我们积极解决了。

Informant 003

To speak of the disadvantages, it may be that the cycle time is a little longer and the degree of customization is limited to a certain range, and not all conditions can be met.

要说缺点，可能就是周期长一点，并且定制程度有限，要在一定范围内，也不是所有条件都能满足。

Researcher

So that's it. So after you know the existence of custom furniture, will you go through some channels to get better information about these products?

原来如此。那么在您知道了定制家具的存在之后，会不会通过一些渠道去更好地了解这些产品的信息？

Informant 003

Will, generally will go to some official websites and WeChat public number and some public social platform to search for experience in the furniture topic

会的，一般会去一些官方网站和微信公众号以及一些公开社交平台的家具专题中去搜寻经验。

Researcher

How often do you use cabinets, closets, and other custom-made furniture?

您使用橱柜、衣柜和其他定制的家具的频率是如何的

Informant 003

Frequency is high, because the cabinet type of things are used to store items, especially the closet and the kitchen cupboard is must be used every day, but the living room decorative cabinets are not necessarily, may be three to five days once.

频率很高，因为橱柜类的东西是用来存放物品的，特别是衣柜以及厨房的碗柜是必须每天都会使用的，但是客厅的装饰类的柜子就不一定，可能三五天一次。

Researcher

Do the tactile details of current custom furniture products meet your needs?

当前定制家具产品触觉细节满足您的需求吗?

Informant 003

Not bad, because the surface materials are selected according to our preferences, comparison before finalizing, and then customized out, from the appearance to practicality, overall very satisfied.

还不错，因为表面材料都是根据我们的喜好挑选的，对比之后才最终确定，然后定制出来，从外观到实用性，总体来说非常满意。

Informant 003

I prefer simplicity, so the biggest meaning of customizing the home for me is not the appearance, but the added personalization work. What I only express is that complex activities often do not make people like them.

我更喜欢简洁的，所以定制家居对我来说的最大意义不是外观，是增加的个性化功能。我仅仅表达的是，复杂的设计往往并不让人喜欢

Informant 003

But it does not mean that the simpler the activity is, the better
但不代表越简单的设计是越好的

Researcher
So that's it. Let's move on to the next topic. What is the way of opening and closing the door of your custom furniture? Which door opening/closing method do you prefer?
原来如此。我们进入下一个话题。您家定制家具开关门方式是什么样的？您喜欢哪种开关门方式？

Informant 003
The cabinet is the way to open the door by pressing, and the closet is the way to open the door by directly pulling.
橱柜是按压开门的方式，衣柜是直接拉开开门的方式。

Resecher 003
What other features do you think can be added to custom furniture?
您觉得定制家具可以添加什么其他功能

Informant 003
But I usually do not refer to only one statement, I will compare multiple
根据不同的人群添加不同功能的，例如针对老人可能可以多一些把手，方便老人腿脚不方便的时候支撑一下的，或者还有针对一些材料特性的。

Researcher
Can you give an example?
可以举个例子吗？

Informant 003
For example, when we customize cabinets, we want to choose materials that are easy to clean and prevent oil penetration.

比如当我们定制橱柜,我们希望选择的材料很容易清洁,防止油渗透。

Informant 003

So I would ask a few more and compare the materials before going to make a decision.

所以我会多问几家，对材料进行对比，再去做决定。

Researcher

I understand.

我明白了。

Researcher

Okay. Let's talk a little bit more about some of the specific needs, what did you have in mind for your kids during the custom furniture process?

好的。我们再聊一下一些特定需求，您在定制家具的过程种有为孩子考虑到什么呢？

Informant 003

For more security

为了更安全

Researcher

Tell us about it

展开说说呢

Informant 003

Families with children, so the cabinet door needs to be easy to open and close, easy to operate and convenient to store.

有孩子的家庭,所以内阁门需要容易打开和关闭,操作方便,方便存储。

Informant 003

Meanwhile, I want furniture materials that can resist warping and hardware that is durable.
同时我希望家具材料可以抵抗变形和硬件是持久的。

Informant 003
The main reason for the final choice of the brand is its appearance and texture of the panel.
最终选择的品牌主要原因是它的外观和质地的面板。

Researcher
Good. And how about your custom furniture this time in terms of price?
好的。那您这次定制家具的价格方面如何呢？

Informant003
We had 3 custom cabinets, one cabinet, and 2 closets built for a total of nearly ¥25,000, which was slightly more than our initial budget.
我们定制的3个柜子,一个橱柜,2壁橱,总共花了近25000元,略高于我们最初的预算。

Researcher
Then you rank the current custom furniture in your home
那你对目前家中的定制家具排个序

Informant 003
We felt that the customization of the cabinets would maximize the use of space and facilitate storage.
我们觉得橱柜的定制可以最大化利用空间，方便储存。

Researcher
These are all the questions. Thank you very much for participating in our research.
这就是全部的问题。 非常感谢您参与我们的研究。
