# Supplementary material for: Building a 4E interview-grounded theory model: A case study of demand factors for customized furniture
Source: PLoS One. 2023 Apr 27;18(4):e0282956. doi: 10.1371/journal.pone.0282956 (PMC10138260; doi:10.1371/journal.pone.0282956)
Supplement: S1 File — (ZIP) [file pone.0282956.s001.zip › transcript/transcript 004.pdf]

Informant : 004

Please note that the original transcript is in Simplified Chinese. The English translation is for internal communication among the author of this research, and it is not proofread. Potential linguistic errors may exist in the English translation.

Researcher

Thank you for your willingness to participate and be interviewed here. My name is XXX , and I'm a PhD in the XXX University. Currently, I am working on a research project that focuses on collecting information about user demand when purchasing and using customized furniture. Throughout the interview, I will ask you a series of questions and you are encouraged to express your opinions and views freely. During the interview, I will ask you if I have questions about what you have said or if I need you to clarify a topic or concept.

感谢您愿意参加并在此接受采访。我叫 XXX，我是 XXX 大学的博士。目前，我正在开展一个研究项目，主要收集在使用定制家具时的用户体验资料。在整个访谈中，我会问您一系列问题，我们鼓励您自由表达您的意见和观点。在访谈过程中，如果我对你所说的内容有疑问或需要您澄清一个主题或概念，我会向您询问。

Researcher

Are you ready?

您准备好了吗?

Informant 004

Yes.

准备好了。

Researcher

First, some questions about yourself. How old are you now?

首先是关于您个人的一些问题。请问您现在的年龄是多少?

Informant 004

I am 23 years old.

我今年 23 岁。

Researcher

What kind of work are you doing now?

请问您现在从事什么工作呢？

Informant 004

I am an elementary school teacher.

我是一名小学教师。

Researcher

What is the area of your house?

你的房子的面积是多少?

Informant 004

About 105 square meters

大约 105 平方米

Researcher

How many people are in your household? What does the family structure look like?

您的家庭人数？家庭结构是什么样的？

Informant 004

Three people, my parents and me

3 人，父母和我

Researcher

What style of furniture is in the home?

家中家具是什么样式的？

Informant 004

chinese style furniture

中式家具

Researcher

Where is the custom furniture placed? Which cabinets are the main ones?

定制家具放置在哪里？主要是哪些柜体？

Informant 004

In the master and assistant bedrooms, there are wardrobes and TV tables

主副卧室里，有衣柜和电视桌

Researcher

What is your custom furniture style like? Is it consistent with the decoration style of the home?

您家定制家具风格是什么样？和家中装修风格一致吗？

Informant 004

都是中式或者新中式，和家中装修风格不违和

Are Chinese or new Chinese style, and home decoration style is compatible

Researcher

How much do you spend on custom furniture?

你花多少钱在定制家具上?

Informant 004

Thirty or forty thousand yuan.

三四万吧。

Researcher

What is your understanding of custom furniture?

您对定制家具的理解是什么？

Informant 004

For custom furniture, I think it is a home product that is designed and customized according to your specific needs and home environment. Compared with ready-made goods, customized furniture can better meet our individual needs and make our home life more comfortable and beautiful. In addition, custom furniture allows us to have more choices in home style, not limited to ready-made products on the market. We can freely choose materials, colors, sizes, styles, etc. according to our own preferences, so as to create a unique home style.

对于定制家具，我认为它是一种根据自己的具体需求和家庭环境状况来进行设计定制的家居产品。相比于现成的商品，定制家具可以更好地符合我们的个性化需求，让我们的家居生活更加舒适和美好。此外，定制家具还能让我们在家居风格上有更多的选择，不必局限于市场上现成的商品。我们可以根据自己的喜好，自由选择材料、颜色、尺寸、样式等等，从而打造一种独具特色的家居风格。

Researcher

What do you know about the custom furniture brand channel?

您了解定制家具品牌渠道是什么?

Informant 004

TV commercials, introductions from friends

电视广告，朋友介绍

Researcher

How did you learn about custom furniture?

您是怎么了解定制家具相关内容?

Informant 004

The introduction of the receptionist when buying furniture, Internet search

购买家具时接待人员的介绍，网络搜寻

Researcher

What was your initial impression of the brand you chose? What was the initial understanding?

您对您选择的品牌最初印象是什么？最初的理解是什么？

Informant 004

Brand reputation is good, friends around are buying, cost-effective

品牌口碑较好，身边朋友的都在买，性价比较高

Researcher

Why did you choose the brand's bespoke furniture?

您选择该品牌的定制家具的原因是什么？

Informant 004

Provide more satisfactory service and price

提供较为满意的服务与价格

Researcher

So is service important to you?

所以对您来说，服务也很重要吗？

Informant 004

Yes, it is offline explanation, or designer communication, I think these can be counted within the scope of services.

是的，无论是线下的讲解，还是设计师的沟通，我觉得这些都可以算在服务范围内。

Researcher

What about after-sales service?

那售后服务呢？

Informant 004

After-sales service is also very important, can't I buy out, the company doesn't care. If I encounter any problems in the future, I hope the company can still help me solve them.

售后服务也很重要，不能我买完了，公司就不管了吧。我后续如果遇到什么问题，我希望公司还是可以帮助我解决。

Researcher

What do you think are the advantages of custom-made furniture over finished furniture?

您认为相比成品家具，定制家具的优势是什么?

Informant 004

It can be flexible and adjusted according to the needs of different users and different use environment

能够根据不同用户的需求和不同的使用环境进行灵活变通与调节

Researcher

What do you think you should pay attention to when choosing custom furniture? 您觉得在选择定制家具时应该注意什么问题？

Informant 004

Brand, price, workmanship, material, waiting time. I hope the renovation doesn't

drag on too long, and after installing the custom furniture, I have to open the window again to spread the smell.
品牌，价格，做工，材质，等待时间，我希望装修不要拖太久，安装好定制家具后还要再开窗散散味道。

Researcher

How often do you use cabinets, wardrobes, and other custom furniture?
您使用橱柜、衣柜、和其他定制的家具的频率是如何的？

Informant 004

Just like normal furniture
和正常家具无异

Researcher

Does the current custom furniture product look meet your needs?
当前定制家具产品外观满足您的需求吗?

Informant 004

More satisfied
较为满意

Researcher

Do the tactile details of current custom furniture products meet your needs?
当前定制家具产品触觉细节满足您的需求吗?

Informant 004

The more details, the higher the price required, cost-effective is the focus of customer consideration
细节越多，所需的价钱越高，性价比是客户考虑的重点

Researcher

Does the current custom furniture fit your needs for product functionality? Which need is not being met?

当前的定制家具是否符合您对产品功能的需求？哪一个需求没有得到满足？

Informant 004

Most of the needs can be met

大部分需求都能满足

Researcher

Does the current custom furniture fit your needs for product audibility or smell?

当前定制家具是否符合您对产品可听性或气味的需求？

Informant 004

The smell of customized furniture is heavy and it takes more time to volatilize formaldehyde.

定制家具的气味较重，需要较多时间来挥发甲醛。

Researcher

What is the way your custom furniture opens and closes doors? Which way do you prefer to open and close doors?

您家定制家具开关门方式是什么样的？您喜欢哪种开关门方式？

Informant 004

Sliding doors and sliding doors are available. Sliding doors. Sliding doors save space.

拉门和推拉门都有。推拉门，推拉门节省空间。

Researcher

Will you share your renovation success with others?

您会与别人分享您的装修成功经验吗?

Informant 004

Of course, if people ask.

当然，如果别人询问的话。

Researcher

What do you think are the disadvantages of current custom furniture?

您觉得当前的定制家具的缺点是什么?

Informant 004

The disadvantages of current custom furniture are not only that it takes longer to design and make, the price is higher, there are also some other problems. For example, many custom furniture does not have much choice, making it difficult to meet individual needs.

当前定制家具的缺点不仅仅在于设计和制作所需的时间较长，价格较高，还存在一些其他问题。例如，许多定制家具没有太多的选择性，从而难以满足个性化需求。

Researcher

What other features do you think custom furniture can add?

您觉得定制家具可以添加什么其他功能？

Informant 004

Removable and replaceable furniture allows components and accessories to be replaced on demand, enabling rapid renewal and sustainability. In this way, a piece of furniture can be used for many years, otherwise it may be advantageous that the location can still be used, but because the parts are broken, a whole piece of custom furniture has to be thrown away.

可拆卸更换性家具可以按需更换组件和配件，从而实现快速更新和可持续发展。这样就可以使用一件家具很多年，否则可能优点位置还可以使用，但因为部件坏掉了，一整件定制家具都要扔掉。

Researcher

What aspects of custom furniture can provide users with more possibilities?

定制家具的哪些方面可以为用户提供更多的可能性?

Informant 004

Adapt to the user's self-creativity, let the user to create.

适应用户的自我创造性，让用户自己来创造。

Researcher

Thank you for joining us in this interview.

感谢您参与我们的本次访谈。
