# Supplementary material for: Building a 4E interview-grounded theory model: A case study of demand factors for customized furniture
Source: PLoS One. 2023 Apr 27;18(4):e0282956. doi: 10.1371/journal.pone.0282956 (PMC10138260; doi:10.1371/journal.pone.0282956)
Supplement: S1 File — (ZIP) [file pone.0282956.s001.zip › transcript/transcript 005.pdf]

Informant : 005

Please note that the original transcript is in Simplified Chinese. The English translation is for internal communication among the author of this research, and it is not proofread. Potential linguistic errors may exist in the English translation.

Researcher

Thank you for your willingness to participate and be interviewed here. My name is XXX, and I'm a PhD in the XXX University. Currently, I am working on a research project that focuses on collecting information about user demand when purchasing and using customized furniture. Throughout the interview, I will ask you a series of questions and you are encouraged to express your opinions and views freely. During the interview, I will ask you if I have questions about what you have said or if I need you to clarify a topic or concept.

感谢您愿意参加并在此接受采访。我叫 XXX，是 XXX 大学的博士。目前，我正在开展一个研究项目，主要收集在使用定制家具时的用户体验资料。在整个访谈中，我会问您一系列问题，我们鼓励您自由表达您的意见和观点。在访谈过程中，如果我对你所说的内容有疑问或需要您澄清一个主题或概念，我会向您询问。

Researcher

Are you ready?

您准备好了吗?

Informant 005

Yes.

准备好了。

Researcher

First, How old are you now?

请问您现在的年龄是多少?

Informant 005

I am 29 years old.

我今年 29 岁。

Researcher

What kind of work are you doing now?

请问您现在从事什么工作呢？

Informant 005

I am a writer.

我是一名作家。

Researcher

What is the area of your house?

您的房子的面积是多少?

Informant 005

360 square meters

360 平。

Researcher

How many people are in your household? What does the family structure look like?

您的家庭人数？家庭结构是什么样的？

Informant 005

3 people. Me, Dad, Mom.

3 人。我、爹、妈。

Researcher

What style of furniture is in the home?

家中家具是什么样式的？

Informant 005

Chinese style. I like traditional Chinese culture very much, it has a unique charm. Our home is in the traditional culture, adding new fashion and unique taste in it, such Chinese decoration is more suitable for people's daily life. This house is my wedding room and is currently under renovation.

中式风格。我很喜欢中国传统文化，它有一种独特的韵味。我们家是在传统的文化里面，加入了新的时尚和独特的品味在里面，这样的中式装修更贴切人们的日常生活。这个房子是我的婚房，目前还在装修中。

Researcher

Where is the custom furniture placed? Which cabinets are the main ones?

您家的定制家具放置在哪里？主要是哪些柜体？

Informant 005

Door, porch, kitchen, bedroom changing room, balcony. Change the shoe cabinet in the elevator room at the door, change the wardrobe against the wall near the porch, kitchen cabinet, bedroom locker room cabinet, balcony side cabinet.

门口、玄关、厨房、卧室换衣间、阳台。门口电梯间换鞋柜、玄关附近靠墙换衣柜、厨房柜子、卧室换衣间柜子、阳台侧面柜子。

Researcher

What is your custom furniture style like? Is it consistent with the decoration style of the home?

Informant 005

您家定制家具风格是什么样？和家中装修风格一致吗？

The cabinet in the elevator room and the wardrobe near the entrance are more Chinese; The kitchen cabinets are more modern, the bedroom cabinets are more Chinese, and the balcony side cabinets are more modern. Not too consistent, but not too conflicting in style

电梯间柜子、玄关附近靠墙换衣柜偏中式；厨房柜子偏现代、卧室柜子偏中式一点、阳台侧面柜子偏现代。不太一致，但风格不会太冲突

Researcher

How much do you spend on custom furniture?

你花多少钱在定制家具上?

Informant 005

More than 200,000 yuan

20 多万元

Researcher

What is your understanding of custom furniture?

您对定制家具的理解是什么？

Informant 005

Furniture that is fully based on a specific space and can meet customers' personalized needs for furniture function, material, and appearance, and improve the quality of life. The designer will listen carefully to our needs and ideas, make meticulous design, and take into account the actual situation of our family in the actual production to ensure that the customized furniture can be perfectly integrated into our family. Moreover, because custom furniture belongs to personalized products, its quality and materials will be more advanced and more durable. In short, in addition to meeting our individual needs, customized furniture

can also make our home life more comfortable, beautiful, personalized and high-quality.
充分依据特定的空间打造的、可以满足顾客对于家具功能、材质、外观的个性化需求、提高生活品质的家具。设计师会仔细聆听我们的需求和想法，进行细致的设计，并在实际制作中考虑到我们家庭的实际情况，确保定制的家具能够完美地融入我们的家庭之中。而且，由于定制家具属于个性化定制产品，它的质量和材料都会更高级，更加耐用。总之，定制家具除了可以满足我们个性化的需求，还能让我们的家居生活更加舒适、美好、个性化和高质量。

Researcher

What do you know about the custom furniture brand channel?

您了解定制家具品牌渠道是什么?

Informant 005

Advertising, word of mouth, friend recommendation

广告、口碑、朋友推荐

Researcher

How did you learn about custom furniture?

您是怎么了解定制家具相关内容?

Informant 005

One is online, mainly through online channels such as e-commerce platforms and social media; The following is offline, offline is mainly through exhibitions, shopping malls, specialty stores and other physical channels to understand.
一个是线上，主要是通过电商平台、社交媒体等网络渠道了解；一下是线下，线下主要是通过展览、商场、专卖店等实体渠道选购的时候进行了解。

Researcher

What was your initial impression of the brand you chose? What was the initial understanding?

您对您选择的品牌最初印象是什么？最初的理解是什么？

Informant 005

It looks professional. Well-known brand, material safety and environmental protection

看起来比较专业。知名度高的品牌、材料安全环保

Researcher

Why did you choose the brand's bespoke furniture?

您选择该品牌的定制家具的原因是什么？

Informant 005

When I choose furniture, the safety, environmental friendliness and quality of the materials are one of the three important aspects I consider. The material is safe and environmentally friendly, and there will be no pollutants such as formaldehyde, which have adverse effects on people's health and the environment.

我选择家具时，材质的安全性、环保性和品质是我考虑的三个重要方面之一。材料安全环保，不会有甲醛等污染物，这些物质对人们的健康和环境有不良影响。

Researcher

What do you think are the advantages of custom-made furniture over finished furniture?

您认为相比成品家具，定制家具的优势是什么?

Informant 005

Unified design according to the space, the appearance has integrity. Custom-designed according to the items I expect to place, to meet the needs of use,

appearance, etc. Custom furniture was able to provide the perfect furniture solution according to my space needs and dimensions. Through the comprehensive unified design of the space, my room can be more harmonious and unified, visually present a more high-end effect, and maximize the use of space.
根据空间统一设计，外观有整体性。根据我期望放置的物品进行定制设计，满足使用需求、外观需求等。定制家具能够根据我的空间需求和尺寸，提供最完美的家具解决方案。通过对空间进行全面的统一设计，能使我房间更加和谐统一，在视觉上呈现出更加高端的效果，让空间得到最大化的利用。

Researcher
What do you think you should pay attention to when choosing custom furniture?
您觉得在选择定制家具时应该注意什么问题？

Informant 005
When choosing custom furniture, I will consider many aspects, such as the manufacturer's reputation, after-sales service, cost and so on. Therefore, before choosing custom furniture, I also carefully look at the company's behind-the-scenes capabilities to ensure that I can get the most satisfying custom furniture. In addition, it is also important to communicate with the designer and express your own ideas, and it is necessary to properly refer to the designer's opinion.
在选择定制家具时，我会考虑到诸多方面，比如厂家的口碑、售后服务、成本等等。因此，在选择定制家具前，我还会仔细了解公司的幕后实力，以确保能获得最令人满意的定制家具。此外，跟设计师沟通、表达自己的想法，也是很重要的，要适当参考设计师的意见。

Researcher
How often do you use cabinets, wardrobes, and other custom furniture?
您使用橱柜、衣柜、和其他定制的家具的频率是如何的？

Informant 005

High frequency of kitchen cabinet, wardrobe and shoe cabinet, low frequency of balcony cabinet.

橱柜、衣柜、鞋柜使用频率高、阳台柜使用频率低。

Researcher

Does the current custom furniture product look meet your needs?

当前定制家具产品外观满足您的需求吗?

Informant 005

Satisfied.

满足。

Researcher

Does the current custom furniture fit your needs for product functionality? Which need is not being met?

当前的定制家具是否符合您对产品功能的需求？哪一个需求没有得到满足？

Informant 005

Basically meets my needs

基本都符合我的需求。

Researcher

What is the way your custom furniture opens and closes doors? Which way do you prefer to open and close doors?

您家定制家具开关门方式是什么样的？您喜欢哪种开关门方式？

Informant 005

It depends on the type of furniture. Choose sliding doors for changing wardrobe. The

kitchen has sliding doors and sliding doors. The bedroom cabinet has sliding doors and sliding doors.
取决于家具种类。换衣柜选择滑动门，厨房有开关门、推拉门，卧室柜子是推拉门、开关门。

Researcher
Will you share your renovation success with others?
您会与别人分享您的装修成功经验吗?

Informant 005
不会。
Nope.

Researcher
Why not share it?
为什么不会分享呢？

Informant 005
If someone hadn't asked, I wouldn't have offered to share my renovation experience with others.
如果不是有人来询问的话，我不会主动向他人分享装修经验。

Researcher
What do you think are the disadvantages of current custom furniture?
您觉得当前的定制家具的缺点是什么?

Informant 005
In the current custom furniture market, although consumers can customize according to their own needs and preferences, it is relatively simple in appearance. There are

often only a few design types, and there is not much innovation and variation, making people feel less fresh and personalized when choosing. In addition, due to the difference in accessories and other aspects, customized furniture often has the problem of later maintenance and replacement of accessories, and these will also increase the use of trouble and cost for consumers.

在当前的定制家具市场中，尽管消费者可以按照自己的需求和喜好进行订制，但是在外观上却相对比较单一。往往只有少数设计种类，而且没有太多创新和变化，使得人们选择时感觉缺乏新鲜感和个性化。另外，由于配件等方面的不同，定制家具常常存在后期维修与更换配件的问题，而这些也会增加消费者的使用麻烦及成本。

Researcher

What aspects of custom furniture can provide users with more possibilities?

定制家具的哪些方面可以为用户提供更多的可能性?

Informant 005

The traditional way of opening the door may require pulling cabinet handles or sliding doors, which is relatively inconvenient and unhygienic. Touchless electronic switches or inductive switches are now available to easily open or close furniture with a flick of a hand or close to the door. The advantages of this switch method are convenient, fast, hygienic and clean, and it is also very suitable for families with elderly or children at home.

传统的开门方式可能需要拉柜把手或者推拉门，用起来相对不太方便，也不太卫生。而现在可以采用无触控电子开关或者感应式开关，只要手轻轻一触或者靠近门，就能轻松开启或关闭家具。这种开关方式的优势是方便快捷、卫生清洁，也非常适合家中有老人或者小孩的家庭。

Researcher

Thank you for participating in this interview and have a great life.

感谢您参与本次的访谈，祝您生活愉快。
