# Supplementary material for: Building a 4E interview-grounded theory model: A case study of demand factors for customized furniture
Source: PLoS One. 2023 Apr 27;18(4):e0282956. doi: 10.1371/journal.pone.0282956 (PMC10138260; doi:10.1371/journal.pone.0282956)
Supplement: S1 File — (ZIP) [file pone.0282956.s001.zip › transcript/transcript 006.pdf]

Informant : 006

Please note that the original transcript is in Simplified Chinese. The English translation is for internal communication among the author of this research, and it is not proofread. Potential linguistic errors may exist in the English translation.

Researcher

Thank you for your willingness to participate and be interviewed here. My name is XXX, and I'm a PhD in the XXX University. Currently, I am working on a research project that focuses on collecting information about user demand when purchasing and using customized furniture. Throughout the interview, I will ask you a series of questions and you are encouraged to express your opinions and views freely. During the interview, I will ask you if I have questions about what you have said or if I need you to clarify a topic or concept.

感谢您愿意参加并在此接受采访。我叫 XXX，是 XXX 大学的博士。目前，我正在开展一个研究项目，主要收集在使用定制家具时的用户体验资料。在整个访谈中，我会问您一系列问题，我们鼓励您自由表达您的意见和观点。在访谈过程中，如果我对你所说的内容有疑问或需要您澄清一个主题或概念，我会向您询问。

Researcher

Are you ready?

您准备好了吗?

Informant 006

Yes.

准备好了。

Researcher

How old are you now?

请问您现在的年龄是多少?

Informant 006

I am 33 years old.

我今年 33 岁。

Researcher

What kind of work are you doing now?

请问您现在从事什么工作呢？

Informant 006

I am a IT.

我是一名 IT.

Researcher

What is the area of your house?

你的房子的面积是多少?

Informant 006

120 square meters

120 平米

Researcher

How many people are in your household? What does the family structure look like?

您的家庭人数？家庭结构是什么样的？

Informant 006

Three people, me, wife and a child

三人，我，妻子和小朋友

Researcher

What style of furniture is in the home?

家中家具是什么样式的？

Informant 006

Wooden furniture

木制家具

Researcher

Is it solid wood furniture?

是实木家具吗？

Informant 006

Yes, the home is dominated by oak and walnut furniture.

是的，家中的主要是橡木和胡桃木家具。

Researcher

Where is the custom furniture placed?

定制家具放置在哪里?

Informant 006

Custom furniture is mainly placed in the living room and bedroom, mainly wardrobes, cabinets, bookcases and bedside tables.

定制家具主要是放在客厅和卧室，主要是衣柜、橱柜、书柜和床头柜。

Researcher

What is your custom furniture style like? Is it consistent with the decoration style of the home?

您家定制家具风格是什么样？和家中装修风格一致吗？

Informant 006

The style is all wooden vintage, which is consistent with the overall style of the home.
风格都是木制复古，和家中整体的风格一致。

Researcher

How much money do you spend on custom furniture?
你们花了多少钱在定制家具上?

Informant 006

My wife spends money, I donot know
妻子花钱，我自己不知晓。

Researcher

So the power of your family's finances is in the hands of your wife?
那您家的财政的大权是掌握在妻子手中啊？

Informant 006

Haha yes, I'm only responsible for handing over the salary.
哈哈是的，我只负责将工资上缴就可以。

Researcher

What is your understanding of custom furniture?
您对定制家具的理解是什么？

Informant 006

Custom furniture refers to the designer according to the needs and requirements of the owner, design and manufacture furniture that meets the owner's requirements. I can customize the size, style, and function according to your own preferences to meet our personalized needs in space design, color matching, size and function.

定制家具是指设计师根据业主的需求和要求，设计并制造符合业主要求的家具。可以根据自己喜好来定制大小，样式，功能，满足我们在空间设计、色彩搭配、尺寸和功能等方面的个性化需求。

Researcher

What do you know about the custom furniture brand channel?

您了解定制家具品牌渠道是什么?

Informant 006

advertisement

广告

Researcher

How did you learn about custom furniture?

您是怎么了解定制家具相关内容?

Informant 006

Search on the Internet, and you will also go to the local building materials market to learn about the condition of customized furniture.

上网搜索，也会去当地的建材市场转悠转悠了解定制家具状况。

Researcher

Is the Internet search mainly a WeChat public account or a Baidu search?

那上网搜索的话主要是微信公众号、还是百度搜索？

Informant 006

Will take a look, will also go to the video website to find out.

都会看看，也会去视频网站了解一下。

Researcher

What was your initial impression of the brand you chose? What was the initial understanding?

您对您选择的品牌最初印象是什么？最初的理解是什么？

Informant 006

The exterior styling meets the requirements, which is both beautiful and comfortable. It can be designed according to our needs, both to save space and to choose furniture according to our preferences, so that we can create a comfortable home.

外观造型符合要求，既美观又舒适。可以根据我们的需求进行设计，既可以节省空间，也可以根据自己的喜好来选择家具，这样就能创造出一个舒适的家。

Researcher

Why did you choose the brand's bespoke furniture?

您选择该品牌的定制家具的原因是什么？

Informant 006

We mainly consider the brand awareness we choose, so that at least it will not be a three-nil product, and the quality is guaranteed. Secondly, this brand after-sales service will be good, and you can find someone to deal with problems if you have problems.

我们主要是考虑我们选择的品牌知名度，这样至少不会是三无产品，质量有保障。其次这种品牌售后服务做的也会还不错，有问题可以找得到人进行处理。

Researcher

What do you think are the advantages of custom-made furniture over finished furniture?

您认为相比成品家具，定制家具的优势是什么?

Informant 006

The custom furniture fully considers the space layout of the house in the design, cleverly uses every inch of space, and can accommodate more people without increasing the area of the house. And the design of customized furniture is closer to people's lives, and its materials, colors, styles, functions, etc. can be designed and selected according to the needs of our family.
定制家具在设计中充分考虑了房屋的空间布局，巧妙的利用每一寸空间，在不增加房屋面积的情况下，可以容纳更多人使用。而且定制家具在设计上更加贴近人们的生活，其材质、色彩、款式、功能等方面都可以根据我们一家人的需求来进行设计和选择。

Researcher
What do you think you should pay attention to when choosing custom furniture?
您觉得在选择定制家具时应该注意什么问题？

Informant 006
I don't think I know much about the industry, so I don't know a lot of questions. The first is to confirm the material with the merchant when choosing custom furniture. Because many custom furniture manufacturers are now small manufacturers, they will cut corners in the production and production process, in order to make custom furniture more durable, they use more on the board. Therefore, when choosing furniture, we must determine the material with the merchant, and do not wait until there is a problem in the later stage to find the merchant.
我觉得自己对这个行业不是很了解，所以很多问题都不清楚。首先就是在挑选定制家具的时候一定要和商家确认清楚材质。因为现在很多定制家具厂家都是小厂家，在生产和制作过程中会偷工减料，为了让定制家具更耐用，他们在板材上的使用量就比较多。所以我们在选择家具的时候一定要和商家确定清楚材质，不要等到后期出现问题了才来找商家。

Researcher
How often do you use cabinets, wardrobes, and other custom furniture?

您使用橱柜、衣柜、和其他定制的家具的频率是如何的？

Informant 006

Often

经常

Researcher

Does the current custom furniture fit your needs for product functionality? Which need is not being met?

当前的定制家具是否符合您对产品功能的需求？哪一个需求没有得到满足？

Informant 006

Basically met our needs.

基本都满足我们的需求。

Researcher

What is the way your custom furniture opens and closes doors? Which way do you prefer to open and close doors?

您家定制家具开关门方式是什么样的？您喜欢哪种开关门方式？

Informant 006

I prefer sliding doors, which don't take up space.

我比较喜欢推拉门，不占空间。

Researcher

Will you share your renovation success with others?

您会与别人分享您的装修成功经验吗?

Informant 006

Yes

会

Researcher

What do you think are the disadvantages of current custom furniture?

您觉得当前的定制家具的缺点是什么?

Informant 006

Some custom furniture materials are not environmentally friendly and have certain hazards to human health. In addition, the price of custom furniture is higher than other furniture because the materials are limited. Even if you buy custom furniture from a big brand, if the material is not good, it is not worth buying it. That is, custom furniture does not have a unified specification to use, so there will be many problems in decoration. For example, the price is still not too cheap, so you also need to consider the price when customizing furniture, after all, the price problem is also the most important.

有些定制家具材料不环保，对人体健康有一定的危害。此外，定制家具的价格比其他家具高，因为材料有限。即使你买了一个大品牌的定制家具，如果材料不好，也不值得买它。就是定制家具没有统一的规格来使用，所以在装修上就会存在很多问题。比如价格方面还是不算太便宜，所以在定制家具的时候还需要考虑一下价格方面，毕竟价格上的问题也是最重要的。

Researcher

What other features do you think custom furniture can add?

您觉得定制家具可以添加什么其他功能？

Informant 006

Replaceable accessory function. You can consider adding drawers, baskets, clothes rails, increase storage space, or you can add shelves, clothes rails and other accessories, when making multi-functional cabinets, you can also add small drawers or small clothes rails to meet different needs; Also, it would be nice if adjustable

shelves could be added. Adjust the height of the shelf according to actual needs, so that the wardrobe space is more practical and flexible.

可替换配件功能。可以考虑添加抽屉、拉篮、衣杆，增加储物空间，或者可以添加层板、挂衣杆等配件，在做多功能柜体的时候，还可以添加小抽屉或者小挂衣杆，满足不同需求；此外如果可以增加可调节搁板，那就太好了。根据实际需求去调整层板的高度，让衣柜空间更加实用和灵活。

Researcher

What aspects of custom furniture can provide users with more possibilities?

定制家具的哪些方面可以为用户提供更多的可能性?

Informant 006

After-sales service. If something is broken, the replacement of accessories can be carried out.

售后服务吧。东西坏了可以进行配件的更换。

Researcher

Okay, thank you for receiving our interview

好的，谢谢您接收我们的访谈
