# Supplementary material for: Building a 4E interview-grounded theory model: A case study of demand factors for customized furniture
Source: PLoS One. 2023 Apr 27;18(4):e0282956. doi: 10.1371/journal.pone.0282956 (PMC10138260; doi:10.1371/journal.pone.0282956)
Supplement: S1 File — (ZIP) [file pone.0282956.s001.zip › transcript/transcript 007.pdf]

Informant : 007

Please note that the original transcript is in Simplified Chinese. The English translation is for internal communication among the author of this research, and it is not proofread. Potential linguistic errors may exist in the English translation.

Researcher

Thank you for your willingness to participate and be interviewed here. My name is XXX, and I'm a PhD in the XXX University. Currently, I am working on a research project that focuses on collecting information about user demand when purchasing and using customized furniture. Throughout the interview, I will ask you a series of questions and you are encouraged to express your opinions and views freely. During the interview, I will ask you if I have questions about what you have said or if I need you to clarify a topic or concept.

感谢您愿意参加并在此接受采访。我叫 XXX，是 XXX 大学的博士。目前，我正在开展一个研究项目，主要收集在使用定制家具时的用户体验资料。在整个访谈中，我会问您一系列问题，我们鼓励您自由表达您的意见和观点。在访谈过程中，如果我对你所说的内容有疑问或需要您澄清一个主题或概念，我会向您询问。

Researcher

Are you ready?

您准备好了吗?

Informant 007

Yes.

准备好了。

Researcher

How old are you now?

请问您现在的年龄是多少？

Informant 007

I am 34 years old.

我今年 34 岁。

Researcher

What kind of work are you doing now?

请问您现在从事什么工作呢？

Informant 007

I am a salesperson.

我是一名销售员。

Researcher

What is the area of your house?

你的房子的面积是多少?

Informant 007

109 square

109 平。

Researcher

How many people are in your household?

您的家庭人数？

Informant 007

A family of four. three rooms, two living rooms, one kitchen, two bathrooms and a balcony

一家四口，房子是三房两厅一厨两卫一阳台

Researcher

What style of furniture is in the home?

家中家具是什么样式的？

Informant 007

Chinese recliner Western cowhide sofa mix and match

中式躺椅西式牛皮沙发混搭

Researcher

Where is the custom furniture placed?

您家的定制家具放置在哪里?

Informant 007

The custom furniture in our home is mainly the wardrobe and desk in the room, and the rest of the custom cabinets play a role in the storage hallway in the living room and dining room.

我们家的定制家具主要是房间内的衣柜和书桌，其余定制柜体在客厅餐厅起到储物玄关等作用。

Researcher

What is your custom furniture style like? Is it consistent with the decoration style of the home?

您家定制家具风格是什么样？和家中装修风格一致吗？

Informant 007

Wood Chinese style, the style is roughly the same

木材中式风，风格大体一致

Researcher

How much do you spend on custom furniture?

你花多少钱在定制家具上?

Informant 007

50,000 to 60,000, on a case-by-case basis

5 万到 6 万，具体问题具体分析

Researcher

What is your understanding of custom furniture?

您对定制家具的理解是什么？

Informant 007

Create the highest quality furniture that matches the room type. Tailor-made furniture, customized furniture according to consumer needs, which is also one of the biggest features of custom furniture. Finished furniture is mainly produced and sold by brand enterprises, and customized furniture is designed and manufactured by individual designers or corporate designers according to consumer needs. And custom homes require factory mass production.

打造符合房型的最优质家具。量身定做的家具，根据消费者需求来定制家具，这也是定制家具最大的特点之一。成品家具主要由品牌企业生产销售，定制家具是由个人设计师或企业设计师根据消费者需求设计制造而成。而且定制家居需要工厂批量生产。

Researcher

What do you know about the custom furniture brand channel?

您了解定制家具品牌渠道是什么?

Informant 007

It started as a TV variety show - Dream Makeover

最开始是电视综艺节目——梦想改造家

Researcher

How did you learn about custom furniture?

您是怎么了解定制家具相关内容?

Informant 007

咨询专业人士，上网调查

Consult professionals and research online

Researcher

What was your initial impression of the brand you chose?

您对您选择的品牌最初印象是什么?

Informant 007

The initial impression is in line with personal aesthetics, and it is a more professional brand. The plate materials are all E1 grade materials, which are safe and healthy.

最初印象符合个人审美， 是个较为专业的品牌。板材材质都是 E1 级别的材料，安全健康。

Researcher

Why did you choose the brand's bespoke furniture?

您选择该品牌的定制家具的原因是什么？

Informant 007

Designers are cooperating with custom furniture manufacturers, basically first out of the renderings, and then according to the renderings to design, the designer will give a series of product layout schemes, such as drawers, door panels, cabinet doors, etc., and then let the customer choose. It is believed that its design style uses materials to meet the needs of custom furniture, and the price is also more appropriate.

设计师都是与定制家具生产厂家合作的，基本都是先出效果图，然后根据效果图来设计，设计师会给出一系列的产品布局方案，如抽屉、门板、柜门等等，然后让客户进行选择。认为其设计风格使用材质符合对定制家具的需求，而且价格也比较合适。

Researcher

What do you think are the advantages of custom-made furniture over finished furniture?

您认为相比成品家具，定制家具的优势是什么?

Informant 007

For us, the advantages of custom furniture are mainly reflected in the two aspects of design and price. In terms of product design, custom furniture has flexibility and diversity, can be designed according to the needs of consumers, providing consumers with more choices, and more possibilities in material selection. In terms of price, custom furniture is an overall planning of the function, style and style of furniture within a certain price range, and custom furniture is more cost-effective than finished furniture.

对于我们来说，定制家具的优势主要体现在设计和价格两个方面。在产品设计上，定制家具具有灵活性和多样性，可以根据消费者的需求进行设计，为消费者提供了更多选择，而且在材质选择上有更多可能性。就价格而言，定制家具是在一定价格范围内对家具的功能、款式和风格进行整体规划，相比成品家具来说，定制家具的性价比更高。

Researcher

What do you think you should pay attention to when choosing custom furniture?

您觉得在选择定制家具时应该注意什么问题？

Informant 007

Whether the material is safe and durable, and whether the customized size is

accurate. If there is a deviation in the dimensions, it will lead to a space that does not fit into the house.

材质是否安全耐用，定制大小是否精确。如果尺寸上有偏差，就会导致不适合房屋的空间。

Researcher

How often do you use cabinets, wardrobes, and other custom furniture?

您使用橱柜、衣柜、和其他定制的家具的频率是如何的？

Informant 007

The frequency is very high

频率十分高

Researcher

Does the current custom furniture product look meet your needs?

当前定制家具产品外观满足您的需求吗?

Informant 007

Relatively satisfied.

较为满足。

Researcher

Does the current custom furniture fit your needs for product functionality? Which need is not being met?

当前的定制家具是否符合您对产品功能的需求？哪一个需求没有得到满足？

Informant 007

Yes, some quality problems occur with the increase of use time.

满足，质量随着使用时间增加出现部分问题。

Researcher

For example?

举个例子呢？

Informant 007

That is, the hardware will be broken, which directly leads to the door not closing tightly or even falling directly.

就是五金会坏掉，这就直接导致门会出现关不紧甚至是直接掉下来的状况。

Researcher

What is the way your custom furniture opens and closes doors? Which way do you prefer to open and close doors?

您家定制家具开关门方式是什么样的？您喜欢哪种开关门方式？

Informant 007

Push and pull, no very favorite way

前后推拉，没有非常喜欢的方式

Researcher

Will you share your renovation success with others?

您会与别人分享您的装修成功经验吗?

Informant 007

YES

会

Researcher

What do you think are the disadvantages of current custom furniture?

您觉得当前的定制家具的缺点是什么?

Informant 007

Accessories are inconvenient to replace

配件更换不方便

Researcher

What other features do you think custom furniture can add?

您觉得定制家具可以添加什么其他功能？

Informant 007

Accessories can be replaced flexibly

配件可以灵活更换

Researcher

What aspects of custom furniture do you think can provide more possibilities for users?

您觉得定制家具的哪些方面可以为用户提供更多的可能性?

Informant 007

Material selection and flexible replacement style for local design. With the progress of society, people's requirements for home life are getting higher and higher, and traditional custom furniture can no longer meet people's needs, and the designer's secondary creation is required in some details.

局部设计的材质选择与灵活更换风格。随着社会的进步，人们对家居生活要求越来越高，传统的定制家具已经无法满足人们的需求，在一些细节上就需要设计师的二次创作。

Researcher

Okay, this interview is about this technology. Wish you a happy life.

好的，本次的访谈到此技术，祝您生活愉快。
