# Supplementary material for: Building a 4E interview-grounded theory model: A case study of demand factors for customized furniture
Source: PLoS One. 2023 Apr 27;18(4):e0282956. doi: 10.1371/journal.pone.0282956 (PMC10138260; doi:10.1371/journal.pone.0282956)
Supplement: S1 File — (ZIP) [file pone.0282956.s001.zip › transcript/transcript 008.pdf]

Informant : 008

Please note that the original transcript is in Simplified Chinese. The English translation is for internal communication among the author of this research, and it is not proofread. Potential linguistic errors may exist in the English translation.

Researcher

Thank you for your willingness to participate and be interviewed here. My name is XXX, and I'm a PhD in the XXX University. Currently, I am working on a research project that focuses on collecting information about user demand when purchasing and using customized furniture. Throughout the interview, I will ask you a series of questions and you are encouraged to express your opinions and views freely. During the interview, I will ask you if I have questions about what you have said or if I need you to clarify a topic or concept.

感谢您愿意参加并在此接受采访。我叫 XXX，是 XXX 大学的博士。目前，我正在开展一个研究项目，主要收集在使用定制家具时的用户体验资料。在整个访谈中，我会问您一系列问题，我们鼓励您自由表达您的意见和观点。在访谈过程中，如果我对你所说的内容有疑问或需要您澄清一个主题或概念，我会向您询问。

Researcher

Are you ready?

您准备好了吗?

Informant 008

Yes.

准备好了。

Researcher

First, some questions about yourself. How old are you now?

首先是关于您个人的一些问题。请问您现在的年龄是多少?

Informant 008

I am 26 years old.

我今年 26 岁。

Researcher

What kind of work are you doing now?

请问您现在从事什么工作呢？

Informant 008

I am a teacher.

我是一名老师。

Researcher

What is the size of the house you are currently living in?

你的房子的面积是多少?

Informant 008

120 square meters.

120 平方米。

Researcher

What is your family structure like?

您的家庭结构是什么样的？

Informant 008

Three people, our husband and wife and daughter.

三个人，我们夫妻俩和女儿。

Researcher

What style of furniture is in the home?

家中家具是什么样式的？

Informant 008

Solid wood, modern style.

实木为主，现代风格。

Researcher

Where is the custom furniture placed? Which cabinets are the main ones?

定制家具放置在哪里？主要是哪些柜体？

Informant 008

The entrance, bedroom and kitchen are all placed with custom furniture, such as custom wardrobes, shoe cabinets, lockers, cabinets.

玄关，卧室和厨房都放置了定制家具，比如定制衣柜，鞋柜，储物柜，橱柜。

Researcher

What shape is your cabinet?

您家的橱柜是什么形状的呢？

Informant 008

Our kitchen has little space, and the cabinets are L-shaped.

我们家厨房空间不大，橱柜做的是 L 型。

Researcher

What is your custom furniture style like? Is it consistent with the decoration style of the home?

您家定制家具风格是什么样？和家中装修风格一致吗？

Informant 008

Modern style, consistent..

现代风格，一致。

Researcher

How much do you spend on custom furniture?

你花多少钱在定制家具上?

Informant 008

About 50 thousand yuan.

5 万左右吧

Researcher

What is your understanding of custom furniture?

您对定制家具的理解是什么？

Informant 008

It is more in line with the structure of the house, and exclusive products can be customized according to preferences and personal habits. Customized furniture can provide users with more possibilities, including design, size, color, material, function, etc. Users can choose the right style and configuration according to their needs and preferences, so that the furniture and home style are more in harmony. For example, and hangers can be customized according to the type of clothes.

更加符合房屋结构，可以根据喜好和个人习惯定制专属产品。定制家具可以为用户提供更多的可能性，包括外观设计、尺寸、颜色、材质、功能等方面。用户可以根据自己的需求和喜好，选择合适的款式和配置，让家具与家居风格更加协调一致。例如，挂衣架可以根据衣服类型选择定制。

Researcher

What do you know about the custom furniture brand channel?

您了解定制家具品牌渠道是什么?

Informant 008

Advertising, physical stores.

广告，实体店。

Researcher

Do you visit physical stores when you're ready to buy custom furniture?

您是在准备购买定制家具的时候会去逛实体店吗？

Informant 008

It will be earlier, when buying a house and just starting to decorate hard, I will visit the physical store to understand the condition of soft decoration.

会更早一些，在买了房子刚开始硬装的时候就会逛逛实体店，了解一下软装的状况。

Researcher

So will you spend more on hard or soft furnishings?

那您的钱是花费在硬装上多一些还是软装会多一些呢？

Informant 008

Soft furnishing it. The materials of the furniture selected are all good, afraid of any bad effects on children, so try to choose some good furniture.

软装吧。选择的家具的材料都是还不错的，怕对小孩子产生什么不好的影响，所以都尽量挑选一些好的家具。

Researcher

How did you learn about custom furniture?

您是怎么了解定制家具相关内容?

Informant 008

Network understanding

网络了解

Researcher

Can you explain it in detail?

可以详细说一下吗?

Informant 008

It is WeChat that follows similar public accounts, and then Tiktok video will also pay attention to it.

就是微信关注差不多的公众号，然后抖音视频也会都关注一下。

Researcher

What was your initial impression of the brand you chose? What was the initial understanding?

您对您选择的品牌最初印象是什么？最初的理解是什么？

Informant 008

Initially learned that this brand was recommended by a friend, he said that the furniture of this house is of good quality, healthy and safe. We all have small children, so the issue of health is a very important point.

最初了解到这个牌子就是朋友推荐，他表示这家的家具质量好，健康安全。我们都是有小孩子的，所以健康的问题是一个很重要的点。

Researcher

Why did you choose the brand's bespoke furniture?

您选择该品牌的定制家具的原因是什么？

Informant 008

In the end, the main reason for choosing his home is that the price is reasonable and cost-effective.
最终选择他家的主要就是价格合理，性价比高。

Researcher
What do you think are the advantages of custom-made furniture over finished furniture?
您认为相比成品家具，定制家具的优势是什么?

Informant 008
It is to design furniture styles according to the size of the house and the preferences of the owner, combined with the indoor space layout and the individual needs of the owner, and configure the corresponding furniture hardware accessories.
It not only meets the owner's pursuit of individuality and diversity, but also greatly meets the needs of the furniture market. It is on the basis of traditional furniture, according to personal preferences and requirements, make full use of every inch of space, design and tailor-made according to needs, consumer-centric, to meet the personalized needs of consumers in terms of function, style, style, material and other aspects.
就是根据房屋面积大小和业主喜好要求，结合室内空间布局及业主个性化需求，量身设计家具款式，并配置相应家具五金配件。既满足了业主对个性化、多样性的追求，又极大地满足了家具市场的需求。它是在传统家具的基础上，根据个人爱好和要求，充分利用每一寸空间，按需设计和量身定做，以消费者为中心，满足消费者在使用功能、款式、风格、材质等方面的个性化需求。

Researcher
What do you think you should pay attention to when choosing custom furniture?
您觉得在选择定制家具时应该注意什么问题？

Informant 008

Size issues, coordination with other furniture.

尺寸问题，和其他家具的协调。

Researcher

How often do you use cabinets, wardrobes, and other custom furniture?

您使用橱柜、衣柜、和其他定制的家具的频率是如何的？

Informant 008

Daily use frequency.

日常使用频率。

Researcher

Does the current custom furniture product look meet your needs?

当前定制家具产品外观满足您的需求吗?

Informant 008

Basically satisfied

基本满足

Researcher

Do the tactile details of current custom furniture products meet your needs?

当前定制家具产品触觉细节满足您的需求吗?

Informant 008

I like the custom furniture brand we chose because they do a lot of detail in their home, and the handle is very comfortable to touch.

我看中我们家选择的定制家具品牌就是因为他们家的细节处理做的很多，拉手那儿触摸起来很舒服。

Researcher

Does the current custom furniture fit your needs for product functionality? Which need is not being met?
当前的定制家具是否符合您对产品功能的需求？哪一个需求没有得到满足？

Informant 008
Basically compliant
基本符合

Researcher
What is the way your custom furniture opens and closes doors?
您家定制家具开关门方式是什么样的？

Informant 008
Normal opening and closing door and drawer design. The drawer design of the kitchen cabinet is still relatively comfortable, and you can take out the dishes or bowls in the drawer by pulling and pulling.
普通开合门和抽屉设计。厨房的地柜的抽屉设计还是比较舒适的，通过抽拉的方式就可以拿出抽屉内的盘或碗。

Researcher
Will you share your renovation success with others?
您会与别人分享您的装修成功经验吗?

Informant 008
yes.
会

Researcher
What do you think are the disadvantages of current custom furniture?

您觉得当前的定制家具的缺点是什么?

Informant 008

There are not enough choices, the price is expensive.

选择还不够多，价格贵。

Researcher

What other features do you think custom furniture can add?

您觉得定制家具可以添加什么其他功能？

Informant 008

It can easily change the color and the size of the compartment in the cabinet.

可以很方便的改色、改变柜内隔层大小。

Researcher

What aspects of custom furniture can provide users with more possibilities?

定制家具的哪些方面可以为用户提供更多的可能性?

Informant 008

You can make adjustments according to your preferences.

可以根据自己的喜好做调整。

Researcher

Okay, thank you for receiving our interview

好的，谢谢您接收我们的访谈
