# Supplementary material for: Building a 4E interview-grounded theory model: A case study of demand factors for customized furniture
Source: PLoS One. 2023 Apr 27;18(4):e0282956. doi: 10.1371/journal.pone.0282956 (PMC10138260; doi:10.1371/journal.pone.0282956)
Supplement: S1 File — (ZIP) [file pone.0282956.s001.zip › transcript/transcript 009.pdf]

Informant : 009

Please note that the original transcript is in Simplified Chinese. The English translation is for internal communication among the author of this research, and it is not proofread. Potential linguistic errors may exist in the English translation.

Researcher

Thank you for your willingness to participate and be interviewed here. My name is XXX, and I'm a PhD in the XXX University. Currently, I am working on a research project that focuses on collecting information about user demand when purchasing and using customized furniture. Throughout the interview, I will ask you a series of questions and you are encouraged to express your opinions and views freely. During the interview, I will ask you if I have questions about what you have said or if I need you to clarify a topic or concept.

感谢您愿意参加并在此接受采访。我叫 XXX，是 XXX 大学的博士。目前，我正在开展一个研究项目，主要收集在使用定制家具时的用户体验资料。在整个访谈中，我会问您一系列问题，我们鼓励您自由表达您的意见和观点。在访谈过程中，如果我对你所说的内容有疑问或需要您澄清一个主题或概念，我会向您询问。

Researcher

Are you ready?

您准备好了吗?

Informant 009

Yes.

准备好了。

Researcher

First, some questions about yourself. How old are you now?

首先是关于您个人的一些问题。请问您现在的年龄是多少?

Informant 009

I am 31 years old.

我今年 31 岁。

Researcher

What kind of work are you doing now?

请问您现在从事什么工作呢？

Informant 009

I am a company manager.

我是一个公司经理。

Researcher

What is the area of your house?

您的房子的面积是多少?

Informant 009

600 square

600 平方

Researcher

How many people are in your household? What does the family structure look like?

您的家庭人数？家庭结构是什么样的？

Informant 009

5 people, dad, mom, me, wife and my two-year-old son.

5 人，爸爸、妈妈、我、妻子和我两岁的儿子。

Researcher

What style of furniture is in the home?

家中家具是什么样式的？

Informant 009

Chinese furniture and new Chinese furniture

中式家具和新中式家具

Researcher

Where is the custom furniture placed?

定制家具放置在哪里?

Informant 009

Living room, bedroom

客厅、卧室

Researcher

Is your custom furniture all solid wood?

您家的定制家具都是实木的吗？

Informant 009

Yes. Solid wood furniture can be used for a long time and has a better texture. Compared to panel furniture, the pollution will also be a little less.

是的。实木家具可以用很久，比较有质感。相较于普通的板式定制家具，污染也会小一点。

Researcher

What is your custom furniture style like? Is it consistent with the decoration style of the home?

您家定制家具风格是什么样？和家中装修风格一致吗？

Informant 009

New Chinese style, more consistent

新中式、较为一致

Researcher

What is your understanding of custom furniture?

您对定制家具的理解是什么？

Informant 009

Personalized furniture configuration customized according to personal preferences and space details. Furniture is designed and manufactured according to consumer demand.

根据个人喜好、空间细节，定做的个性化家具配置，定制家具企业会根据消费者的需求来设计家具并制作。

Researcher

What do you know about the custom furniture brand channel?

您了解定制家具品牌渠道是什么?

Informant 009

TV commercials, and roadside billboards and so on

电视广告，还有路边的广告牌等等

Researcher

How did you learn about custom furniture?

您是怎么了解定制家具相关内容?

Informant 009

Ask furniture stores or furniture manufacturers

去家具卖场和家具制作工厂询问

Researcher

What was your initial impression of the brand you chose? What was the initial understanding?

您对您选择的品牌最初印象是什么？最初的理解是什么？

Informant 009

Good quality, there is a certain after-sales guarantee

质量不错，有一定的售后保障

Researcher

Why did you choose the brand's bespoke furniture?

您选择该品牌的定制家具的原因是什么？

Informant 009

Recommendation from friends

朋友推荐

Researcher

What do you think are the advantages of custom-made furniture over finished furniture?

您认为相比成品家具，定制家具的优势是什么?

Informant 009

Can better meet the needs of consumers

可以更加满足消费者的需求

Researcher

What do you think you should pay attention to when choosing custom furniture?

您觉得在选择定制家具时应该注意什么问题？

Informant 009

Ask well in advance for a good price, as well as installation and shipping responsibility. In addition, as with all homes, pay attention to the corners and corners of the furniture to prevent bumping into the children in the house. If the home has sharp corners, my wife will buy anti-bump tape to cover the corners.

提前询问好价格，以及安装和运输如何负责。另外和所有的家具有一样，注意家具的边边角角，防止磕碰的家里的小朋友。如果家具有尖角的，我的妻子都会购买防磕碰胶带将拐角遮住。

Researcher

How often do you use custom furniture?

您使用定制家具的频率是如何的？

Informant 009

About twice a day

一天两次左右

Researcher

What is the way your custom furniture opens and closes doors?

您家定制家具开关门方式是什么样的？

Informant 009

Push-pull.

推拉式。

Researcher

Will you share your renovation success with others?

您会与别人分享您的装修成功经验吗?

Informant 009

Yes

会

Researcher

What do you think are the disadvantages of current custom furniture?

您觉得当前的定制家具的缺点是什么?

Informant 009

Expensive, long production cycle and do not know the progress, cumbersome design

价格昂贵，制作周期长且不知道进度，设计繁琐

Researcher

So do you expect factories to be transparent about the production cycle and even the production process?

所以您会期待工厂将生产周期甚至生产过程透明化吗？

Informant 009

I would love to have this feature, but this may be too difficult for the factory to implement.

很希望能有这个功能，但是这个对工厂来说可能太难实现了。

Researcher

What other features do you think custom furniture can add?

您觉得定制家具可以添加什么其他功能？

Informant 009

Mezzanine, stratify the space, make full use of the space, improve the storage space

and use efficiency. It would be nice if the shelves could be moved. Consumers can not only easily adjust the division of interior space according to their own needs, but also use furniture for different occasions. In addition, for custom furniture such as wardrobes, you can also add light inside. When looking for clothing, lighting can greatly improve the user's experience and make it easier for users to find the clothes they need. Moreover, users can freely adjust the brightness and color temperature of the light according to their needs to achieve a more comfortable use effect.

夹层，将空间分层，充分利用空间，提高收纳空间和使用的效率。如果层板可以活动的话那就太好了。消费者不仅可以方便地根据自己的需求来调整内部空间的划分，同时也可将家具用于不同的场合。另外，对于衣柜这类定制家具而言，还可以在里面增加灯光。在寻找衣物时，灯光可以大大提高使用者的使用体验，让用户更方便地寻找到自己需要的衣物。而且，使用者也可以根据需要自由地调节灯光的亮度和色温，达到更加舒适的使用效果。

Researcher

What aspects of custom furniture can provide users with more possibilities?

定制家具的哪些方面可以为用户提供更多的可能性?

Informant 009

Intelligence, interaction, interlayer concealment. The concealment of the sandwich can make the furniture more beautiful, reduce some excessive exposure, and improve the overall beauty.

智能、交互、夹层隐蔽性。夹层隐蔽性则可以使家具更加美观，减少一些过度的外露，提高整体的美感。

Researcher

What do you mean by intelligence and interaction?

您所说的智能和交互是指哪些呢？

Informant 009

For example, the light in the wardrobe is a sensor light that can be turned on when the door is opened. In terms of interaction, I hope that there can be an interactive screen outside the wardrobe to show today's temperature, and then make recommendations based on today's temperature and the clothes I have. There is also a smart mirror set up outside the wardrobe, virtual fitting, and a dress-up.

比如，衣柜内的灯光是个感应式灯光，可以在开门的时候才亮起。交互的话，我是希望衣柜外面可以有一块可以互动的屏幕，展示今天的温度，然后根据今天的温度和我所拥有的衣服进行搭配推荐。还有在衣柜外面设置一块智能镜子，虚拟试衣，一件换装。

Researcher

Ok, thanks for the valuable suggestion. That's the end of our interview, thank you for your cooperation.

好的，感谢您提出的宝贵建议。我们的访谈就结束了，谢谢您的配合。
