# Supplementary material for: Building a 4E interview-grounded theory model: A case study of demand factors for customized furniture
Source: PLoS One. 2023 Apr 27;18(4):e0282956. doi: 10.1371/journal.pone.0282956 (PMC10138260; doi:10.1371/journal.pone.0282956)
Supplement: S1 File — (ZIP) [file pone.0282956.s001.zip › transcript/transcript 010.pdf]

Informant : 010

Please note that the original transcript is in Simplified Chinese. The English translation is for internal communication among the author of this research, and it is not proofread. Potential linguistic errors may exist in the English translation.

Researcher

Thank you for your willingness to participate and be interviewed here. My name is XXX, and I’m a PhD in the XXX University. Currently, I am working on a research project that focuses on collecting information about user demand when purchasing and using customized furniture. Throughout the interview, I will ask you a series of questions and you are encouraged to express your opinions and views freely. During the interview, I will ask you if I have questions about what you have said or if I need you to clarify a topic or concept.

感谢您愿意参加并在此接受采访。我叫 XXX，是 XXX 大学的博士。目前，我正在开展一个研究项目，主要收集在使用定制家具时的用户体验资料。在整个访谈中，我会问您一系列问题，我们鼓励您自由表达您的意见和观点。在访谈过程中，如果我对你所说的内容有疑问或需要您澄清一个主题或概念，我会向您询问。

Researcher

Are you ready?

您准备好了吗?

Informant 010

Yes.

准备好了。

Researcher

First of all, How old are you now?

请问您现在的年龄是多少？

Informant 010

I am 32 years old.

我今年 32 岁。

Researcher

What kind of work are you doing now?

请问您现在从事什么工作呢？

Informant 010

I am a company manager.

我是一个司机。

Researcher

What is the area of your house?

你的房子的面积是多少?

Informant 010

140 sq. ft.

140 平方。

Researcher

How many people are in your household? What does the family structure look like?

您的家庭人数？家庭结构是什么样的？

Informant 010

4 people; I, wife, two children

4 人；我，妻子，两个孩子

Researcher

What style of furniture is in the home?

家中家具是什么样式的？

Informant 010

Simple and modern, there are also Chinese-style chairs

简约现代，也有中式的椅子

Researcher

Where is the custom furniture placed? Which cabinets are the main ones?

定制家具放置在哪里？主要是哪些柜体？

Informant 010

Mainly placed in the bedroom; Bedside tables, wardrobes, bookcases

主要是放在卧室；床头柜，衣柜，书柜

Researcher

Isn't there a kitchen?

厨房没有吗？

Informant 010

Ah, and the kitchen also has cupboards. I don't cook very often, so I forgot.

啊，厨房也是有橱柜的。我不经常做饭，所以忘记了。

Researcher

What is your custom furniture style like?

您家定制家具风格是什么样？

Informant 010

Modern minimalist style, basically my wife's choice; It is consistent with the style of finishing at home.

现代简约风格，基本上都是我妻子做的选择；和家里的整理风格是一致的。

Researcher

How much do you spend on custom furniture?

你花多少钱在定制家具上?

Informant 010

40,000 yuan.

4 万元。

Researcher

What is your understanding of custom furniture?

您对定制家具的理解是什么？

Informant 010

Custom-made furniture according to the needs of your own family and the size and style of your house

根据自己家庭的需求以及房子的面积和风格，定制的家具

Researcher

What do you know about the custom furniture brand channel?

您了解定制家具品牌渠道是什么?

Informant 010

Advertising, TV, home improvement company recommended

广告，电视，家装公司推荐

Researcher

How did you learn about custom furniture?

您是怎么了解定制家具相关内容?

Informant 010

Shopping guide, network

导购，网络

Researcher

So will you listen carefully to the advice of the shopping guide?

所以您会认真听导购的建议吗？

Informant 010

Yes, but it will not be completely listened to, will shop around and listen to the advice of different shopping guides.

是的，但也不会完全听信，会货比三家，听不同导购员的建议。

Researcher

What was your initial impression of the brand you chose?

您对您选择的品牌最初印象是什么?

Informant 010

Public praise, price and appearance

口碑，价格和外观

Researcher

Why did you choose the brand's bespoke furniture?

您选择该品牌的定制家具的原因是什么？

Informant 010

Price and quality control. For this price, I think there is no problem. Their brand is not very famous. In terms of quality, I think it's okay, after all, even if it is cheap at this price, the quality will not be bad.

价格和品控。对于这个价格，我是觉得没什么问题的。他们的品牌不是很出名。质量方面我觉得也还行吧，毕竟这个价位就算是便宜的，质量也不会差到哪里去。

Researcher

What do you think are the advantages of custom-made furniture over finished furniture?

您认为相比成品家具，定制家具的优势是什么?

Informant 010

Customized furniture can be designed according to the needs of the owner, the appearance and size of the corresponding furniture can be individually designed, so as to meet the individual needs of each owner. Strong selectivity, in the choice of furniture, customized furniture in the selection, application and applicability and other aspects, have a strong performance, compared with finished furniture, it is more suitable for owners who like personalized furniture.

定制家具可以根据业主需求，对相应家具的外观、尺寸进行个人 DIY 设计，从而满足每一位业主的个性需求。选择性强，在进行家具选择时，定制家具在选择、应用以及适用性等多个方面，都有着很强的表现，相比成品家具而言，它更适合喜欢个性化家具的业主。

Researcher

What do you think you should pay attention to when choosing custom furniture?

您觉得在选择定制家具时应该注意什么问题？

Informant 010

The type and size of customized furniture products should be determined according to the area of the room. If the area of the house is limited, it is necessary to choose space-saving, as simple as possible, and the volume should be relatively small. Pay more attention to the use of space so that it does not appear crowded. It should be unified with the decoration style.

要根据居室面积确定定制家具产品的种类和大小。如果房子面积有限，就要选择节省空间的，尽量简单，体量也要相对小些。更加注重空间的运用，这样才不会显得拥挤。要与装修风格统一。

Researcher

How often do you use cabinets, wardrobes, and other custom furniture?

您使用橱柜、衣柜、和其他定制的家具的频率是如何的？

Informant 010

Use it every day

每天都使用

Researcher

What is the way your custom furniture opens and closes doors?

您家定制家具开关门方式是什么样的？

Informant 010

Push and pull and open flat.

推拉和平开。

Researcher

Which way do you prefer to open and close doors?

您喜欢哪种开关门方式？

Informant 010

Push and pull. Sliding doors saves effort and space.

推拉。推拉开门比较省力和省空间。

Researcher

Your sliding door has been used for a long time, will it be unsmooth?

您家推拉门用久了，会出现不顺滑的状况吗？

Informant 010

Not yet. The sliding door is not good for this, it will not be smooth after a long time, and it will also produce noise. Therefore, the track of the sliding door should still be selected as well as possible.

暂时还没有。推拉门就是有这点不好，用久了会不顺滑，还会产生噪音。所以推拉门的轨道还是要尽量挑选好一点的。

Researcher

Will you share your renovation success with others?

您会与别人分享您的装修成功经验吗?

Informant 010

Yes

会

Researcher

Do you have any experience to share with us?

那您有什么经验要给我们分享吗？

Informant 010

Before production, it is best to let the designer come up with a scheme drawing and have a better communication with their own ideas, so that the size will be better grasped. If you pay attention to details, you can consider comprehensive consideration of custom furniture from the color, pattern, material and other aspects of the home before customizing furniture. In addition, it is necessary to consider its service life and utilization as well as maintenance costs.

在生产前，最好能让设计师出一个方案图，和自己的想法有一个比较好的沟通，这样在尺寸上会更好把握一些。如果你很注重细节，在定制家具前可以考虑从家

居的颜色、花纹、材质等方面综合考虑定制家具。此外，还要考虑好它的使用寿命和利用率以及维修成本。

Researcher

What do you think are the disadvantages of current custom furniture?

您觉得当前的定制家具的缺点是什么?

Informant 010

Expensive.

价格贵。

Researcher

What other features do you think custom furniture can add?

您觉得定制家具可以添加什么其他功能？

Informant 010

some odor details; You can apply more functional hardware; The design is more user-friendly.

一些气味细节；可以多应用一些功能五金；设计更加人性化。

Researcher

What is functional hardware?

功能五金是指什么？

Informant 010

It's handles, clothes rails, baskets, bouncers, trouser racks, shelves and so on. If the wardrobe is installed with clothes rails and trouser racks according to the height and habits of the user, it can allow users to better store and save space.

就是拉手、挂衣杆、拉篮、反弹器、裤架、置物架等等。衣柜根据用户高度与习惯安装挂衣杆、裤架这些东西的话，可以让用户进行更好的收纳，节省空间。

Researcher

What aspects of custom furniture can provide users with more possibilities?

定制家具的哪些方面可以为用户提供更多的可能性?

Informant 010

Save space, pay more attention to the use of space, and will not appear crowded; Unified with the decoration style, more beautiful, there are more possibilities.

节省空间，更加注重空间的运用，不会显得拥挤；与装修风格统一，更美观，有更多可能。

Researcher

好的，谢谢您接受我们本次的访谈，祝您生活愉快。

Okay, thank you for this interview and have a nice life.
