# Supplementary material for: Building a 4E interview-grounded theory model: A case study of demand factors for customized furniture
Source: PLoS One. 2023 Apr 27;18(4):e0282956. doi: 10.1371/journal.pone.0282956 (PMC10138260; doi:10.1371/journal.pone.0282956)
Supplement: S1 File — (ZIP) [file pone.0282956.s001.zip › transcript/transcript 011.pdf]

Informant : 011

Please note that the original transcript is in Simplified Chinese. The English translation is for internal communication among the author of this research, and it is not proofread. Potential linguistic errors may exist in the English translation.

Thank you for your willingness to participate and be interviewed here. My name is XXX, and I’m a PhD in the XXX University. Currently, I am working on a research project that focuses on collecting information about user demand when purchasing and using customized furniture. Throughout the interview, I will ask you a series of questions and you are encouraged to express your opinions and views freely. During the interview, I will ask you if I have questions about what you have said or if I need you to clarify a topic or concept.

感谢您愿意参加并在此接受采访。我叫 XXX，是 XXX 大学的博士。目前，我正在开展一个研究项目，主要收集在使用定制家具时的用户体验资料。在整个访谈中，我会问您一系列问题，我们鼓励您自由表达您的意见和观点。在访谈过程中，如果我对你所说的内容有疑问或需要您澄清一个主题或概念，我会向您询问。

Researcher

Are you ready?

您准备好了吗？

Informant 011

Yes.

准备好了。

Researcher

What is the square footage of your house?

你的房子的面积是多少?

Informant 011

About 105 square meters.

大约 105 平方米.

Researcher

How big is your family? What's the family structure like?

您的家庭人数？家庭结构是什么样的？

Informant 011

Three people, my parents and me

3 人，父母和我

Researcher

What is the style of furniture in the home?

家中家具是什么样式的？

Informant 011

Chinese furniture, more retro and elegant style.

中式家具，比较复古典雅的风格。

Researcher

Where is the custom furniture placed? What are the main cabinets?

定制家具放置在哪里？主要是哪些柜体？

Informant 011

In the master and assistant bedrooms, there are wardrobes and TV tables. It is an efficient living environment.

主副卧室里，有衣柜和电视桌。是一个高效率的居住环境。

Researcher

What is your custom furniture style? Is it consistent with the home decor?
您家定制家具风格是什么样？和家中装修风格一致吗？

Informant 011

Are Chinese or new Chinese style, and home decoration style is not inconsistent, as far as possible color and material are consistent, so more harmonious.
都是中式或者新中式，和家中装修风格不违和，尽量颜色和材质都保持一致，这样比较和谐。

Researcher

How much do you spend on custom furniture?
你花多少钱在定制家具上?

Informant 011

No more than 20,000 yuan, comfort as the first priority, I consider the cost performance is good.
不超过两万元，普通家庭，并且以舒适为第一位，考虑性价比比较多。

Researcher

What is your understanding of custom furniture?
您对定制家具的理解是什么？

Informant 011

According to their own needs and the state of their own environment to design customized, can solve some problems before the furniture, the use of more comfortable.
根据自己的需求和自家环境的状况来进行设计定制，可以解决以前家具的一些问题，使用更加舒服。

Researcher

What do you know about custom furniture brand channels? (advertising or otherwise)
您了解定制家具品牌渠道是什么?（广告或其他）

Informant 011

TV ads, friend introductions, usually chat with friends at home who are decorating, and they will be invisibly recommended when they just chat.
电视广告，朋友介绍，平时会跟家里在装修的朋友聊聊天，刚好聊到就会被无形地安利。

Researcher

How do you know about custom furniture?
您是怎么了解定制家具相关内容?

Informant 011

The introduction of the receptionist when buying furniture, the Internet search, the friends to share some experience, it is just to ask a lot, shop around.
购买家具时接待人员的介绍，网络搜寻，朋友交谈分享一些经验，反正就是多问问，货比三家。

Researcher

What was your initial impression of the brand you chose?
您对您选择的品牌最初印象是什么?

Informant 011

Brand reputation is good, friends around are buying, cost-effective
品牌口碑较好，身边朋友的都在买，性价比较高

Researcher

What was your initial impression of the brand you chose? What was the initial understanding?

您选择该品牌的定制家具的原因是什么？

Informant 011

Provide more satisfactory service and price, can be designed according to their own personal needs, especially the home with the elderly and children can be more safe and comfortable.

提供较为满意的服务与价格，能根据自己的个人需求进行设计，特别是家里有老人小孩就可以更加安全舒适。

Researcher

What do you think are the advantages of custom furniture over finished furniture?

您认为相比成品家具，定制家具的优势是什么?

Informant 011

According to the needs of different users and different use of the environment for flexibility and adjustment, personalized, systematic. Because today's young people like exclusive, unique, will have a sense of belonging.

能够根据不同用户的需求和不同的使用环境进行灵活变通与调节，个性化，系统化。因为现在的年轻人都喜欢专属的，独特的，会有一种归属感。

Researcher

What do you think you should pay attention to when choosing custom furniture?

您觉得在选择定制家具时应该注意什么问题?

Informant 011

Brand, size, price, workmanship, material, waiting time, these are all considered.

品牌，尺寸，价格，做工，材质，等待时间，这些都要考虑。

Researcher

How often do you use cabinets, closets, and other custom furniture?

您使用橱柜、衣柜、和其他定制的家具的频率是如何的？

Informant 011

And normal furniture, is the daily collocation of clothes, the use of storage function, put some daily necessities.

和正常家具无异，就是日常搭配衣服，使用储物功能，放一些日用品之类的。

Researcher

Does the appearance of current custom furniture products meet your needs?

当前定制家具产品外观满足您的需求吗?

Informant 011

较为满意

More satisfied

Researcher

当前定制家具产品触觉细节满足您的需求吗?

Informant 011

The more details, the higher the price required, cost-effective is the focus of customer consideration

细节越多，所需的价钱越高，性价比是客户考虑的重点

Researcher

Does the current custom furniture fit your functional needs? Which need is not being met?

当前的定制家具是否符合您对产品功能的需求？哪一个需求没有得到满足？

Informant 011

Most of the requirements can be met, but some small details can not be fully realized,

because it may cost a lot of manpower and resources to achieve, there are some limitations.
大部分需求都能满足，但是有些小细节无法完全实现，因为可能会耗费大量的人力物力去达成，还是有些限制的。

Researcher
Does the current custom furniture meet your need for product audibility or smell?
当前定制家具是否符合您对产品可听性或气味的需求？

Informant 011
The smell of customized furniture is heavy and it takes more time to volatilize formaldehyde
定制家具的气味较重，需要较多时间来挥发甲醛

Researcher
How do you open and close your custom furniture? How do you like to open and close the door?
您家定制家具开关门方式是什么样的？您喜欢哪种开关门方式？

Informant 011
Sliding door between kitchen and dining room. The sealing of the sliding door is very good, will not let the smoke in the kitchen run to the restaurant, will not let the taste of the restaurant run to the kitchen, but also can play the role of dust isolation.
厨房和餐厅之间，用推拉门。推拉门的密封性很好，不会让厨房中的油烟跑到餐厅中，也不会让餐厅中的味道跑到厨房中，同时还能够起到隔绝灰尘的作用。

Researcher
Will you share your successful decorating experience with others?
您会与别人分享您的装修成功经验吗?

Informant 011

当然，如果别人询问的话。我也愿意在一些公共网络平台分享一些经验。

Of course, if people ask. I am also willing to share my experience on some public online platforms.

Researcher

What do you think are the disadvantages of current custom furniture?

您觉得当前的定制家具的缺点是什么?

Informant 011

The design takes a long time to make, the price is high, and is not completely satisfied with the customization, is a limited customization.

设计做成所需时间较长，价格较高，并且不是完全满意的定制，是有限制的定制。

Researcher

What other features do you think can be added to custom furniture?

您觉得定制家具可以添加什么其他功能？

Informant 011

Detachable and replaceable means that the product design or parts design has the ability of convenient disassembly, easy replacement and easy maintenance. Specifically, it means that the design of the parts should be easy to disassemble and replace, but also easy to maintain. With this performance, it can make the product easier to repair, can save more materials, and thus reduce the production cost of the product.

可拆卸更换性是指产品设计或零部件设计具有拆卸方便、更换容易，便于维修的能力。具体来说，就是指零部件的设计要便于拆卸和更换，同时还要方便维修。有了这一性能，就能使产品更易维修，能节省更多的材料，从而减少产品的生产成本。

Researcher

What aspects of custom furniture can provide more possibilities for users?

定制家具的哪些方面可以为用户提供更多的可能性?

Informant 011

Adapt to the user's self-creativity, let the user to create

适应用户的自我创造性，让用户自己来创造

Researcher

These are all the questions. Thank you very much for participating in our research.

这就是全部的问题。 非常感谢您参与我们的研究。
