# Supplementary material for: Building a 4E interview-grounded theory model: A case study of demand factors for customized furniture
Source: PLoS One. 2023 Apr 27;18(4):e0282956. doi: 10.1371/journal.pone.0282956 (PMC10138260; doi:10.1371/journal.pone.0282956)
Supplement: S1 File — (ZIP) [file pone.0282956.s001.zip › transcript/transcript 012.pdf]

**Informant : 012**

***Please note that the original transcript is in Simplified Chinese. The English translation is for internal communication among the author of this research, and it is not proofread. Potential linguistic errors may exist in the English translation.***

Researcher

Thank you for your willingness to participate and be interviewed here. My name is XXX, and I'm a PhD in the XXX University. Currently, I am working on a research project that focuses on collecting information about user demand when purchasing and using customized furniture. Throughout the interview, I will ask you a series of questions and you are encouraged to express your opinions and views freely. During the interview, I will ask you if I have questions about what you have said or if I need you to clarify a topic or concept.

感谢您愿意参加并在此接受采访。我叫 XXX，是 XXX 大学的博士。目前，我正在开展一个研究项目，主要收集在使用定制家具时的用户体验资料。在整个访谈中，我会问您一系列问题，我们鼓励您自由表达您的意见和观点。在访谈过程中，如果我对你所说的内容有疑问或需要您澄清一个主题或概念，我会向您询问。

Researcher

What is the square footage of your house?

你的房子的面积是多少?

Informant 012

Because in the village, the home area is large, and the usual area is 150 square meters

因为在村子里 家里面积较大 常用面积 150 平方

Researcher

How big is your family? What's the family structure like?

您的家庭人数？家庭结构是什么样的？

Informant 012

家庭人数为三人（父母和我）核心家庭主要由父母和未婚子女组成 姐姐偶尔回家居住

The family size is three people (parents and me), the nuclear family consists mainly of parents and unmarried children, and the sister occasionally returns home to live

Researcher

What is the style of furniture in the home?

家中家具是什么样式的？

Informant 012

There are plank wood, solid wood furniture and other soft furnishings. In the style of home furniture, it is mainly pastoral style furniture and old Chinese style. Furniture products are mainly purchased according to the interior decoration style.

有板木、实木家具以及其他软装。在家庭家具风格中主要以田园风格家具和旧中式风格为主。主要按照室内的装修风格选购家具产品。

Researcher

Where is the custom furniture placed? What are the main cabinets?

定制家具放置在哪里？主要是哪些柜体？

Informant 012

Custom furniture is mainly placed in areas such as kitchens and bathrooms. In particular, the problem of oil stains in the kitchen, whether it is easy to clean up after adhering to the furniture, and moisture-proof measures in the bathroom. The cabinet body is mostly a parallel double door to pull ring double door

定制家具主要放置在厨房以及卫生间等区域。特别是厨房的油污问题，在附着在家具上以后是否会容易清理以及卫生间的防潮措施等。柜体的话多是平行双开门

以拉环式双开门。

Researcher

What is your custom furniture style? Is it consistent with the home decor?

您家定制家具风格是什么样？和家中装修风格一致吗？

Informant 012

Simple, generous style, compound traditional Chinese style and pastoral style; consistent

简朴，大方的风格，复合传统中式和田园式风格；一致。

Researcher

How much do you spend on custom furniture?

你花多少钱在定制家具上?

Informant 012

4000~5000 yuan in custom furniture

4000～5000 元在定制家具中

Researcher

What is your understanding of custom furniture?

您对定制家具的理解是什么？

Informant 012

Tailored to meet the aesthetic needs of different users of products. And through the customized way to design the furniture interior style space. For the users of a higher life experience and convenient living space.

量身定制，满足不同用户的生活审美需求等产品。并且通过定制的方式设计出适合户型内部的家具室内风格空间。为用户更高的生活体验和便利的生活空间。

Researcher

What do you know about custom furniture brand channels? (advertising or otherwise)

您了解定制家具品牌渠道是什么?

Informant 12

Mainly online, through marketing advertisements, or recommended by well-known bloggers, or through Xiaohongshu Zhihu or other APP to understand customized furniture

主要还是线上通过营销广告或者是知名博主推荐又或者是通过小红书知乎或者是其他 APP 了解定制家具

Researcher

How do you know about custom furniture?

您是怎么了解定制家具相关内容?

Informant 012

The whole house customization of some wardrobe, bookcases, wine cabinets, shoe cabinet storage cabinets based customization, and more other different furniture products customization. Create independent, unified and personalized space for users to live and activity space. Meet the individual needs of different consumers.

全屋定制一些衣柜，书柜，酒柜，鞋柜储物柜为主的定制，还有更多其他不同的家具产品定制。为用户居住和活动空间创造出独立，统一，个性的空间。满足不同消费者的个性需求。

Researcher

What was your initial impression of the brand you chose? What was the initial understanding?

您对您选择的品牌最初印象是什么? 最初的理解是什么？

Informant 012

Provide users with timely solutions and provide free personality service suggestions for the pain points of the householder. Intimate, automated to provide solutions and budget, output modification suggestions one-to-one questions and services
为用户提供及时的解决方案及为户主关心的痛点提供免费个性等服务建议。贴心，自动化提供解决方案及预算，产出修改建议一对一答疑及服务

Researcher
Why do you choose this brand of custom furniture?
您选择该品牌的定制家具的原因是什么？

Informant 012
The budget is reasonable, safe and reliable, friends around have customized the brand and have a good reputation in the market
预算合理，安全可靠，身边朋友有定制过该品牌且市面上口碑较好

Researcher
What do you think are the advantages of custom furniture over finished furniture?
您认为相比成品家具，定制家具的优势是什么?

Informant 012
满足了不同用户对于不同产品的不同需求，使家具产品真正意义上做到了为人服
It meets the different needs of different users for different products, so that furniture products can truly serve people, and on the basis of using customized furniture, the safety and environmental protection of products are also guaranteed, so as to be more adapted to user needs.
务，同时在使用定制家具的基础上，产品的安全环保性也得到保障，从而更加适应用户需求。

Researcher
What do you think you should pay attention to when choosing custom furniture?

您觉得在选择定制家具时应该注意什么问题？

Informant 012

使用的材料、价格、环保性以及后续使用的实用性及寿命

Materials used, price, environmental friendliness and practicality and longevity of subsequent use

Researcher

How often do you use cabinets, closets, and other custom furniture?

您使用橱柜、衣柜、和其他定制的家具的频率是如何的？

Informant 012

橱柜和衣柜较多使用，主要以厨房定制家具使用频率较高，其次便是餐座区域，学习区域，喝茶区域所涉及的座椅运用较多。

Cabinet and wardrobes are often used, mainly with the kitchen customized furniture, followed by the seating area, learning area, and tea area involved in the use of seats.

Researcher

Does the appearance of current custom furniture products meet your needs?

当前定制家具产品外观满足您的需求吗?

Informant 012

勉强满足，勉强满足家庭装修风格的协调统一。

Barely meet, reluctantly meet the coordination and unity of the family decoration style.

Researcher

Do current custom furniture products meet your needs with tactile details?

当前定制家具产品触觉细节满足您的需求吗?

Informant 012

一般般，定制家具想要满足需求的同时也要考虑价格以及最高性价比、

In general, custom furniture wants to meet the needs while considering the price and the best value for money

Researcher

Does the current custom furniture fit your functional needs? Which need is not being met?

当前的定制家具是否符合您对产品功能的需求？哪一个需求没有得到满足？

Informant 012

复合，储物功能使用寿命长的方面没有得到满足。

The compound, storage function of the long service life is not met.

Researcher

Does the current custom furniture meet your need for product audibility or smell?

当前定制家具是否符合您对产品可听性或气味的需求？

Informant 012

不符合。

inconformity

Researcher

How do you open and close your custom furniture? How do you like to open and close the door?

您家定制家具开关门方式是什么样的？您喜欢哪种开关门方式？

Informant 012

平开门隐藏半隐蔽式，平开门式

Flat door hidden semi-hidden, flat door type

Researcher

Will you share your successful decorating experience with others?

您会与别人分享您的装修成功经验吗?

Informant 012

会

yes

Researcher

What do you think are the disadvantages of current custom furniture?

您觉得当前的定制家具的缺点是什么?

Informant 012

美观和功能上较为常规，个性化程度不高。

Beauty and function is more conventional, personalized degree is not high

Researcher

What other features do you think can be added to custom furniture?

您觉得定制家具可以添加什么其他功能？

Informant 012

隐藏收纳式功能以及细节安全方面的考虑应当更全面些。也可添加智能化功能

Hidden storage type function and detail security aspect of the consideration should be more comprehensive.Intelligent functions can also be added

Researcher

What aspects of custom furniture can provide more possibilities for users?

定制家具的哪些方面可以为用户提供更多的可能性?

Informant 012

材料，舒适性，性价比高低，独特性私人定制打造性为用户提供更多可能行。

Materials, comfort, cost performance, unique private customization to provide users with more possibilities.

Researcher

Thank you very much for participating in our interview, thank you.

非常感谢您参与我们的访谈，谢谢。
