# Supplementary material for: Building a 4E interview-grounded theory model: A case study of demand factors for customized furniture
Source: PLoS One. 2023 Apr 27;18(4):e0282956. doi: 10.1371/journal.pone.0282956 (PMC10138260; doi:10.1371/journal.pone.0282956)
Supplement: S1 File — (ZIP) [file pone.0282956.s001.zip › transcript/transcript 013.pdf]

Informant : 013

Please note that the original transcript is in Simplified Chinese. The English translation is for internal communication among the author of this research, and it is not proofread. Potential linguistic errors may exist in the English translation.

Thank you for your willingness to participate and be interviewed here. My name is XXX, and I'm a PhD in the XXX University. Currently, I am working on a research project that focuses on collecting information about user demand when purchasing and using customized furniture. Throughout the interview, I will ask you a series of questions and you are encouraged to express your opinions and views freely. During the interview, I will ask you if I have questions about what you have said or if I need you to clarify a topic or concept.

感谢您愿意参加并在此接受采访。我叫 XXX，是 XXX 大学的博士。目前，我正在开展一个研究项目，主要收集在使用定制家具时的用户体验资料。在整个访谈中，我会问您一系列问题，我们鼓励您自由表达您的意见和观点。在访谈过程中，如果我对你所说的内容有疑问或需要您澄清一个主题或概念，我会向您询问。

Researcher

What is the square footage of your house?

你的房子的面积是多少?

Informant 013

130 平米

130 square meters

Researcher

How big is your family? What's the family structure like?

您的家庭人数？家庭结构是什么样的？

Informant 013

家庭人数为三，家庭结构为核心家庭，分别为父亲母亲和我

There are three people in the family. The family structure is a nuclear family, consisting of my father, mother and myself.

Researcher

What is the style of furniture in the home?

家中家具是什么样式的?

Informant 013

家中家具整体风格偏欧式简约风，但也有结合中式元素;属性主要为实木家具和板式家具

The overall style of furniture in the home leans towards European simplicity, but also combines Chinese elements; Properties are mainly solid wood furniture and panel furniture.

Researcher

Where is the custom furniture placed? What are the main cabinets?

定制家具放置在哪里?主要是哪些柜体?

Informant 013

由于整体装修风格是全屋定制的，部分定制家具分布不均，主要放置在卧室和客厅，主要柜体为衣橱还有茶几柜，电视柜，书柜，酒柜。

As the overall decoration style is customized for the whole house, some customized furniture is not evenly distributed, mainly placed in the bedroom and living room. The main cabinets are wardrobes, tea table cabinets, TV cabinets, bookcases and wine cabinets.

Researcher

What is your custom furniture style? Is it consistent with the home decor?

您家定制家具风格是什么样?和家中装修风格一致吗?

Informant 013

定制家具风格为木式家具和偏欧式家具，和家中装修风格不违和，是配套定制的。

Custom furniture style for wood furniture and partial European furniture, and home decoration style is compatible, is customized.

Researcher

How much do you spend on custom furniture?

你花多少钱在定制家具上?

Informant 013

具体价格不太清楚，因为是父母选择的。

The exact price is unclear because parents choose it.

Researcher

What is your understanding of custom furniture?

您对定制家具的理解是什么？

Informant 013

In my opinion, customized furniture is made according to the needs and preferences of residents and tailored for individual customers. At present, it is increasingly becoming the mainstream of the home decoration industry. It is in line with personalized consumption and can integrate customers' ideas with brand concepts and products, gradually increasing sales in the market and recognized by people.

我认为定制家具就是根据住户需要和喜好进行制作，为个别客户量身裁剪的家具，现阶段越来越成为家装行业的主流，符合个性化消费，可以将客户想法同品牌理念和商品融合起来，在市场上的销量也逐步提升，被人们认可。

Researcher

What do you know about custom furniture brand channels?

您了解定制家具品牌渠道是什么?

Informant 013

The channels to learn about customized furniture brands are visiting brand furniture stores, online publicity, brand public accounts, furniture design books and recommendations from friends around.

了解定制家具品牌的渠道是参观品牌家具城、网络宣传、品牌公众号、家具设计类书籍还有周边的朋友推荐。

Researcher

How do you know about custom furniture?

您是怎么了解定制家具相关内容?

Informant 013

Learn about customized furniture by browsing XiaoHongshu or furniture APP software, search furniture brands on wechat public account to learn about different furniture brand design styles, and take time to visit furniture city and let the shopping guide give professional explanation.

通过浏览小红书或者家具类 APP 软件了解定制家具的相关信息，微信搜索公众号家具品牌了解不同家具品牌设计风格，抽时间去家具城参观，让导购进行专业的讲解。

Researcher

What was your initial impression of the brand you chose? What was the initial understanding?

您对您选择的品牌最初印象是什么？最初的理解是什么？

Informant 013

The brands chosen by my family are Gold Medal and Boloni. My initial impression is their environmental protection and overall texture and design sense. The initial understanding is that they use imported boards, beautiful and easy to use.

我家选择的品牌是金牌和博洛尼，最初的印象是看中了它们的环保型以及整体质感和设计感;最初的理解就是它们采用进口的板子，好看也好用。

Researcher

Why do you choose this brand of custom furniture?

您选择该品牌的定制家具的原因是什么？

Informant 013

The combination of these two furniture brands is chosen because every place in the kitchen, porch, bookcase and bedroom can be taken care of, and the quality is good.

选择组合这两个家具品牌是因为厨房玄关书柜卧室每个地方都能照顾得到，质量好。

Researcher

What do you think you should pay attention to when choosing custom furniture?

您觉得在选择定制家具时应该注意什么问题?

Informant 013

Customized furniture can be selected according to individual aesthetics and needs. First, the material can be selected at will. The environmental protection material is controllable. The service life of customized furniture will be longer, the aesthetic degree is more fine, many details are guaranteed, and the space utilization rate is high, can be targeted according to the pain point of the house design, these are the finished furniture can not match.

定制家具可以通过个人审美和需求进行选择，首先在材质上可以随心挑选，环保材质是可控的，许多成品家具在环保型上都欠佳，对住户的身体都是有一定伤害的；使用时间上定制家具使用寿命也会更长，美观程度上也更加精细，许多细节也是有所保证的，而且空间利用率高，可以根据房屋痛点进行针对性的设计，这些都是成品家具无法匹敌的。

Researcher

How often do you use cabinets, closets, and other custom furniture?

您使用橱柜、衣柜、和其他定制的家具的频率是如何的?

Informant 013

At home, I use the closet bookcase more frequently and open the door every day. The coffee table cabinet is also used more frequently and the cupboard is used more by my parents.

在家里我使用衣柜书柜的频率较高，每天都会打开柜门，茶几柜使用频率也很高，橱柜父母使用较多。

Researcher

Does the appearance of current custom furniture products meet your needs?

当前定制家具产品外观满足您的需求吗?

Informant 013

当前定制的家具产品外观基本上符合我父母的审美和功能需求，同我个人审美还是有一定偏差的，我个人倾向于现代简约风。

The appearance of the current customized furniture products is basically in line with my parents' aesthetic and functional needs, and there is a certain deviation from my personal aesthetic, I personally tend to modern simple style.

Researcher

Do current custom furniture products meet your needs with tactile details?

当前定制家具产品触觉细节满足您的需求吗?

Informant 013

产品触觉细节可以满足我的需求，因为是实木材质，木头是有温度的，且四季都会给人不同的触感。

The tactile details of the product can meet my needs, because it is made of solid wood, wood has temperature, and the four seasons will give people different tactile sensations.

Researcher

Does the current custom furniture fit your functional needs? Which need is not being met?

当前的定制家具是否符合您对产品功能的需求？哪一个需求没有得到满足？

Informant 013

It basically meets my functional requirements of the product, but I am not satisfied with the

diversified functions of the product. I want to meet the appearance and at the same time, it can be assembled into other types of furniture, which can be deformed and have stronger functions.
基本符合我对产品功能的需求，但是对于产品功能多元化方面并不是很满意，想满足外观的同时更能组装成别的样式的家具，可变形，功能性更强。

Researcher

Does the current custom furniture meet your need for product audibility or smell?
当前定制家具是否符合您对产品可听性或气味的需求?

Informant 013

基本符合，全家人都很喜欢木质家具的味道。
Basically, the whole family loves the smell of wood furniture.

Researcher

How do you open and close your custom furniture? How do you like to open and close the door?
您家定制家具开关门方式是什么样的?您喜欢哪种开关门方式?

Informant 013

The opening and closing modes are both open and pull. I prefer the cabinet with the flat door, because the cabinet opens outwards, and the whole wardrobe can be displayed in front of my eyes after opening, which is convenient for sorting clothes and smooth and convenient for use.
开关门的方式平开式和推拉式都有。我更喜欢平开门的柜子，因为柜子向外开，打开之后整个衣柜都可以展示在眼前，方便整理衣物，使用顺畅方便。

Researcher

Will you share your successful decorating experience with others?
您会与别人分享您的装修成功经验吗?

Informant 013

Yes, because I thought it would be nice to work together to make things more livable.

会的，因为觉得一起让家里陈设变得更适合居住是很好的事。

Researcher

What do you think are the disadvantages of current custom furniture?

您觉得当前的定制家具的缺点是什么?

Informant 013

The main disadvantage is first of all, the design is cumbersome, the waiting cycle time is too long, if you want to customize a larger wardrobe, it takes a long time to make, if the design does not meet customer requirements, then in the after-sales service more trouble.

主要缺点首先就是设计繁琐，等待的周期时间过长，如果要定制更大型的衣柜等需要长时间制作，如果设计中出现不符合客户要求的设计那么在售后服务上更加麻烦。

Researcher

What other features do you think can be added to custom furniture?

您觉得定制家具可以添加什么其他功能?

Informant 013

I think more high-tech artificial interactive systems can be added, more ergonomic design should be considered, more malleability and storage type changes should be added, so that a customized furniture can be used in a variety of ways.

我觉得可以添加多一些高科技人工交互系统，多考虑符合人体工程学的设计，增加更多延展性和储物型的变换，做到一个定制家具多种使用方法。

Researcher

What aspects of custom furniture can provide more possibilities for users?

定制家具的哪些方面可以为用户提供更多的可能性?

Informant 013

The first is the security aspect, and the second is the use aspect.

首先是安全方面，其次是使用方面。

Researcher

This interview is over this technology, thank you for participating.

本次访谈到此技术，感谢您的参与。
