# Supplementary material for: Building a 4E interview-grounded theory model: A case study of demand factors for customized furniture
Source: PLoS One. 2023 Apr 27;18(4):e0282956. doi: 10.1371/journal.pone.0282956 (PMC10138260; doi:10.1371/journal.pone.0282956)
Supplement: S1 File — (ZIP) [file pone.0282956.s001.zip › transcript/transcript 014.pdf]

Informant : 014

Please note that the original transcript is in Simplified Chinese. The English translation is for internal communication among the author of this research, and it is not proofread. Potential linguistic errors may exist in the English translation.

Thank you for your willingness to participate and be interviewed here. My name is XXX, and I'm a PhD in the XXX University. Currently, I am working on a research project that focuses on collecting information about user demand when purchasing and using customized furniture. Throughout the interview, I will ask you a series of questions and you are encouraged to express your opinions and views freely. During the interview, I will ask you if I have questions about what you have said or if I need you to clarify a topic or concept.

感谢您愿意参加并在此接受采访。我叫 XXX，是 XXX 大学的博士。目前，我正在开展一个研究项目，主要收集在使用定制家具时的用户体验资料。在整个访谈中，我会问您一系列问题，我们鼓励您自由表达您的意见和观点。在访谈过程中，如果我对你所说的内容有疑问或需要您澄清一个主题或概念，我会向您询问。

Researcher

What is the square footage of your house?

你的房子的面积是多少?

Informant 014

139 square meters

139 平方米

Researcher

How big is your family? What's the family structure like?

您的家庭人数？家庭结构是什么样的？

Informant 014

Four people grandma father mother me

4 人 奶奶 父亲 母亲 我

Researcher

What is the style of furniture in the home?

家中家具是什么样式的？

Informant 014

In traditional Chinese furniture, wood is used as the frame and the tenon as the eye. The practice of cleverly connecting the gaps between wood and wood can not only prevent the cracking and deformation of wood, but also has a certain decorative effect.

中国传统家具,以木为框，以榫为眼，将木和木之间的缝隙巧妙地衔接起来的做法，不但可以防止木材的开裂和变形，同时也具有一定的装饰效果。

Researcher

Where is the custom furniture placed? What are the main cabinets?

定制家具放置在哪里？主要是哪些柜体？

Informant 014

卧室，用来放衣服的衣橱

Bedroom, a closet for putting your clothes

Researcher

What is your custom furniture style? Is it consistent with the home decor?

您家定制家具风格是什么样？和家中装修风格一致吗？

Informant 014

没定制过，所以我不太清楚。

No customization, so I don't know.

Researcher

How much do you spend on custom furniture?

你花多少钱在定制家具上?

Informant 014

我觉得未来我需要的话，我的预算应该是 5 万元左右吧。

I think if I need it in the future, my budget should be about 50,000 yuan.

Researcher

What is your understanding of custom furniture?

您对定制家具的理解是什么？

Informant 014

与室内环境较好融合，其形式简洁，稍具纹样装饰点缀。

With indoor environment better fusion, its form is simple, slightly with pattern decoration ornament.

Researcher

What do you know about custom furniture brand channels?

您了解定制家具品牌渠道是什么?

Informant 014

以前在学校有相关课程，还有最近在一些公共社交平台也会刷到相关推荐。

In the past, there were relevant courses in the school, and recently, relevant recommendations have been made on some public social media platforms.

Researcher

How do you know about custom furniture?

您是怎么了解定制家具相关内容?

Informant 014

访问定制家具工厂网站

Visit the Custom Furniture Factory website

Researcher

What was your initial impression of the brand you chose? What was the initial understanding?

您对您选择的品牌最初印象是什么？最初的理解是什么？

Informant 014

价格的适宜亲民 造型的简洁 没有过分的装饰 最初理解：便宜至上

The price is suitable for the people of the simple modeling without excessive decoration initial understanding: cheap supreme

Researcher

Why do you choose this brand of custom furniture?

您选择该品牌的定制家具的原因是什么？

Informant 014

便宜 耐用 整体

Cheap and durable overall

Researcher

What do you think are the advantages of custom furniture over finished furniture?

您认为相比成品家具，定制家具的优势是什么?

Informant 014

Integrity. can be better integrated with the interior space to give people a overall

comfortable feeling

整体性 能够与室内空间较好融合给人整体舒适的感觉

Researcher

What do you think you should pay attention to when choosing custom furniture?

您觉得在选择定制家具时应该注意什么问题?

Informant 014

The price is what I am more concerned about, want to find their own favorite decoration style in their own economic position. Secondly, the technology is also what I value, and I think the texture is very important.

价格是我比较关注的，要在自己的经济方位之内找到自己喜欢的装饰风格。其次工艺也是我比较看重的，我觉得质感很重要。

Researcher

How often do you use cabinets, closets, and other custom furniture?

您使用橱柜、衣柜、和其他定制的家具的频率是如何的?

Informant 014

Ambry uses every day, wardrobe changes season uses

橱柜天天用 衣柜换季用

Researcher

Do current custom furniture products meet your needs with tactile details?

当前定制家具产品触觉细节满足您的需求吗?

Informant 014

Satisenough, because I didn't pay attention to it before, as long as it's not particularly uncomfortable.

一定程度上满足，因为我之前对这方面关注不到，只要不是特别不舒适的就可以了。

Researcher

Does the current custom furniture fit your functional needs? Which need is not being met?

当前的定制家具是否符合您对产品功能的需求？哪一个需求没有得到满足？

Informant 014

Not very satisfied, no more functions, almost all are ordinary cabinets, no additional functions, more boring.

不是很满意，没有更多的功能，几乎都是普通的柜子，没有另外附加的功能，比较枯燥。

Researcher

Does the current custom furniture meet your need for product audibility or smell?

当前定制家具是否符合您对产品可听性或气味的需求？

Informant 014

还可以，气味是木香，听觉上面没有太多感受。

Yes, the smell is wood, and there is not too much feeling on the hearing.

Researcher

How do you open and close your custom furniture? How do you like to open and close the door?

您家定制家具开关门方式是什么样的？您喜欢哪种开关门方式？

Informant 014

Hinges moving type

铰链 移动式

Researcher

Will you share your successful decorating experience with others?

您会与别人分享您的装修成功经验吗?

Informant 014

I'm a sharing person, but I'd be happy to share if someone asks me.

我是个分享欲一般的的人，但是如果别人主动问我我会很乐意分享。

Researcher

What do you think are the disadvantages of current custom furniture?

您觉得当前的定制家具的缺点是什么?

Informant 014

The price is generally relatively high, businesses are too unique to make an issue, which will lead to the price is generally a little inflated.

价格普遍比较高，商家们太拿独一无二来做文章了，这样会导致价格普遍有点虚高。

Researcher

What other features do you think can be added to custom furniture?

您觉得定制家具可以添加什么其他功能？

Informant 014

Classification module by group to receive small objects, but also can add some smart home functions, to keep up with The Times.

按组分类模块收纳小物件，还可以添加一些智能家居的功能，跟上时代。

Researcher

What aspects of custom furniture can provide more possibilities for users?

定制家具的哪些方面可以为用户提供更多的可能性?

Informant 014

Availability and accessibility can be improved. The appearance is now very saturated, and some hidden needs should be concerned.

可用性和无障碍上可以改进。外观现在已经非常饱和了，一些隐性的需求应该被关注到。
