# Supplementary material for: Building a 4E interview-grounded theory model: A case study of demand factors for customized furniture
Source: PLoS One. 2023 Apr 27;18(4):e0282956. doi: 10.1371/journal.pone.0282956 (PMC10138260; doi:10.1371/journal.pone.0282956)
Supplement: S1 File — (ZIP) [file pone.0282956.s001.zip › transcript/transcript 015.pdf]

**Informant : 015**

***Please note that the original transcript is in Simplified Chinese. The English translation is for internal communication among the author of this research, and it is not proofread. Potential linguistic errors may exist in the English translation.***

Thank you for your willingness to participate and be interviewed here. My name is XXX, and I'm a PhD in the XXX University. Currently, I am working on a research project that focuses on collecting information about user demand when purchasing and using customized furniture. Throughout the interview, I will ask you a series of questions and you are encouraged to express your opinions and views freely. During the interview, I will ask you if I have questions about what you have said or if I need you to clarify a topic or concept.

感谢您愿意参加并在此接受采访。我叫 XXX，是 XXX 大学的博士。目前，我正在开展一个研究项目，主要收集在使用定制家具时的用户体验资料。在整个访谈中，我会问您一系列问题，我们鼓励您自由表达您的意见和观点。在访谈过程中，如果我对你所说的内容有疑问或需要您澄清一个主题或概念，我会向您询问。

Researcher

What is the square footage of your house?

你的房子的面积是多少?

Informant 015

100m2

Researcher

How big is your family? What's the family structure like?

您的家庭人数？家庭结构是什么样的？

Informant 015

3。 我、爸爸、妈妈

3. Me, dad, mom.

Researcher

What is the style of furniture in the home?

家中家具是什么样式的?

Informant 015

Modern simplicity

现代简约

Researcher

Where is the custom furniture placed? What are the main cabinets?

定制家具放置在哪里?主要是哪些柜体?

Informant 015

Bedroom, dining room. Wardrobe, kitchen cabinet.

卧室、餐厅。衣柜、橱柜。

Researcher

What is your custom furniture style? Is it consistent with the home decor?

您家定制家具风格是什么样?和家中装修风格一致吗?

Informant 015

Modern simplicity. Consistency.

现代简约。一致。

Researcher

How much do you spend on custom furniture?

你花多少钱在定制家具上?

Informant 015

5-10 thousand.

5-10 万。

Researcher

What is your understanding of custom furniture?

您对定制家具的理解是什么？

Informant 015

I think that customized furniture can be designed and manufactured according to the specific requirements of customers, starting from size, material, color, style, function, etc., to meet the actual needs and personalized requirements of customers. Custom furniture is a tailor-made service that is more unique than the mass production of standard furniture.

我觉得定制家具可以按照客户的具体要求进行设计和制造，从尺寸、材料、颜色、样式、功能等方面入手，以满足客户的实际需求和个性化要求。定制家具是一项量身定做的服务，跟标准家具的批量生产相比，定制家具更为独一无二。

Researcher

What do you know about custom furniture brand channels? (advertising or otherwise)

您了解定制家具品牌渠道是什么?

Informant 015

Advertising. The brand will promote itself through various media such as television, radio, newspapers, and the internet to increase its brand awareness and reputation.

广告宣传。品牌会通过电视、广播、报刊、网络等媒介进行广告宣传，以提高品牌知名度和美誉度。

Researcher

How do you know about custom furniture?

您是怎么了解定制家具相关内容?

Informant 015

Browsing relevant websites and forums on the internet to understand the trend, design concepts, production techniques of custom furniture.

在互联网上浏览相关网站和论坛，了解定制家具的潮流趋势、设计理念、制作工艺等。

Researcher

What was your initial impression of the brand you chose? What was the initial understanding?

您对您选择的品牌最初印象是什么？最初的理解是什么？

Informant 015

Appearance.

外观。

Researcher

Why do you choose this brand of custom furniture?

您选择该品牌的定制家具的原因是什么？

Informant 015

Good-looking.

好看。

Researcher

What do you think are the advantages of custom furniture over finished furniture?

您认为相比成品家具，定制家具的优势是什么?

Informant 015

Personalization: Customized furniture can be designed and manufactured according to the customer's needs and requirements, meeting their personalized demands in terms of size, color,

material, style, function and more. This is more in line with the customer's aesthetic and usage needs.

个性化：定制家具能够根据客户的需求和要求进行设计和制造，从尺寸、颜色、材料、样式、功能等方面来满足客户的个性化需求，更符合客户的审美和使用需求。

Researcher

What do you think you should pay attention to when choosing custom furniture?

您觉得在选择定制家具时应该注意什么问题?

Informant 015

Functional requirements of furniture: When choosing customized furniture, the first step is to select the functions and types of furniture based on your own household needs and lifestyle habits, including storage function, usage location, color matching, etc.

家具的功能需求：在选择定制家具时，首先要根据自己的家庭需求和生活习惯来选择家具的功能和类型，包括储物功能、使用场所、颜色搭配等。

Researcher

How often do you use cabinets, closets, and other custom furniture?

您使用橱柜、衣柜、和其他定制的家具的频率是如何的?

Informant 015

Used every day.

每天都用。

Researcher

Do current custom furniture products meet your needs with tactile details?

当前定制家具产品触觉细节满足您的需求吗?

Informant 015

符合

Yes

Researcher

How do you open and close your custom furniture?

您家定制家具开关门方式是什么样的?

Informant 015

Folding door: A folding door refers to a door that folds and opens along the vertical direction of the door panel. It can also save space compared to traditional doors, making it suitable for smaller spaces.

折叠门：折叠门是指门板沿着竖直方向折叠开关的门，同样可以省去传统门的开门占用空间，适合空间较小的场合。

Researcher

Will you share your successful decorating experience with others?

您会与别人分享您的装修成功经验吗?

Informant 015

Yes

会

Researcher

What do you think are the disadvantages of current custom furniture?

您觉得当前的定制家具的缺点是什么?

Informant 015

Higher price: The design and production of custom furniture require more time and effort, so the price is usually higher than ready-made furniture.

价格较高：定制家具的设计和生产需要更多的时间和精力，因此价格通常比现成家具更高。

Researcher

What other features do you think can be added to custom furniture?

您觉得定制家具可以添加什么其他功能？

Informant 015

Smart features: In customized furniture such as TV cabinets and desks, smart control systems can be added, such as voice control and remote control, to improve convenience and comfort of use.

智能功能：在定制电视柜、书桌等家具中，可以添加智能控制系统，例如语音控制、遥控器控制等，提高使用的便利性和舒适度。

Researcher

What aspects of custom furniture can provide more possibilities for users?

定制家具的哪些方面可以为用户提供更多的可能性?

Informant 015

Design: Customized furniture can be uniquely designed according to the personalized needs and preferences of users. Users can choose different materials, colors, styles, etc. based on their own preferences, space characteristics and functional requirements to create distinctive furniture.

设计：定制家具可以根据用户的个性化需求和喜好，进行独特的设计。用户可以根据自己的喜好、空间的特点和功能需求，选择不同的材料、色彩和款式等，以打造出与众不同的家具。

Researcher

Okay, that's the end of this interview, thank you for participating.

好的，本次的访谈到此结束，感谢您的参与。
