# Supplementary material for: Building a 4E interview-grounded theory model: A case study of demand factors for customized furniture
Source: PLoS One. 2023 Apr 27;18(4):e0282956. doi: 10.1371/journal.pone.0282956 (PMC10138260; doi:10.1371/journal.pone.0282956)
Supplement: S1 File — (ZIP) [file pone.0282956.s001.zip › transcript/transcript 016.pdf]

Informant : 016

Please note that the original transcript is in Simplified Chinese. The English translation is for internal communication among the author of this research, and it is not proofread. Potential linguistic errors may exist in the English translation.

Thank you for your willingness to participate and be interviewed here. My name is XXX, and I'm a PhD in the XXX University. Currently, I am working on a research project that focuses on collecting information about user demand when purchasing and using customized furniture. Throughout the interview, I will ask you a series of questions and you are encouraged to express your opinions and views freely. During the interview, I will ask you if I have questions about what you have said or if I need you to clarify a topic or concept.

感谢您愿意参加并在此接受采访。我叫 XXX，是 XXX 大学的博士。目前，我正在开展一个研究项目，主要收集在使用定制家具时的用户体验资料。在整个访谈中，我会问您一系列问题，我们鼓励您自由表达您的意见和观点。在访谈过程中，如果我对你所说的内容有疑问或需要您澄清一个主题或概念，我会向您询问。

Researcher

What is the square footage of your house?

你的房子的面积是多少?

Informant 016

139 平方米。

139 ㎡

Researcher

How big is your family? What's the family structure like?

您的家庭人数？家庭结构是什么样的？

Informant 016

3 人，家里住着我们夫妻俩和一个孩子。

3 people, husband and wife living in the family and a child.

Researcher

What is the style of furniture in the home?

家中家具是什么样式的？

Informant 016

The furniture in the home adopted modern and contracted style on the whole, the whole is concise and generous, and also can contain the small ornaments of a few exotic culture among them, add the interest in the home.

家中家具整体上采用了现代简约风格，整体简洁大方，其中也会包含一些异域文化的小摆件，增添家中的趣味。

Researcher

Where is the custom furniture placed? What are the main cabinets?

定制家具放置在哪里？主要是哪些柜体？

Informant 016

Customized furniture is mainly placed in the primary and secondary bedroom and the living room, mainly including the wardrobe of the bedroom and the dining side cabinet in the living room.

定制的家具主要放置在主次卧和客厅，主要包括卧室的衣柜以及客厅的餐边柜等。

Researcher

What is your custom furniture style? Is it consistent with the home decor?

您家定制家具风格是什么样？和家中装修风格一致吗？

Informant 016

For harmony, unity, the customized furniture style still adopts the modern and contracted style, consistent with the home decoration style, making the general style of the whole home is consistent.

为了和谐性、统一性，所定制的家具风格仍然采用了现代简约风格，与家中装修风格一致，使得整个家的大体风格保持一致。

Researcher

How much do you spend on custom furniture?

你花多少钱在定制家具上?

Informant 016

The two wardrobes in the two bedrooms plus the dining side cabinets in the living room cost about 8,000 yuan.

两间卧室中的两个衣柜加上客厅的餐边柜一共花费了 8000 人民币左右。

Researcher

What is your understanding of custom furniture?

您对定制家具的理解是什么？

Informant 016

It is probably designed to design customized personal furniture according to personal preferences, at the same time can adapt to the space layout and ensure the harmony and unity of the environment, in the case of ensuring that the style does not violate, and can have their own unique ideas and design style.

大概就是根据个人喜好请人设计定制个人专属的家具，同时可以适应空间布局以及保证环境的和谐统一，在保证风格不违和的情况下，又能有自己独特的想法和设计风格。

Researcher

What do you know about custom furniture brand channels?

您了解定制家具品牌渠道是什么?

Informant 016

The channel to understand customized furniture is through online e-commerce platforms such as Taobao, Jingdong, and watch through some live broadcasts. The other is to choose the customized furniture they have bought for good quality through the recommendation of elders.

了解定制家具的渠道一是通过淘宝，京东等线上电商平台，并通过一些直播进行观看，二是通过长辈推荐，选择他们曾经购买过的，质量等方面得到好评的定制家具。

Researcher

How do you know about custom furniture?

您是怎么了解定制家具相关内容?

Informant 016

I have always heard about the publicity of customized furniture. In the decoration, after repeated deliberation and inquiry of information, I choose field investigation, to the relevant brand entity stores to consult relevant knowledge, how to communicate with designers and other issues.

一直都有听过关于定制家具的宣传，在装修时，经过反复推敲、查询资料之后选择实地考察，到相关品牌实体店咨询相关知识、价格如何与设计师沟通等问题。

Researcher

What was your initial impression of the brand you chose? What was the initial understanding?

您对您选择的品牌最初印象是什么? 最初的理解是什么？

Informant 016

Finally, I chose SOGAL as the purchasing unit. My initial impression of SOGAL is that the scene layout in SOGAL stores is very good, which can easily experience different decoration furniture schemes, and can feel the appearance after decoration effectively, with certain authenticity and diversity.
最终选择索菲亚为购买单位，我对索菲亚的最初印象是索菲亚的卖场中的场景化布置非常好，可以非常便捷的体验各个不同的装修家具方案，能够实体感受装修后的面貌，具有一定的真实性、多样性。

Researcher
Why do you choose this brand of custom furniture?
您选择该品牌的定制家具的原因是什么？

Informant 016
A large part of SOGAL's customized furniture is due to the recommendation of elders and personal visit to SOGAL stores, I heard about SOGAL before, and was attracted by its concept and style.
选择索菲亚的定制家具很大一部分原因是由于家里长辈推荐，并且有到索菲亚卖场进行参观选择，切身实地考察；另外之前对索菲亚也略有耳闻，被它的理念、风格所吸引。

Researcher
What do you think are the advantages of custom furniture over finished furniture?
您认为相比成品家具，定制家具的优势是什么?

Informant 016
Customized furniture can better fit the layout of the home, there will be no wardrobe wall and other problems, into the environment; the style can also be very good fit, do not need to do; at the same time, according to self needs, do not waste every space, to achieve the best utilization.

定制家具可以更好的贴合家中的布局，不会有衣柜不贴墙等问题出现，融于环境；风格上也能够非常好的契合，并不需要将就；同时能够根据自我需要进行功能分区，不浪费每一个空间，达到最好的利用率。

Researcher

What do you think you should pay attention to when choosing custom furniture?

您觉得在选择定制家具时应该注意什么问题？

Informant 016

First of all, we should communicate with the designer, listen to the designer's ability to control the spatial layout, but also take the initiative to put forward our own needs to achieve satisfactory results. Secondly, pay attention to the choice of materials, choose environmentally friendly and harmless materials to ensure the safe and harmless indoor environment; finally, choose the right enterprise for customization

首先应该多和设计师沟通磨合，要听从设计师对空间布局的把控能力，但也要主动提出自己的需求，从而达到双方满意的结果，其次注意材料的选择，选择环保、无害的材料，确保室内环境的安全无害；最后要选择合适的企业进行定制。

Researcher

How often do you use cabinets, closets, and other custom furniture?

您使用橱柜、衣柜、和其他定制的家具的频率是如何的？

Informant 016

Because the furniture is more durable, we rarely consider customized new furniture, usually when it is damaged, or when the home needs to be renovated.

由于家具都比较耐用所以很少会考虑定制新的家具，一般是在其有所损坏，或者家中需要重新装修时才会考虑更换新的定制家具。

Researcher

Does the appearance of current custom furniture products meet your needs?

当前定制家具产品外观满足您的需求吗?

Informant 016

I am more satisfied with the appearance of the current customized furniture products, more selective, diversified style, fit the modern development, with a certain sense of science and technology, convenient and beautiful.

我对当前的定制家具产品外观较为满足，可选择性大，风格多样化，贴合现代发展，具有一定的科技感，便捷、美观。

Researcher

Do current custom furniture products meet your needs with tactile details?

当前定制家具产品触觉细节满足您的需求吗?

Informant 016

In my opinion, the tactile details of customized furniture products are still good. The diversification of materials makes the furniture that can feel different touches. In addition, the existing customized furniture pays special attention to detail in the design, so it can also enjoy the touch.

我觉得定制家具产品的触觉细节还是不错的，材料的多样化使得可以感受不同触感的家具，另外现有的定制家具在设计上尤为注重细节，因此触觉上也能有所享受。

Researcher

Does the current custom furniture fit your functional needs? Which need is not being met?

当前的定制家具是否符合您对产品功能的需求？哪一个需求没有得到满足？

Informant 016

The current customized furniture is generally in line with my demand for product functions, but it is a pity that the master bedroom wardrobe can not be used to move

the door because the bed was arranged before. But overall, it met expectations and was worth it.
当前定制的家具大体上是符合我对产品功能的需求的，但是主卧衣柜因为之前布置好的床不能装推拉式的门只能用移门比较可惜。但总的来说，达到了预期，非常值得。

Researcher
Does the current custom furniture meet your need for product audibility or smell?
当前定制家具是否符合您对产品可听性或气味的需求？

Informant 016
The current wardrobe and meal side cabinet do not have what smell, will not appear pungent smell, environmental protection can reach the standard, but the wardrobe of the door to push the sound is not very comfortable, sometimes there will be a harsh sound.
当前的衣柜和餐边柜都没有什么气味，不会出现刺激性气味，环保方面能够达标，但是衣柜的移门推动的声音并不是很舒服，有时会有刺耳的声音。

Researcher
How do you open and close your custom furniture? How do you like to open and close the door?
您家定制家具开关门方式是什么样的？您喜欢哪种开关门方式？

Informant 016
At present, the custom furniture in the home is moving the door. But I prefer the push-pull door, which can be fully opened, and can directly see the items inside the whole cabinet visually, which is more convenient.
目前家中的定制家具为移门。但我更喜欢推拉式的门，可以全打开，能够视觉上直接看到整个柜子内部的物品，更加方便。

Researcher

Will you share your successful decorating experience with others?

您会与别人分享您的装修成功经验吗?

Informant 016

If your relatives and friends want to decorate, they should share their decoration experience to help them quickly choose the right furniture products.

如果亲戚朋友要装修的话应该会分享装修经验，帮助他们能够快速选择合适的家具产品

Researcher

What do you think are the disadvantages of current custom furniture?

您觉得当前的定制家具的缺点是什么?

Informant 016

I think the custom cycle of furniture is a little long, so it is not convenient to get the product in time.

我觉得家具的定制周期有些长，不方便及时拿到产品。

Researcher

What other features do you think can be added to custom furniture?

您觉得定制家具可以添加什么其他功能？

Informant 016

Customized furniture can be added to some products with furniture, such as the cabinet can have shadow induction lights, and some tableware in the cabinet can be recommended, which can enrich the versatility of furniture products.

定制家具可以添加一些与家具配套的产品，例如柜子可以有影藏的感应灯，餐边柜可以推荐一些餐具等，能够丰富家具产品的多功能性。

Researcher

What aspects of custom furniture can provide more possibilities for users?

定制家具的哪些方面可以为用户提供更多的可能性?

Informant 016

The selectivity is stronger, in the selection of furniture, customized furniture in the selection, application and applicability and other aspects, have a strong performance, whether the material, color and style have multiple choices.

选择性更强，在进行家具选择时，定制家具在选择、应用以及适用性等多个方面，都有着很强的表现，无论是材料、颜色和款式等都有了多重选择。
