# Supplementary material for: Building a 4E interview-grounded theory model: A case study of demand factors for customized furniture
Source: PLoS One. 2023 Apr 27;18(4):e0282956. doi: 10.1371/journal.pone.0282956 (PMC10138260; doi:10.1371/journal.pone.0282956)
Supplement: S1 File — (ZIP) [file pone.0282956.s001.zip › transcript/transcript 017.pdf]

Informant : 017

Please note that the original transcript is in Simplified Chinese. The English translation is for internal communication among the author of this research, and it is not proofread. Potential linguistic errors may exist in the English translation.

Thank you for your willingness to participate and be interviewed here. My name is XXX, and I’m a PhD in the XXX University. Currently, I am working on a research project that focuses on collecting information about user demand when purchasing and using customized furniture. Throughout the interview, I will ask you a series of questions and you are encouraged to express your opinions and views freely. During the interview, I will ask you if I have questions about what you have said or if I need you to clarify a topic or concept.

感谢您愿意参加并在此接受采访。我叫 XXX，是 XXX 大学的博士。目前，我正在开展一个研究项目，主要收集在使用定制家具时的用户体验资料。在整个访谈中，我会问您一系列问题，我们鼓励您自由表达您的意见和观点。在访谈过程中，如果我对你所说的内容有疑问或需要您澄清一个主题或概念，我会向您询问。

Researcher

What is the square footage of your house?

你的房子的面积是多少?

Informant 017

130 平方

130 square

Researcher

How big is your family? What's the family structure like?

您的家庭人数？家庭结构是什么样的？

Informant 017

Three people, my father, mother and me

3 人，爸爸妈妈和我

Researcher

What is the style of furniture in the home?

家中家具是什么样式的？

Informant 017

Modern simple, white as the main theme, because my parents and I like a bit of modern style, and we think the more simple furniture is more durable.

现代简约，以白色为主旋律的，因为父母和我都喜欢现代一点的风格，而且我们都认为越简约的家具越耐看。

Researcher

Where is the custom furniture placed? What are the main cabinets?

定制家具放置在哪里？主要是哪些柜体？

Informant 017

It is mainly placed in the bedroom, living room and dining room. The main cabinets are the wardrobe in the bedroom, the wine cabinet in the guest dining room and the electrical cabinet in the living room.

主要放置在卧室、客厅和餐厅，主要柜体为卧室里的衣柜，客餐厅酒柜，以及客厅的电器柜。

Researcher

What is your custom furniture style? Is it consistent with the home decor?

您家定制家具风格是什么样？和家中装修风格一致吗？

Informant 017

The custom furniture is also modern and simple, in line with the home decor.

定制的家具也是现代简约风格的，和家中装修风格一致。

Researcher

How much do you spend on custom furniture?

你花多少钱在定制家具上?

Informant 017

3-5 million

3-5 万

Researcher

What is your understanding of custom furniture?

您对定制家具的理解是什么？

Informant 017

Customized home can be customized according to the style and style of consumers need to make personalized products, which conforms to the pursuit of personalized consumption needs of young people. In addition, customized furniture can be customized according to the specific household type personalized furniture, effective use of every inch of space, increase the practicality of furniture, which is also an important factor for many consumers to choose customized furniture, customized furniture makes furniture more practical and reasonable.

定制家居可以根据消费者需要的款式和风格做出个性化产品，这符合年轻人追求个性化消费需求。另外，定制家居可以根据具体户型定制个性化家具，有效利用每一寸空间，增加了家具的实用性，这也是众多消费者选择定制家居的重要因素，定制家具让家具更具有实用性，合理性。

Researcher

What do you know about custom furniture brand channels? (advertising or otherwise)

您了解定制家具品牌渠道是什么?（广告或其他）

Informant 017

Advertising and word-of-mouth, usually through advertising to understand some brands and types, also heard from friends and relatives around what brand furniture is good, will pay more attention to this brand.

广告和相传口碑，平时通过广告来了解到一些品牌和种类，也从周围朋友亲戚等听说什么牌子的家具好用，也会多注意这一品牌。

Researcher

How do you know about custom furniture?

您是怎么了解定制家具相关内容?

Informant 017

Sales, Internet search, offline experience library contact

销售，网络搜索，线下体验馆接触

Researcher

What was your initial impression of the brand you chose? What was the initial understanding?

您对您选择的品牌最初印象是什么？最初的理解是什么？

Informant 017

When I choose a brand, I will look at their design and sense of shape, tone and material at the beginning. If they do well in these aspects, they will leave a good impression on me. Second is the big brand, because the big brand after-sales and service in place, purchase is guaranteed.

我选择品牌的时候，一开始会看他们的设计和造型感，色调和材质，这些方面做得好的话会给我留下很好的印象。其次就是大品牌，因为大品牌售后和服务到位，购买的话也有保障。

Researcher

Why do you choose this brand of custom furniture?

您选择该品牌的定制家具的原因是什么？

Informant 017

The brand I chose matches the market share, price and appearance of the material, and the design style is more in line with my parents' and my aesthetic taste. In addition, the after-sales service and service are also in place.

我选择的品牌市场占有率，价位与材质外观造型相匹配，而且设计风格比较符合父母和我的审美，另外售后和服务也比较到位。

Researcher

What do you think are the advantages of custom furniture over finished furniture?

您认为相比成品家具，定制家具的优势是什么?

Informant 017

I think customized furniture can reasonably adapt to the overall interior layout environment style, can give reasonable products according to user needs, and does not waste space, more reasonable and convenient to use.

我认为定制家具可以合理的与整体室内布局环境格调相适应，能根据用户需求给出合理的产品，而且不浪费空间，使用起来更加合理、便捷。

Researcher

What do you think you should pay attention to when choosing custom furniture?

您觉得在选择定制家具时应该注意什么问题?

Informant 017

I think in the custom furniture should first pay attention to the environmental protection of the plate, after all, you and your family to deal with furniture every day, safety is very important. The second is the permanent use of accessories, because customized furniture generally will not change, unlike other furniture dislike can be replaced immediately. Finally, harmony with the rest of the home. Only when the whole is harmonious, will the home look harmonious, neat and

comfortable.
我觉得在定制家具时首先应该注意板材的环保性，毕竟自己和家人要每天与家具打交道，安全很重要。其次是配件使用的长久性，因为定制家具一般不会更改，不像别的家具一样不喜欢可以立马更换。最后是与家中其他部分的协调性，只有整体协调，家里才会看起来协调，整洁，令人舒适。

Researcher
How often do you use cabinets, closets, and other custom furniture?
您使用橱柜、衣柜、和其他定制的家具的频率是如何的?

Informant 017
It's used almost every day. It's basic furniture.
基本上每一天都会使用，这些都是最基本的家具。

Researcher
Does the appearance of current custom furniture products meet your needs?
当前定制家具产品外观满足您的需求吗?

Informant 017
Basically satisfied, all belong to the simple modern style, the family is very satisfied. Because custom furniture can use a variety of materials and colors. We can choose a variety of materials such as wood, metal, glass, and plastic, and can also choose a variety of colors and textures to meet their individual needs.
基本满足，都属于简约现代风格， 家人都很满意。定制家具可以使用各种材料和颜色。我们可以选择木材、金属、玻璃、塑料等多种材料，还可以选择各种颜色和纹理，以满足自己的个性化需求。

Researcher
Do current custom furniture products meet your needs with tactile details?

当前定制家具产品触觉细节满足您的需求吗?

Informant 017

Basically satisfied, furniture material is also a part of our choice at that time

基本满足，家具的材质也是我们当时选择的一部分

Researcher

Does the current custom furniture fit your functional needs? Which need is not being met?

当前的定制家具是否符合您对产品功能的需求？哪一个需求没有得到满足？

Informant 017

The current furniture basically meets my functional needs. If there is anything not satisfied, should be the space structure also needs to be more reasonable life, sometimes feel that there is still some space can be used. There is also the need for colors to blend more into the home.

当前的家具基本符合我对产品功能的需求。如果说还有什么不满足的，应该是空间结构还需要更加合理生活化，有时候觉得还有一些空间可以利用。还有就是颜色需要更融入家中环境。

Researcher

Does the current custom furniture meet your need for product audibility or smell?

当前定制家具是否符合您对产品可听性或气味的需求？

Informant 017

The smell of the plate is still not satisfied

板材气味还是不太满意

Researcher

How do you open and close your custom furniture?

您家定制家具开关门方式是什么样的？

Informant 017

The wardrobe in our bedroom is automatically rebounding closed, press slowly to open. The liquor cabinet and TV cabinet are push-pull.

我们家卧室的衣柜是自动回弹关闭，按动缓慢弹开。酒柜和电视柜是推拉的。

Researcher

How do you like to open and close the door?

您喜欢哪种开关门方式？

Informant 017

Personally, I prefer the automatic rebound opening and closing mode.

个人比较喜欢自动回弹的开关门方式。

Researcher

Will you share your successful decorating experience with others?

您会与别人分享您的装修成功经验吗?

Informant 017

Yes, because I want others to learn from my own success

会的，因为自己成功的地方也想让别人借鉴一下

Researcher

What do you think are the disadvantages of current custom furniture?

您觉得当前的定制家具的缺点是什么?

Informant 017

The price is relatively high, because it is customized to each person's different space, so the price is not very low. And the need for users to have a certain planning ability for space, to have a certain understanding of all kinds of plates and hardware, the structure and tone of the need for a certain knowledge reserve, so as to customize the furniture suitable for home.

价格相对较高，因为是根据每个人不同的空间定制的，所以价格都不是很低。而且需要用户对空间有一定的规划能力，要对各种板材各种五金件有一定的了解，对结构和色调需要一定的知识储备，这样才能定制到适合家里的家具。

Researcher

What other features do you think can be added to custom furniture?

您觉得定制家具可以添加什么其他功能？

Informant 017

我觉得还可以添加智能化，人性化，更符合用户的生活行为习惯，改善空间架构，板材的可移动性等功能。

Intelligent, humanized, more in line with the user 's living habits, improve the spatial structure, sheet mobility

Researcher

What aspects of custom furniture can provide more possibilities for users?

定制家具的哪些方面可以为用户提供更多的可能性?

Informant 017

Function and style : Customized furniture can meet the functional and style needs of users. Users can choose a variety of different functions, such as storage space, hidden storage, adjustable seats and so on. At the same time, users can also choose different styles, such as modern, traditional, retro, industrial and so on, to meet their individual needs.

Customized needs: If users have special needs, such as disabled appliances, children's furniture, particularly high or low seats, etc., custom furniture can meet these needs.

功能和风格：定制家具可以满足用户的功能和风格需求。用户可以选择各种不同的功能，如存储空间、隐藏式储物、可调节的座椅等等。同时，用户还可以选择不同的风格，如现代、传统、复古、工业等等，以适应自己的个性化需求。

定制需求：如果用户有特殊的需求，如残疾人用具、儿童家具、特别高或低的座位等等，

定制家具可以满足这些需求。

Researcher

We were inspired by what you mentioned about meeting the needs of special populations. This concludes this interview, thank you for participating.

您提到的能到满足特殊人群的需求这一点让我们很受启发。本次访谈到此结束，谢谢您的参与。
