# Supplementary material for: Building a 4E interview-grounded theory model: A case study of demand factors for customized furniture
Source: PLoS One. 2023 Apr 27;18(4):e0282956. doi: 10.1371/journal.pone.0282956 (PMC10138260; doi:10.1371/journal.pone.0282956)
Supplement: S1 File — (ZIP) [file pone.0282956.s001.zip › transcript/transcript 018.pdf]

Informant : 018

Please note that the original transcript is in Simplified Chinese. The English translation is for internal communication among the author of this research, and it is not proofread. Potential linguistic errors may exist in the English translation.

Thank you for your willingness to participate and be interviewed here. My name is XXX, and I’m a PhD in the XXX University. Currently, I am working on a research project that focuses on collecting information about user demand when purchasing and using customized furniture. Throughout the interview, I will ask you a series of questions and you are encouraged to express your opinions and views freely. During the interview, I will ask you if I have questions about what you have said or if I need you to clarify a topic or concept.

感谢您愿意参加并在此接受采访。我叫 XXX，是 XXX 大学的博士。目前，我正在开展一个研究项目，主要收集在使用定制家具时的用户体验资料。在整个访谈中，我会问您一系列问题，我们鼓励您自由表达您的意见和观点。在访谈过程中，如果我对你所说的内容有疑问或需要您澄清一个主题或概念，我会向您询问。

Researcher

What is the square footage of your house?

你的房子的面积是多少?

Informant 018

240m^2.

Researcher

How big is your family? What's the family structure like?

您的家庭人数？家庭结构是什么样的？

Informant 018

三个人，我和父母。

Parents and children

Researcher

What is the style of furniture in the home?

家中家具是什么样式的？

Informant 018

Home decoration is more traditional, it is the antique wooden furniture.

家里装修比较传统，是古色古香的木制家具。

Researcher

Where is the custom furniture placed? What are the main cabinets?

定制家具放置在哪里？主要是哪些柜体？

Informant 018

Basically be bedroom, kitchen, wooden ark body is in the majority

主要是卧室、厨房，木制柜体居多

Researcher

What is your custom furniture style? Is it consistent with the home decor?

您家定制家具风格是什么样？和家中装修风格一致吗？

Informant 018

Mix and match style, because I and parents like the style is not the same, but we do not interfere with each other, their own space can be decorated into their own like.

混搭风格，因为我和父母喜欢的风格不一样，但是我们相互之间按互不干涉，自己的空间可以装修成自己喜欢的样子。

Researcher

How much do you spend on custom furniture?

你花多少钱在定制家具上?

Informant 018

I don't know. I bought it many years ago when I was young.

我不太清楚，是很多年前我还比较小的时候买的了。

Researcher

What is your understanding of custom furniture?

您对定制家具的理解是什么？

Informant 018

Build on the basis of their own preferences should also take into account the actual situation of the house.

建立在自己喜好的基础上也要考虑到房屋的实际情况。

Researcher

What do you know about custom furniture brand channels? (advertising or otherwise)

您了解定制家具品牌渠道是什么?（广告或其他）

Informant 018

Through some social network platforms, media channels such as Xiaohongshu, Zhihu, Taobao, TikTok, etc., which young people like to brush, I will also listen to friends introduce some buying experience.

会通过一些社交网络平台，媒体渠道例如像年轻人都喜欢刷的小红书、知乎、淘宝、抖音等，也会听朋友介绍一些购买经验。

Researcher

How do you know about custom furniture?

您是怎么了解定制家具相关内容?

Informant 018

There are many on the network, such as Baidu, Zhihu, Xiaohongshu, can also understand some more authoritative details through the relevant institutions, personnel, or can also field investigation, go to the furniture city, furniture shopping malls.

在网络上有很多，例如百度、知乎、小红书，还可以通过相关机构、人员了解一些更加权威的细节，或者也可以实地考察，去家具城、家具商场逛逛。

Researcher

What was your initial impression of the brand you chose? What was the initial understanding?

您对您选择的品牌最初印象是什么? 最初的理解是什么？

Informant 018

I think the brand that can move me is high quality, high cost-effective, I want to use it well, but also in the economic range I can afford.

我觉得能打动我的品牌是高品质、高性价比的，机要用得好，也要在我能承受的经济范围内。

Researcher

Why do you choose this brand of custom furniture?

您选择该品牌的定制家具的原因是什么？

Informant 018

There is a very good relationship friend recommended, said the last decoration when the choice of this experience is very good, I was recommended by him.

有个关系非常好的朋友推荐的，说上次装修的时候选择这家的体验感非常好，我

被他推荐过来了。

Researcher

What do you think are the advantages of custom furniture over finished furniture?

您认为相比成品家具，定制家具的优势是什么?

Informant 018

Better fitting use of building structure; add unique and individual design points; increase individual required structure

更好的贴合利用房屋结构；增加独特、个性的设计点；增加个人所需结构

Researcher

What do you think you should pay attention to when choosing custom furniture?

您觉得在选择定制家具时应该注意什么问题？

Informant 018

Attention should be paid to four aspects, mainly: deposit and deposit; cabinet body plate selection; cabinet body sealing edge; hardware accessories. These four aspects are concerned about and can be solved if it will be very successful, but often still have to make some compromise, customized furniture, there is no way to completely customized, or to see the actual situation.

要注意 4 个方面，主要是：订金与定金；柜体板材选择；柜体封边；五金配件。这四个方面都关注到并且都能解决的话就会非常成功，但是往往还是要做出一些妥协的，定制家具也没有办法完全定制，还是眼看实际情况。

Researcher

How often do you use cabinets, closets, and other custom furniture?

您使用橱柜、衣柜、和其他定制的家具的频率是如何的？

Informant 018

three to four times a day;
每天三到四次。

Researcher
Does the appearance of current custom furniture products meet your needs?
当前定制家具产品外观满足您的需求吗?

Informant 018
Not satisfied, customization is not completely customized, there will be some restrictions, I hope that one day I can be more personalized customization.
不满足，定制并不是完全定制，会有一些限制，希望有一天可以更加个性化地定制。

Researcher
Do current custom furniture products meet your needs with tactile details?
当前定制家具产品触觉细节满足您的需求吗?

Informant 018
Generally, the tactile aspect may mainly depend on the quality and type of the plate, at the beginning of the custom furniture ignored the texture of this point, although not bad, but in the touch is ordinary.
一般，触觉方面主要可能取决于板材的品质和种类，当初定制家具的时候忽略了质感这一点，虽然不差，但是在触感上就是平平无奇吧。

Researcher
Does the current custom furniture fit your functional needs? Which need is not being met?
当前的定制家具是否符合您对产品功能的需求？哪一个需求没有得到满足？

Informant 018

No, there are two main aspects: storage space and structure. Because there are more pieces and debris in my home, not easy to classify, good storage space is particularly important, I think the structure is more ordinary.
没有，主要是两个方面：储存空间和结构。因为我家里的零碎杂物比较多，不好归类，好的存储空间格外重要，结构的话我觉得是比较普通。

Researcher
Does the current custom furniture meet your need for product audibility or smell?
当前定制家具是否符合您对产品可听性或气味的需求？

Informant 018
Yes, there is a faint smell of wood.
符合，有一种淡淡的木头香味。

Researcher
How do you open and close your custom furniture? How do you like to open and close the door?
您家定制家具开关门方式是什么样的？您喜欢哪种开关门方式？

Informant 018
Yes, there is a faint smell of wood.
推拉式和触碰式都有。

Researcher
Will you share your successful decorating experience with others?
您会与别人分享您的装修成功经验吗?

Informant 018
Yes, I will post my experience with my friends and on social media platforms.

会，会跟朋友还有在社交平台发布一下经验。

Researcher

What do you think are the disadvantages of current custom furniture?

您觉得当前的定制家具的缺点是什么?

Informant 018

Generally, customized furniture has a long cycle, so the finished product cannot be seen in time, so it must be booked in advance. The production cycle is long. If you want to use furniture, the efficiency of finished furniture is relatively high.

一般定制家具周期长，无法及时看见成品，须提前预定，生产周期久，如果着急想要使用家具的话还是成品家具效率比较高。

Researcher

What other features do you think can be added to custom furniture?

您觉得定制家具可以添加什么其他功能？

Informant 018

Intelligent, a lot of furniture is designed on appearance and style, but the present era adds some intelligent elements can let a person shine at the moment.

智能化，很多家具都是在外形和风格上进行设计，但是现在的时代多加一些智能化的元素会让人眼前一亮。

Researcher

What aspects of custom furniture can provide more possibilities for users?

定制家具的哪些方面可以为用户提供更多的可能性?

Informant 018

Can expand the whole customized category; increase the customer unit price, but also can be linked with different categories of products.

可以扩充整家定制品类；提升客单价，还可以与不同品类产品的联动。

Informant 018

OK, this is the end of this interview, I wish you a happy life.

好滴，本次访谈到此就结束了，祝您生活愉快
