# Supplementary material for: Building a 4E interview-grounded theory model: A case study of demand factors for customized furniture
Source: PLoS One. 2023 Apr 27;18(4):e0282956. doi: 10.1371/journal.pone.0282956 (PMC10138260; doi:10.1371/journal.pone.0282956)
Supplement: S1 File — (ZIP) [file pone.0282956.s001.zip › transcript/transcript 019.pdf]

Informant : 019

Please note that the original transcript is in Simplified Chinese. The English translation is for internal communication among the author of this research, and it is not proofread. Potential linguistic errors may exist in the English translation.

Thank you for your willingness to participate and be interviewed here. My name is XXX, and I'm a PhD in the XXX University. Currently, I am working on a research project that focuses on collecting information about user demand when purchasing and using customized furniture. Throughout the interview, I will ask you a series of questions and you are encouraged to express your opinions and views freely. During the interview, I will ask you if I have questions about what you have said or if I need you to clarify a topic or concept.

感谢您愿意参加并在此接受采访。我叫 XXX，是 XXX 大学的博士。目前，我正在开展一个研究项目，主要收集在使用定制家具时的用户体验资料。在整个访谈中，我会问您一系列问题，我们鼓励您自由表达您的意见和观点。在访谈过程中，如果我对你所说的内容有疑问或需要您澄清一个主题或概念，我会向您询问。

Researcher

What is the square footage of your house?

你的房子的面积是多少?

Informant 019

100 Square meters

100 平方米

Researcher

How big is your family? What's the family structure like?

您的家庭人数？家庭结构是什么样的？

Informant 019

Four people

4 人

Me, my parents, and my grandparents

我、父母、弟弟

Researcher

What is the style of furniture in the home?

家中家具是什么样式的？

Informant 019

Modern contracted

典雅简约

Researcher

Where is the custom furniture placed? What are the main cabinets?

定制家具放置在哪里？主要是哪些柜体？

Informant 019

Bedroom, living room

卧室、客厅

Closets, shoe cabinets

衣橱、鞋柜、茶几

Researcher

What is your custom furniture style? Is it consistent with the home decor?

您家定制家具风格是什么样？和家中装修风格一致吗？

Informant 019

Modern contracted. And the home decoration style is consistent

雅致简约和家中装修风格有一点偏差，整体较为和谐，并无突兀之处。

Researcher

How much do you spend on custom furniture?

你花多少钱在定制家具上?

Informant 019

Ten thousand yuan to fifty thousand yuan

一万元到五万元不等

Researcher

What is your understanding of custom furniture?

您对定制家具的理解是什么？

Informant 019

Customized furniture is unique, it is special and can be customized according to user preferences, a bit similar to the integration of designers and users, which can greatly improve user satisfaction and achieve a win-win situation.

定制家具首先是独一无二的，是特别的，可以根据用户喜好来定制，有点类似于设计者和用户融为一体的模式，这样可以极大提高用户满意度，达到双赢的局面。

Researcher

What do you know about custom furniture brand channels? (advertising or otherwise)

您了解定制家具品牌渠道是什么?（广告或其他）

Informant 019

Advertising, network channels, professional search platform

广告、电商和线下体验店

Researcher

How do you know about custom furniture?
您是怎么了解定制家具相关内容?

Informant 019
Mainly through the Internet to search to understand, but also from the mouth of friends around to understand, occasionally forced to understand through offline publicity.
主要通过网络去搜索了解，也会从身边朋友口中去了解，偶尔被迫通过线下宣传了解。

Researcher
What was your initial impression of the brand you chose? What was the initial understanding?
您对您选择的品牌最初印象是什么? 最初的理解是什么？

Informant 019
When choosing customized furniture for the first time, first of all, there is a very fresh experience, and the consumption experience has been improved. In addition to the consumption experience, I am also very satisfied with the design of the furniture. The fashionable and warm design style brings a warm feeling, which is very trustworthy. A good brand naturally has its place worth buying. When you initially choose the brand, it is inevitably affected by the brand effect. I try to customize the home for the first time with an free mentality, with a good experience.
第一次选择定制家具时，首先是非常新鲜的体验感，消费体验得到了前所未有的提高。除了消费体验外，对于家具的设计也非常满意，时尚温馨的设计风格带来温暖的感觉，非常值得信赖。好的品牌自然有其值得购买的地方，最初选择品牌时难免受到品牌效应的影响，抱着不出错的心态第一次尝试定制家居，体验感很好。

Researcher

Why do you choose this brand of custom furniture?
您选择该品牌的定制家具的原因是什么？

Informant 019
First of all, the appearance design is very fashionable and simple, in line with my aesthetic appreciation, and then the furniture material is safe, the color is also very good-looking.
首先外观设计非常时尚简约，符合本人的审美，其次家具的材质安全，色彩也很好看。

Researcher
What do you think are the advantages of custom furniture over finished furniture?
您认为相比成品家具，定制家具的优势是什么?

Informant 019
The style and collocation of choice are more free, more consistent with the decoration style, the utilization rate of space is high, and strong personalized and strong functionality. The most important thing is that it is fully in line with the user's psychological expectations, bringing consumers a better sense of experience.
选择的风格以及搭配比较自由，与装修风格更加契合，空间的利用率高，个性化强和功能性强，最重要的一点是它完全符合用户的心理预期，给消费者带来比较好的体验感。

Researcher
What do you think you should pay attention to when choosing custom furniture?
您觉得在选择定制家具时应该注意什么问题?

Informant 019
The appearance and color are defective, whether the size is reasonable, whether the structure is stable, whether the custom furniture accessories are complete, whether

the man-machine relationship is reasonable, and whether the other furniture in the home echoes.
外观和色彩有无瑕疵的地方，尺寸是否合理，构造是否稳定，定制家具配件是否齐全，人机关系是否合理，与家中其他家具是否相呼应。

Researcher
How often do you use cabinets, closets, and other custom furniture?
您使用橱柜、衣柜、和其他定制的家具的频率是如何的？

Informant 019
often use

经常使用
Researcher
Does the appearance of current custom furniture products meet your needs?
当前定制家具产品外观满足您的需求吗?

Informant 019
satisfied
满足

Researcher
Do current custom furniture products meet your needs with tactile details?
当前定制家具产品触觉细节满足您的需求吗?

Informant 019
satisfied
满足

Researcher

Does the current custom furniture fit your functional needs? Which need is not being met?

当前的定制家具是否符合您对产品功能的需求？哪一个需求没有得到满足？

Informant 019

Basically meet the needs, the function is more complete, some functions of the use of the frequency is not high. The design of the wardrobe can not be satisfied, the utilization rate is not high, the internal space planning needs to be further optimized and improved.

基本上符合需求，功能较为齐全，有些功能的使用频率不高。衣橱内部的设计利用空间不能够满足，利用率不高，内部空间规划有待进一步优化提高。

Researcher

Does the current custom furniture meet your need for product audibility or smell?

当前定制家具是否符合您对产品可听性或气味的需求？

Informant 019

satisfied

较为符合

Researcher

How do you open and close your custom furniture? How do you like to open and close the door?

您家定制家具开关门方式是什么样的？您喜欢哪种开关门方式？

Informant 019

Move between left and right, or push and pull

左右移动或是推拉式

Researcher

Will you share your successful decorating experience with others?
您会与别人分享您的装修成功经验吗?

Informant 019
Yes
会

Researcher
What do you think are the disadvantages of current custom furniture?
您觉得当前的定制家具的缺点是什么?

Informant 019
The price is high, the production cycle is long, there are after-sales problems, if the late encounter dissatisfied with the place is not easy to replace, the most fundamental disadvantage is the high price.
价格高，制作周期长，存在售后问题，若是后期遇到不满意的地方不太容易更换，最根本的缺点在于价格较高。

Researcher
What other features do you think can be added to custom furniture?
您觉得定制家具可以添加什么其他功能？

Informant 019
Hidden induction lamp and hidden storage space, furniture can be folded and telescopic to meet the use of large space.
隐藏感应灯和隐藏收纳空间，家具可以折叠伸缩，满足大空间使用功能。

Researcher
What aspects of custom furniture can provide more possibilities for users?

定制家具的哪些方面可以为用户提供更多的可能性?

Informant 019

In my opinion, it is mainly in terms of price. Secondly, unique functions and material selection are also a breakthrough point. Space utilization is what users always care about, because there are more possibilities in flexible space design.

我认为主要是价格方面，其次独特的功能和材料选择也是一大突破点，空间的利用是用户一直在意的，因为在空间灵活设计上也有更多可能。

Researcher

Okay, thank you for participating in this interview, thank you.

好的，感谢您参与本次访谈，谢谢。
