# Supplementary material for: Building a 4E interview-grounded theory model: A case study of demand factors for customized furniture
Source: PLoS One. 2023 Apr 27;18(4):e0282956. doi: 10.1371/journal.pone.0282956 (PMC10138260; doi:10.1371/journal.pone.0282956)
Supplement: S1 File — (ZIP) [file pone.0282956.s001.zip › transcript/transcript 020.pdf]

Informant : 020

Please note that the original transcript is in Simplified Chinese. The English translation is for internal communication among the author of this research, and it is not proofread. Potential linguistic errors may exist in the English translation.

Thank you for your willingness to participate and be interviewed here. My name is XXX, and I'm a PhD in the XXX University. Currently, I am working on a research project that focuses on collecting information about user demand when purchasing and using customized furniture. Throughout the interview, I will ask you a series of questions and you are encouraged to express your opinions and views freely. During the interview, I will ask you if I have questions about what you have said or if I need you to clarify a topic or concept.

感谢您愿意参加并在此接受采访。我叫 XXX，是 XXX 大学的博士。目前，我正在开展一个研究项目，主要收集在使用定制家具时的用户体验资料。在整个访谈中，我会问您一系列问题，我们鼓励您自由表达您的意见和观点。在访谈过程中，如果我对你所说的内容有疑问或需要您澄清一个主题或概念，我会向您询问。

Researcher

What is the square footage of your house?

你的房子的面积是多少?

Informant 020

110m2

Researcher

How big is your family? What's the family structure like?

您的家庭人数？家庭结构是什么样的？

Informant 020

Six people　　Parents, brother and me

家里面有 6 人 ，爷爷奶奶，父母，弟弟和我

Researcher

What is the style of furniture in the home?

家中家具是什么样式的？

Informant 0020

Home furniture is modern furniture, mainly solid wood and panel furniture.

家里的家具是现代化家具，主要是实木和板式家具。

Researcher

Where is the custom furniture placed? What are the main cabinets?

定制家具放置在哪里？主要是哪些柜体？

Informant 020

The custom furniture is an integral finishing style, placed in the bedroom, kitchen, and living room. The main cabinet includes wine cabinet, TV cabinet, cabinet, wardrobe and so on.

定制的家具是整体装修风格，放置在卧室，厨房，客厅。主要柜体有酒柜、电视柜、橱柜、衣柜等。

Researcher

What is your custom furniture style? Is it consistent with the home decor?

您家定制家具风格是什么样？和家中装修风格一致吗？

The style is modern and simple, and because the whole house is customized, the overall style is consistent.

风格是现代化的简约风格，因为是全屋定制，所以整体风格是一致的。

Researcher

How much do you spend on custom furniture?

你花多少钱在定制家具上?

Informant 020

It's probably tens of thousands yuan.

大概是几万块吧。

Researcher

What is your understanding of custom furniture?

您对定制家具的理解是什么？

Informant 020

Customized home can make personalized products according to our required styles and styles, in line with personalized consumer needs. In addition, customized homes can customize personalized furniture according to specific apartment types, effectively using every inch of space, increasing the practicality of furniture, which is also an important factor for many consumers to choose customized homes.

定制家居可以根据我们的需求做出个性化产品，符合个性化消费需求。另外，定制家居可以根据具体户型定制，有效利用每一寸空间，增加了家具的实用性，这也是众多消费者选择定制家居的重要因素。

Researcher

What do you know about custom furniture brand channels?

您了解定制家具品牌渠道是什么?

Informant 020

Ads or networks or referrals from friends

了解的渠道主要是通过亲戚朋友的介绍、参观家具城获得的。

Researcher

How do you know about custom furniture?

您是怎么了解定制家具相关内容?

Informant 020

Through the introduction of visiting furniture city shopping guides, as well as according to their preferences, in the public number and other search platforms to search for understanding.

通过参观家具城导购人员的介绍，还有根据自己的喜好，在公众号等搜索平台进行搜索了解。

Researcher

What was your initial impression of the brand you chose? What was the initial understanding?

您对您选择的品牌最初印象是什么？最初的理解是什么？

Informant 002

I choose Oriental Bang Tai, the initial impression is to see in a friend's home feel the overall style is more beautiful, the board is also an ecological environmental protection board, the overall is senior with texture.

我选择的是东方邦太，最初的印象是在朋友家里看到感觉整体风格比较好看，板材也是环保型的生态板，整体上显得高级有质感。

Researcher

Why do you choose this brand of custom furniture?

您选择该品牌的定制家具的原因是什么？

Informant020

Maximize home space and improve space utilization.

定制的家具能够最大限度地扩大家庭使用空间，提高空间利用率。

Researcher

What do you think are the advantages of custom furniture over finished furniture?

您认为相比成品家具，定制家具的优势是什么?

Informant 020

Customized home can make personalized products according to our required styles and styles, in line with personalized consumer needs.

定制家居可以根据我们的需求和喜好做出个性化产品，符合个性化消费需求。

Researcher

What do you think you should pay attention to when choosing custom furniture?

您觉得在选择定制家具时应该注意什么问题?

Informant020

Determine customized products according to the area of the room; Customized products and decoration styles should be unified; Resolutely eliminate supplies with low usage; Shop around and make a reasonable budget; Customized products and decoration styles should be unified.

首先在材质的选择上，选用对人体无害的环保型材料；其次，根据自己的预算，货比三家，选择适合自己的；对房屋的整体结构进行针对性设计，有效的利用空间。

Researcher

How often do you use cabinets, closets, and other custom furniture?

您使用橱柜、衣柜、和其他定制的家具的频率是如何的?

Informant 020

The cabinets and wardrobes used in the home are more frequent, while the wine cabinet is relatively used less often and mostly plays a decorative role.

家里使用的橱柜和衣柜的频率较高，酒柜相对来讲使用次数较少一些，大多只起到了装饰作用。

Researcher

Does the appearance of current custom furniture products meet your needs?

当前定制家具产品外观满足您的需求吗?

Informant 020

Now the custom furniture is mainly their own choice, but also just finished, so the appearance of the product is quite satisfied.

现在的定制家具主要是自己选择的，而且也是刚装完不就，所以对产品外观还是挺满足的。

Researcher

Do current custom furniture products meet your needs with tactile details?

当前定制家具产品触觉细节满足您的需求吗?

Informant 020

In terms of touch, it can meet my needs. The veneer of science and technology leather has the touch of wood texture.

在触觉上是可以满足我的需求的，科技皮的贴面有木头纹理的触感。

Researcher

Does the current custom furniture fit your functional needs? Which need is not being met?

当前的定制家具是否符合您对产品功能的需求？哪一个需求没有得到满足？

Informant 020

It basically meets my requirements for the use of the product, but sometimes the size of the items placed in the cabinet is different. It would be good if the space could be adjusted according to the size of the items.

基本符合我对产品的使用需求，但是有时候对柜子放置物品的大小不同，如果能根据物品大小调节空间就好了。

Researcher

Does the current custom furniture meet your need for product audibility or smell?

当前定制家具是否符合您对产品可听性或气味的需求?

Informant 020

No, the custom-made furniture in the house still smells like glue.

不符合，家里面刚装好的定制家具还有一些胶味。

Researcher

How do you open and close your custom furniture? How do you like to open and close the door?

您家定制家具开关门方式是什么样的？您喜欢哪种开关门方式?

Informant 020

The custom furniture in the home is basically open and close style, but there is a small room space, the choice of push-pull cabinet door.

家里的定制家具基本都是开关门样式的，但是有个房间空间较小，选用的推拉式的柜门。

I prefer to open and close the cabinet, open the door can be more intuitive to see what you want.

我更喜欢开关门的柜子，打开柜门能更直观的看到自己想要的东西。

Researcher

Will you share your successful decorating experience with others?

您会与别人分享您的装修成功经验吗?

Informant 020

Yes, because I think the overall style and use of the home is very good.

会的，因为我觉得家里面的整体风格和使用感受都挺好的。

Researcher

What do you think are the disadvantages of current custom furniture?

您觉得当前的定制家具的缺点是什么?

Informant 020

Until the finished product is actually made, it is difficult to grasp what it really looks like, because there is no finished product and no template, everything depends on the designer's drawings and experience. Custom furniture requires a certain production cycle.

真正做出来成品之前，很难把握它到底是什么样子，因为没有成品没有模版，一切全凭设计师的图纸和经验。定制家具需要一定的制作周期。

Researcher

What other features do you think can be added to custom furniture?

您觉得定制家具可以添加什么其他功能？

Informant 020

I think custom furniture can be combined with modern technology to add more intelligent functions and better facilitate our lives

我觉得定制家具可以结合现代科技增加更多的智能化功能，更好的方便我们的生活。

Researcher

What aspects of custom furniture can provide more possibilities for users?

定制家具的哪些方面可以为用户提供更多的可能性?

Informant 020

In terms of personalization, it can be designed according to our needs

In terms of space utilization, maximize the use and reduce the waste of space

In terms of quality, you can choose your own materials to ensure quality

个性化方面，可以根据我们的需求来设计。空间利用方面，最大程度的利用，减少空间的浪费。质量方面，可以自己选择材料保证质量。

Researcher

Okay, thank you for participating in this interview and have a great life.

好的，感谢您对本次访谈的参与，祝您生活愉快。
