# Supplementary material for: Building a 4E interview-grounded theory model: A case study of demand factors for customized furniture
Source: PLoS One. 2023 Apr 27;18(4):e0282956. doi: 10.1371/journal.pone.0282956 (PMC10138260; doi:10.1371/journal.pone.0282956)
Supplement: S1 File — (ZIP) [file pone.0282956.s001.zip › transcript/transcript 021.pdf]

Informant : 021

Please note that the original transcript is in Simplified Chinese. The English translation is for internal communication among the author of this research, and it is not proofread. Potential linguistic errors may exist in the English translation.

Thank you for your willingness to participate and be interviewed here. My name is XXX, and I'm a PhD in the XXX University. Currently, I am working on a research project that focuses on collecting information about user demand when purchasing and using customized furniture. Throughout the interview, I will ask you a series of questions and you are encouraged to express your opinions and views freely. During the interview, I will ask you if I have questions about what you have said or if I need you to clarify a topic or concept.

感谢您愿意参加并在此接受采访。我叫 XXX，是 XXX 大学的博士。目前，我正在开展一个研究项目，主要收集在使用定制家具时的用户体验资料。在整个访谈中，我会问您一系列问题，我们鼓励您自由表达您的意见和观点。在访谈过程中，如果我对你所说的内容有疑问或需要您澄清一个主题或概念，我会向您询问。

Researcher

Are you ready?

您准备好了吗？

Informant 021

Yes.

准备好了。

Researcher

First,some questions about yourself.How old are you now?

首先是关于您个人的一些问题。请问您现在的年龄是多少？

Informant 002

I am 26 years old.

我今年 26 岁。

Researcher

What kind of work are you doing now?

请问您现在从事什么工作呢？

Informant 002

I run my own restaurant.

我是自己开饭店的。

Researcher

What is the square footage of your house?

您的房子的面积是多少?

Informant 021

126 square meters.

126 平方米。

Researcher

How big is your family? What's the family structure like?

您的家庭人数？家庭结构是什么样的？

Informant 021

There are four of us. I live with my parents and my brother.

有 4 个人，我和爸妈还有哥哥住在一起。

Researcher

What is the style of furniture in the home?

家中家具是什么样式的？

Informant 021

The furniture style is mainly Chinese style, but there are also some Western-style furniture. Among them, the most common furniture includes sofa, tea table, dining table and chair, TV cabinet, bookcase and so on.

家具的款式以中式为主，但也有一些西式家具。其中，最常见的家具包括沙发、茶几、餐桌椅、电视柜、书柜等。

Researcher

Where is the custom furniture placed? What are the main cabinets?

定制家具放置在哪里?主要是哪些柜体？

Informant 021

My custom furniture is mainly placed in the bedroom. Mainly inlaid log cabinet, perfect for bedroom.

我家的定制家具主要被放置在卧室。主要是内镶式原木柜体，非常适合放置在卧室中。

Researcher

What is your custom furniture style? Is it consistent with the home decor?

您家定制家具风格是什么样？和家中装修风格一致吗？

Informant 021

In the family decoration style, Chinese style is also the main. Because like this traditional cultural atmosphere, so in the furniture configuration also made the corresponding adjustment.

在家庭的装修风格上，也是以中式风为主。因为喜欢这种传统的文化氛围，所以在家具的配置上也做了相应的调整。

Researcher

How much do you spend on custom furniture?

您花了多少钱在定制家具上?

Informant 021

It cost about 200,000 yuan. Even if there is a certain cost, but also believe that these collocation of custom furniture can meet their needs.

大约花费了 20 万元。即使是有一定的费用，但也相信这些搭配好的定制家具能够满足自己的需要。

Researcher

What is your understanding of custom furniture?

您对定制家具的理解是什么？

Informant 021

Custom furniture is furniture that can be completely customized according to the needs of their own family. For example, like what style of furniture, need what kind of cabinet size, can be customized according to their own preferences.

定制家具就是可以完全根据自己的家庭需要进行定制的家具。比如喜欢什么款式的家具，需要什么样的柜体大小等，都可以根据自己的喜好来定制。

Researcher

What do you know about custom furniture brand channels? (advertising or otherwise)

您了解定制家具品牌渠道是什么?（广告或其他）

Informant 021

They are mainly recommended by advertisements and word of mouth from friends, which enables me to find the most suitable custom furniture brands.

主要都是通过广告和朋友的口碑来推荐的，能够让我寻找最适合的定制家具品牌。

Researcher

How do you know about custom furniture?

您是怎么了解定制家具相关内容?

Informant 021

Consult articles and consult relevant people is the main way to understand the content of customized furniture. Through various inquiries and learning, the understanding of custom furniture will become more in-depth.

查阅文章和向相关人士咨询是了解定制家具相关内容的主要途径。通过多方面的询问和学习，对定制家具的理解会变得更加深入。

Researcher

What was your initial impression of the brand you chose? What was the initial understanding?

您对您选择的品牌最初印象是什么?最初的理解是什么？

Informant 021

The packaging and advertising of this brand are very atmospheric, well-known and professional, which can meet most needs and make people have the desire to buy.

这个品牌的包装和广告都很大气，知名度高，专业性强，能满足大部分需求，让人有购买的欲望。

Researcher

Why do you choose this brand of custom furniture?

您选择该品牌的定制家具的原因是什么？

Informant 021

The final choice of brand is because other aesthetic suits their needs. This brand is well-known and professional. It can provide many furniture requirements and customized furniture solutions to meet their needs.

最终选择的品牌是因为其它审美符合自己的需要。这个品牌的知名度很高，专业性也很强，可以提供很多家具方面的需求， 能够满足自己需要的定制家具方案。

Researcher

What do you think are the advantages of custom furniture over finished furniture?

您认为相比成品家具，定制家具的优势是什么?

Informant 021

Custom furniture has high space utilization, good environmental performance, and increasingly cheaper prices.

定制家具的空间利用率高，环保性能好，价格也越来越便宜。

Researcher

What do you think you should pay attention to when choosing custom furniture?

您觉得在选择定制家具时应该注意什么问题?

Informant 021

Check that the size is appropriate and that the build is stable and reasonable.

要检查尺寸是否合适，构建是否稳定合理。

Researcher

How often do you use cabinets, closets, and other custom furniture?

您使用橱柜、衣柜、和其他定制的家具的频率是如何的?

Informant 021

Cabinets and wardrobes are used more frequently, while others are used in normal frequency in daily life.

橱柜和衣柜的使用频率较高，其他的在日常生活中用的就是正常频率。

Researcher

Does the appearance of current custom furniture products meet your needs?

当前定制家具产品外观满足您的需求吗?

Informant 021

The look of current custom furniture products largely meets my needs. I like the personalized features of customized furniture. They can be customized according to my needs to meet my preferences in appearance and style, so that my home is more comfortable and warm.

当前定制家具产品的外观很大程度上能够满足我的需求。我喜欢定制家具的个性化特点，它们能够根据我的需求定制出符合我喜好的外观和风格，让我的家更加舒适、温馨。

Researcher

Do current custom furniture products meet your needs with tactile details?

当前定制家具产品触觉细节满足您的需求吗?

Informant 021

The tactile detail of the custom furniture is also excellent. I love the feel and texture of custom-made furniture, which is not only comfortable but also load-bearing enough for everyday use.

定制家具的触觉细节也十分出色。我喜欢定制家具的手感和质感，它们不仅舒适，而且承重能力强，可以胜任日常的使用。

Researcher

Does the current custom furniture fit your functional needs? Which need is not being met?

当前的定制家具是否符合您对产品功能的需求？哪一个需求没有得到满足？

Informant 021

Most of the custom-made furniture meets my needs, but some cabinets are a bit cumbersome and take a while to find, which is probably one of the drawbacks.

大部分定制家具能够满足我的需求，但是有一些橱柜设计有点繁琐，需要一些时

间才能找到所需物品，这可能是它的缺点之一。

Researcher

Does the current custom furniture meet your need for product audibility or smell?

当前定制家具是否符合您对产品可听性或气味的需求？

Informant 021

Current custom furniture not only excels in noise reduction, but also smells great. They are mostly made of environmentally friendly materials and do not have unpleasant smells.

当前的定制家具不仅在减噪方面表现出色，而且气味也非常不错。它们大多由环保材料制成，没有令人不舒服的味道。

Researcher

How do you open and close your custom furniture? How do you like to open and close the door?

您家定制家具开关门方式是什么样的？您喜欢哪种开关门方式？

Informant 021

There are two ways to open and close the custom furniture in my home. One is the pull type and the other is the folding type. I personally prefer the pull-out ones because they are more convenient and less labor-intensive.

我家定制家具的开关门方式有两种，一种是抽拉式，另一种是折叠式。我个人更喜欢抽拉式，因为它们更方便，省力。

Researcher

Will you share your successful decorating experience with others?

您会与别人分享您的装修成功经验吗?

Informant 021

I will share my decorating success with others. I think it is a good way to communicate and share. While sharing, I can also learn a lot of useful information from other people's experience.
我会和别人分享我的装修成功经验。我认为这是一种很好的交流和分享方法，我在分享的同时也可以从别人的经验中学到很多有用的信息。

Researcher
What do you think are the disadvantages of current custom furniture?
您觉得当前的定制家具的缺点是什么?

Informant 021
In my opinion, the main disadvantages of current customized furniture are inconvenient to move, long customization cycle and high price. These factors may deter some people.
我认为当前定制家具的缺点主要是不方便移动、定制周期长、价格高。这些因素可能会让一些人望而却步。

Researcher
What other features do you think can be added to custom furniture?
您觉得定制家具可以添加什么其他功能？

Informant 021
I think custom furniture can add more malleable space. For example, by installing drawers under my bed, I can add some storage space and make my home more tidy and organized.
我认为定制家具可以添加更多的延展性空间。比如，在床下安装抽屉，可以增加一些收纳空间，使我的家可以更加整洁有序。

Researcher
What aspects of custom furniture can provide more possibilities for users?

定制家具的哪些方面可以为用户提供更多的可能性?

Informant 021

The storage, material selection, design and process of custom furniture can provide users with more possibilities. For example, adding storage functions, providing more material options, and better design and craftsmanship can make the experience of our consumers even better.

定制家具的收纳、材料选择、设计和工艺等方面可以为用户提供更多的可能性。比如，增加收纳功能，提供更多的材料选择方案，以及更好的设计和工艺，可以使我们消费者的体验更加出色。

Researcher

Okay, thank you for participating in this interview and have a great life.

好的，感谢您对本次访谈的参与，祝您生活愉快。
