# Supplementary material for: Building a 4E interview-grounded theory model: A case study of demand factors for customized furniture
Source: PLoS One. 2023 Apr 27;18(4):e0282956. doi: 10.1371/journal.pone.0282956 (PMC10138260; doi:10.1371/journal.pone.0282956)
Supplement: S1 File — (ZIP) [file pone.0282956.s001.zip › transcript/transcript 022.pdf]

Informant : 022

Please note that the original transcript is in Simplified Chinese. The English translation is for internal communication among the author of this research, and it is not proofread. Potential linguistic errors may exist in the English translation.

Thank you for your willingness to participate and be interviewed here. My name is XXX, and I'm a PhD in the XXX University. Currently, I am working on a research project that focuses on collecting information about user demand when purchasing and using customized furniture. Throughout the interview, I will ask you a series of questions and you are encouraged to express your opinions and views freely. During the interview, I will ask you if I have questions about what you have said or if I need you to clarify a topic or concept.

感谢您愿意参加并在此接受采访。我叫 XXX，是 XXX 大学的博士。目前，我正在开展一个研究项目，主要收集在使用定制家具时的用户体验资料。在整个访谈中，我会问您一系列问题，我们鼓励您自由表达您的意见和观点。在访谈过程中，如果我对你所说的内容有疑问或需要您澄清一个主题或概念，我会向您询问。

Researcher

Are you ready?

您准备好了吗？

Informant 022

Yes.

准备好了。

Researcher

First, some questions about yourself. How old are you now?

首先是关于您个人的一些问题。请问您现在的年龄是多少？

Informant 022

I am 32 years old.

我今年 32 岁。

Researcher

What kind of work are you doing now?

请问您现在从事什么工作呢？

Informant 022

I'm a driver.

我是一名司机。

Researcher

What is the square footage of your house?

你的房子的面积是多少?

Informant 022

50 square meters.

50 平方米。

Researcher

How many people live in your house now?

您的房子现在几个人居住?

Informant 022

I live alone.

我一个人住。

Researcher

What is the style of furniture in the home?

家中家具是什么样式的？

Informant 022

Simple atmosphere, more warm.

简约大气的，比较温馨的。

Researcher

Where is the custom furniture placed? What are the main cabinets?

定制家具放置在哪里？主要是哪些柜体？

Informant 022

Mainly in the bedroom, kitchen and cloakroom. Mainly wardrobes, cupboards, etc.

主要在卧室、厨房以及衣帽间。主要是衣柜、橱柜等。

Researcher

What is your custom furniture style? Is it consistent with the home decor?

您家定制家具风格是什么样？和家中装修风格一致吗？

Informant 022

Custom furniture is also simple and generous style. Consistent with the decor of the home.

定制的家具也是以简约大方的风格为主。和家中的装修风格一致。

Researcher

How much do you spend on custom furniture?

你花多少钱在定制家具上?

Informant 022

It's about 3-5w.

大约在 3-5w。

Researcher

What is your understanding of custom furniture?

您对定制家具的理解是什么？

Informant 022

I think customized furniture is to create the most suitable for their own family decoration style and function of furniture according to their own needs.

我认为的定制家具是根据自己的需要打造出最适合自己的家庭的装修风格以及使用功能的家具。

Researcher

What do you know about custom furniture brand channels? (advertising or otherwise)

您了解定制家具品牌渠道是什么?（广告或其他）

Informant 022

It's usually a recommendation from someone close to you.

一般是来自于身边的朋友的推荐。

Researcher

How do you know about custom furniture?

您是怎么了解定制家具相关内容?

Informant 022

I learned about customized furniture through the recommendation of friends around me. Friends told me that customized furniture can be customized according to personal needs and space size of the perfect furniture, and the quality and materials are better, the price is more affordable. I was very interested in it, so I started learning

about it and actually checking out some brands.
我是通过身边的朋友的推荐了解到定制家具相关内容的。朋友告诉我，定制家具可以根据个人需求和空间大小定制出完美的家具，而且质量和用料都比较好，价格也比较实惠。我对此非常感兴趣，就开始了解相关知识，并实际考察了一些品牌。

Researcher
What was your initial impression of the brand you chose? What was the initial understanding?
您对您选择的品牌最初印象是什么？最初的理解是什么？

Informant 022
When I first learned about the custom furniture brand I chose, my impression was that the price was relatively affordable and the reputation was very good. However, I have some misgivings because I have heard that custom furniture is expensive and the workmanship is sometimes good and sometimes bad.
当我初次了解到选择的定制家具品牌时，我对它的印象是价格比较实惠，而且口碑非常好。但是，我也有一些顾虑，因为我听说定制家具的价格比较昂贵，而且做工有时好有时坏。

Researcher
Why do you choose this brand of custom furniture?
您选择该品牌的定制家具的原因是什么？

Informant 022
The reason why I choose this custom furniture brand is that its appearance and function meet my needs. It can be customized to my actual situation, and the price is relatively affordable, so I chose it.
选择这个定制家具品牌的原因是因为它的外观和功能都很满足我的需求。它可以

根据我的实际情况进行定制，而且价格也比较实惠，所以我选择了它。

Researcher

What do you think are the advantages of custom furniture over finished furniture?

您认为相比成品家具，定制家具的优势是什么？

Informant 022

What do you think are the advantages of custom furniture over finished furniture?

定制家具可以根据家庭的装修风格以及户型定制出最适合的风格以及类型的家具。

Researcher

What do you think you should pay attention to when choosing custom furniture?

您觉得在选择定制家具时应该注意什么问题？

Informant 022

Attention should be paid to the materials used, the manufacturing process and whether the price is affordable, and whether the designer understands the needs of the customer.

应该注意使用的材料、制作工艺以及价格是否实惠，设计师是否能理解客户的需求。

Researcher

How often do you use cabinets, closets, and other custom furniture?

您使用橱柜、衣柜、和其他定制的家具的频率是如何的？

Informant 022

Compared with other furniture, cabinets and wardrobes are customized more frequently, because the most can reflect the design style of a home is in the design of cabinets.

相比较其他家具，橱柜和衣柜的定制频率更加高，因为最能体现一个家的设计风格就在橱柜等的设计。

Researcher

Does the appearance of current custom furniture products meet your needs?

当前定制家具产品外观满足您的需求吗?

Informant 022

I think it is very satisfying for my needs of furniture, practical and simple.

我认为是十分满足我对于家具的需求的，实用而简洁。

Researcher

Do current custom furniture products meet your needs with tactile details?

当前定制家具产品触觉细节满足您的需求吗?

Informant 022

When I customize furniture, my biggest requirement is that the materials selected are good, so the tactile details of the customized cabinets can meet my needs.

我在定制家具时对其最大的要求就是选用的材料是良好的，所以定制的柜子的产品触觉细节可以满足我的需求。

Researcher

Does the current custom furniture fit your functional needs? Which need is not being met?

当前的定制家具是否符合您对产品功能的需求？哪一个需求没有得到满足？

Informant 022

My customized furniture meets my functional requirements for the product and has a good storage effect while not occupying too much spare space. However, the only drawback is that there are no small storage compartments for me to place some small

items.

我定制的家具符合了我对于产品的功能需求，在并不过多占据空余空间的同时拥有很好的收纳效果，但是相比之下，美中不足的是没有设置一些小的收纳隔间给我放置一些小的物品。

Researcher

Does the current custom furniture meet your need for product audibility or smell?

当前定制家具是否符合您对产品可听性或气味的需求？

Informant 022

Satisfied. In my opinion, the smell of wood is a very reassuring aroma, and the faint wood scent can calm me down.

满足。在我看来，木头的香气是一种很让人安心的香气，淡淡的木质香可以让我平静下来。

Researcher

How do you open and close your custom furniture? How do you like to open and close the door?

您家定制家具开关门方式是什么样的？您喜欢哪种开关门方式？

Informant 022

How do you open and close your custom furniture? How do you like to open and close the door?

我的定制家具开关门方式是推拉式柜门。相比较平移式的柜门，推拉式的柜门更加方便整理，而且更加美观。在我家的空间大小和风格选择方面，这种开门方式非常适合。

Researcher

Will you share your successful decorating experience with others?

您会与别人分享您的装修成功经验吗?

Informant 022

It will. In my opinion, the spread of a brand needs word of mouth from customers. If you can save your friends a lot of time to make choices and make mistakes, why not?

会的。我认为一个品牌的传播需要客户的口口相传，如果可以给身边的朋友省去很多选择和犯错的时间，何乐而不为呢？

Researcher

What do you think are the disadvantages of current custom furniture?

您觉得当前的定制家具的缺点是什么?

Informant 022

The disadvantage of the current custom furniture is that its cost is high, and the weight is also relatively large, so the relocation will be more troublesome. In addition, the construction and installation time is long, you have to wait a while to enjoy the custom-made furniture.

当前定制家具的缺点是它的造价较高，而且重量也比较大，所以搬迁会比较麻烦。此外，制作和安装的工期也比较长，需要等待一段时间才能享受到定制家具带来的乐趣。

Researcher

What other features do you think can be added to custom furniture?

您觉得定制家具可以添加什么其他功能？

Informant 022

I think custom furniture can be combined with many existing smart homes, such as the popular Xiao Ai smart home, to add vitality to the originally dull custom furniture industry.

我认为定制家具可以和许多现有的智能家居的结合，比如现下流行的小爱智能家居等，给原本沉闷的定制家具业增添一份活力。

Researcher

What aspects of custom furniture can provide more possibilities for users?

定制家具的哪些方面可以为用户提供更多的可能性?

Informant 022

Custom furniture can provide users with more possibilities in the selection of shape, style, function and materials. Users can choose the perfect custom furniture according to their own needs, making home life more convenient and beautiful. For example, in terms of function selection, users can choose furniture with drawers or more storage space according to their needs to meet their needs. In terms of material selection, users can choose their favorite colors and materials, as well as choose more environmentally friendly and healthy materials according to the family environmental awareness.

定制家具可以在造型选择、风格选择、功能选择、用材选择等方面为用户提供更多的可能性。用户可以根据自己的需求选择完美的定制家具，使家居生活更加便利和美好。例如，在功能选择方面，用户可以根据自己的需要选择带有抽屉或者更多储物空间的家具，以满足自己的需求。在用材选择方面，用户可以选择自己喜欢的颜色和材料，以及根据家庭环保意识选择更加环保健康的材料。

Researcher

Okay, thank you for participating in this interview and have a great life.

好的，感谢您对本次访谈的参与，祝您生活愉快。
