# Supplementary material for: Building a 4E interview-grounded theory model: A case study of demand factors for customized furniture
Source: PLoS One. 2023 Apr 27;18(4):e0282956. doi: 10.1371/journal.pone.0282956 (PMC10138260; doi:10.1371/journal.pone.0282956)
Supplement: S1 File — (ZIP) [file pone.0282956.s001.zip › transcript/transcript 023.pdf]

Informant : 023

Please note that the original transcript is in Simplified Chinese. The English translation is for internal communication among the author of this research, and it is not proofread. Potential linguistic errors may exist in the English translation.

Thank you for your willingness to participate and be interviewed here. My name is XXX, and I'm a PhD in the XXX University. Currently, I am working on a research project that focuses on collecting information about user demand when purchasing and using customized furniture. Throughout the interview, I will ask you a series of questions and you are encouraged to express your opinions and views freely. During the interview, I will ask you if I have questions about what you have said or if I need you to clarify a topic or concept.

感谢您愿意参加并在此接受采访。我叫 XXX，是 XXX 大学的博士。目前，我正在开展一个研究项目，主要收集在使用定制家具时的用户体验资料。在整个访谈中，我会问您一系列问题，我们鼓励您自由表达您的意见和观点。在访谈过程中，如果我对你所说的内容有疑问或需要您澄清一个主题或概念，我会向您询问。

Researcher

Are you ready?

您准备好了吗？

Informant 023

Yes.

准备好了。

Researcher

First, some questions about yourself. How old are you now?

首先是关于您个人的一些问题。请问您现在的年龄是多少？

Informant 023

I am 34 years old.

我今年 34 岁。

Researcher

What kind of work are you doing now?

请问您现在从事什么工作呢？

Informant 023

I'm a teacher.

我是一名老师。

Researcher

What is the square footage of your house?

你的房子的面积是多少?

Informant 023

220 square meters.

220 平方米。

Researcher

How big is your family? What's the family structure like?

您的家庭人数？家庭结构是什么样的？

Informant 023

Three people. My parents and me.

3 人。父母亲以及我。

Researcher

What is the style of furniture in the home?

家中家具是什么样式的？

Informant 023

The furniture in the house is all in Chinese and European style. Chinese style furniture color is mostly dark, simple lines, focus on practicality, European style furniture is focused on gorgeous, exquisite appearance, usually using bright colors and delicate carving.

家里的家具都是中式和欧式风格的。中式风格的家具色调大多是深色，线条简约，注重实用性，欧式风格的家具则注重华丽、精美的外观，通常采用明亮的色调和细腻的雕刻。

Researcher

Where is the custom furniture placed? What are the main cabinets?

定制家具放置在哪里? 主要是哪些柜体？

Informant 023

Mainly in the bedroom, living room and cloakroom. Mainly storage cabinets, wardrobes, tables and so on.

主要在卧室、客厅以及衣帽间。主要是收纳柜、衣柜、桌子等。

Researcher

What is your custom furniture style? Is it consistent with the home decor?

您家定制家具风格是什么样？和家中装修风格一致吗？

Informant 023

Our custom furniture style and home decoration style basically maintain the same, to Chinese simple and generous style. The feature of custom furniture is that it can be made according to our needs and space size, more intimate and personalized.

我们家定制的家具风格与家中装修风格基本保持一致，以中式简约大方的风格为

主。定制家具的特点是可以根据我们的需求和空间尺寸来制作，更加贴心和个性化。

Researcher

What is your understanding of custom furniture?

您对定制家具的理解是什么？

Informant 023

I think custom furniture can make their own more personalized, can effectively use the space everywhere, make home more beautiful, practical.

我认为定制家具能让自家更具个性化，能有效利用好各处空间，让家居更美观，实用。

Researcher

What do you know about custom furniture brand channels?

您了解定制家具品牌渠道是什么?

Informant 023

Understand and choose through a variety of ways such as the introduction of family and friends, as well as customized furniture publicity advertisements.

通过家人朋友的介绍以及定制家具宣传广告等多种途径了解和选择。

Researcher

How do you know about custom furniture?

您是怎么了解定制家具相关内容?

Informant 023

Go to the furniture city to investigate, Xiaohongshu and other software advertising introduction.

去家具城调研，小红书等软件的广告介绍。

Researcher

What was your initial impression of the brand you chose? What was the initial understanding?

您对您选择的品牌最初印象是什么？最初的理解是什么？

Informant 023

My initial impression of choosing a brand is that it fits in with my own style and is cost-effective. The initial understanding is to reflect the style of the whole house through furniture details.

我对选择品牌的最初印象是与自家风格适配，性价比高。最初的理解是通过家具细节体现全屋格调。

Researcher

Why do you choose this brand of custom furniture?

您选择该品牌的定制家具的原因是什么？

Informant 023

Because it is cost-effective, in line with their own decoration style and daily life needs.

因为它的性价比高，符合自家装修风格和日常生活需求。

Researcher

What do you think are the advantages of custom furniture over finished furniture?

您认为相比成品家具，定制家具的优势是什么？

Informant 023

Compared with finished furniture, customized furniture has the advantage that it can meet the different needs of different people, be more elaborate and last longer.

相比成品家具，定制家具的优势在于可以满足不同人的不同需求，更精细制作，使用寿命更长久。

Researcher

What do you think you should pay attention to when choosing custom furniture?

您觉得在选择定制家具时应该注意什么问题？

Informant 023

Pay attention to the materials and craftsmanship used in custom furniture, consider your own style and lifestyle, and shop around.

要注意定制家具使用的材料和工艺，考虑自家风格和生活习惯，货比三家。

Researcher

How often do you use cabinets, closets, and other custom furniture?

您使用橱柜、衣柜、和其他定制的家具的频率是如何的？

Informant 023

It is used frequently every day, because the storage and sorting of clothes and daily necessities cannot be separated from the cabinet.

每天都使用的很频繁，因为衣服和生活用品的收纳整理离不开橱柜。

Researcher

Does the appearance of current custom furniture products meet your needs?

当前定制家具产品外观满足您的需求吗?

Informant 023

The look of my current custom-made furniture mostly meets my needs, but some of it has passed the age and needs to be updated.

我目前定制的家具外观大致上满足我的需求，但有一些已经经过岁月洗礼需要更

新迭代。

Researcher

Do current custom furniture products meet your needs with tactile details?

当前定制家具产品触觉细节满足您的需求吗?

Informant 023

Roughly, because sometimes carelessness can cause pain by touching the edges and corners.

大致符合，因为有时粗心会碰到边边角角引起疼痛。

Researcher

Does the current custom furniture fit your functional needs? Which need is not being met?

当前的定制家具是否符合您对产品功能的需求？哪一个需求没有得到满足？

Informant 023

Roughly, but I wish the details were more cleverly divided and had a more interesting look.

大致符合，但我希望收纳细节可以得到更巧妙的划分，外观更具趣味性。

Researcher

Does the current custom furniture meet your need for product audibility or smell?

当前定制家具是否符合您对产品可听性或气味的需求？

Informant 023

In line with my needs, although customized furniture is not much different from solid wood furniture, the sound and smell of wood products make me feel more comfortable and secure.

符合我的需求，虽然是定制家具也和实木家具带给人的感觉没有太大差距，木制品的声音和香味让我更惬意安心。

Researcher

How do you open and close your custom furniture? How do you like to open and close the door?

您家定制家具开关门方式是什么样的？您喜欢哪种开关门方式？

Informant 023

Flat open and push-pull. I prefer push-pull. It's more convenient.

平开和推拉式。我更喜欢推拉式的，更方便。

Researcher

Will you share your successful decorating experience with others?

您会与别人分享您的装修成功经验吗?

Informant 023

It will. Good brands deserve to be known by more people.

会的。好的品牌值得被更多人知晓。

Researcher

What do you think are the disadvantages of current custom furniture?

您觉得当前的定制家具的缺点是什么?

Informant 023

In my opinion, the disadvantages of customized furniture are that the final effect cannot be seen directly, the waiting period is long, and it will be troublesome to replace the furniture.

我认为定制家具的缺点是不能直观看到最终效果，等待周期长，想换位置放会很

麻烦。

Researcher

What other features do you think can be added to custom furniture?

您觉得定制家具可以添加什么其他功能？

Informant 023

I think it can be linked with other smart homes, or through clever splitting or folding transform shape, to adapt to other Spaces.

我认为可以和其他智能家居联动，或者通过巧妙的拆分或折叠变换造型，适应其他空间。

Researcher

What aspects of custom furniture can provide more possibilities for users?

定制家具的哪些方面可以为用户提供更多的可能性?

Informant 023

At present, customized furniture has provided more possibilities, such as personalized design in modeling style, diversified choice in function and innovation in home intelligence.

目前定制家具已经提供了更多的可能性，例如在造型风格上的个性化设计、在功能上的多样化选择以及在家居智能化方面的创新。

Researcher

Okay, thank you for participating in this interview and have a great life.

好的，感谢您对本次访谈的参与，祝您生活愉快。
