# Supplementary material for: Building a 4E interview-grounded theory model: A case study of demand factors for customized furniture
Source: PLoS One. 2023 Apr 27;18(4):e0282956. doi: 10.1371/journal.pone.0282956 (PMC10138260; doi:10.1371/journal.pone.0282956)
Supplement: S1 File — (ZIP) [file pone.0282956.s001.zip › transcript/transcript 024.pdf]

Informant : 024

Please note that the original transcript is in Simplified Chinese. The English translation is for internal communication among the author of this research, and it is not proofread. Potential linguistic errors may exist in the English translation.

Thank you for your willingness to participate and be interviewed here. My name is XXX, and I'm a PhD in the XXX University. Currently, I am working on a research project that focuses on collecting information about user demand when purchasing and using customized furniture. Throughout the interview, I will ask you a series of questions and you are encouraged to express your opinions and views freely. During the interview, I will ask you if I have questions about what you have said or if I need you to clarify a topic or concept.

感谢您愿意参加并在此接受采访。我叫 XXX，是 XXX 大学的博士。目前，我正在开展一个研究项目，主要收集在使用定制家具时的用户体验资料。在整个访谈中，我会问您一系列问题，我们鼓励您自由表达您的意见和观点。在访谈过程中，如果我对你所说的内容有疑问或需要您澄清一个主题或概念，我会向您询问。

Researcher

Are you ready?

您准备好了吗？

Informant 024

Yes.

准备好了。

Researcher

First, some questions about yourself. How old are you now?

首先是关于您个人的一些问题。请问您现在的年龄是多少？

Informant 024

I am 34 years old.

我今年 34 岁。

Researcher

What kind of work are you doing now?

请问您现在从事什么工作呢？

Informant 024

I'm a kindergarten teacher.

我是一名幼儿园老师。

Researcher

What is the square footage of your house?

你的房子的面积是多少?

Informant 024

160 square meters.

160 平米。

Researcher

How big is your family? What's the family structure like?

您的家庭人数？家庭结构是什么样的？

Informant 024

Three people, respectively, my husband and children.

3 人，分别是我和老公孩子。

Researcher

What is the style of furniture in the home?

家中家具是什么样式的？

Informant 024

Mainly in the house to customize solid wood furniture, partial Chinese style. A small amount of smart iot furniture is complemented.

主要是以全屋定制实木家具为主，偏中式风格。少量智能物联网家具为辅。

Researcher

Where is the custom furniture placed? What are the main cabinets?

定制家具放置在哪里？主要是哪些柜体？

Informant 024

Custom furniture is mainly distributed in the living room, dining room, study, cloakroom, tea room, master bedroom, second bedroom. Mainly for the living room in the TV cabinet, tea table, solid wood sofa; Dining table, matching chairs; Bookcase, desk in the study; A closet in a cloakroom; A tea table in the tea room; Wardrobes, beds, bedside tables in bedrooms.

定制家具主要分布在客厅、餐厅、书房、衣帽间、茶室、主卧、次卧。主要为客厅中的电视机柜、茶几、实木沙发；餐厅中的餐桌、配套椅子；书房中的书柜、书桌；衣帽间内的衣橱；茶室内的茶桌；卧房内的衣柜、床、床头柜。

Researcher

What is your custom furniture style? Is it consistent with the home decor?

您家定制家具风格是什么样？和家中装修风格一致吗？

Informant 024

My custom furniture style partial Chinese style, color choice simple elegant clean, soft and quiet, and home decoration style.

我家的定制家具风格偏中式风格，颜色选择方面素雅干净，柔和恬淡，和家中装修风格一致。

Researcher
How much do you spend on custom furniture?
你花多少钱在定制家具上?

Informant 024
About 30w.
30w 左右。

Researcher
What is your understanding of custom furniture?
您对定制家具的理解是什么？

Informant 024
Compared with traditional furniture, customized furniture can be customized according to the special needs of users, for the user's house, for the user's ideas and needs, combined with the experience and craft of designers, a set of furniture suitable for users. From these custom furniture, can reflect the style of the user.
与传统家具相比较，定制家具可以根据用户特殊需求，为用户的房子，为用户的想法和需求，结合设计师的经验和工艺，量身定做一套合适用户的家具。从这些定制家具上，可以反映出用户的风格。

Researcher
What do you know about custom furniture brand channels?
您了解定制家具品牌渠道是什么?

Informant 024
Various furniture fairs, official website interpretation, offline store visits.

企业各种家具展销会、官网解说、线下门店参观。

Researcher

How do you know about custom furniture?

您是怎么了解定制家具相关内容?

Informant 024

hrough various furniture fairs, offline store visits and surveys. There will be a variety of customized home furnishing enterprises on the season of new furniture display, you can close touch experience. Log in the official website of famous customized furniture, you can view the design concept of customized furniture, watch furniture video commentary, and even 3D simulation images to simulate the customized furniture and home placement environment.

通过企业各种家具展销会，线下门店参观调研。展销会上会有各种定制家居企业对当季新品家具进行展示，可以近距离体验触摸。登录知名定制家具官网，可以查看定制家具的设计理念，观看家具视频解说，甚至还有 3D 模拟影像，对定制家具与居家摆放环境进行模拟。

Researcher

What was your initial impression of the brand you chose? What was the initial understanding?

您对您选择的品牌最初印象是什么?最初的理解是什么？

Informant 024

The brand I chose for whole-house customized furniture is "Sophia". My initial impression of "Sophia" is that when I visited the official website and offline stores, the style of "Sophia" whole-house customized furniture is similar to my preference, with a wide range of options, which can provide users with more quality experience and more personalized home furnishing choices.

我选择的全屋定制家具的品牌是“索菲亚”，“索菲亚”给我的最初印象是，在官网和线下门店参观时，“索菲亚”全屋定制家具风格与我的喜好类似，可选择面广，可以为用户提供了更加优质的使用体验和更多个性化家居选择。

Researcher

Why do you choose this brand of custom furniture?

您选择该品牌的定制家具的原因是什么？

Informant 024

I choose "Sophia" as the brand of customized furniture for the whole house, because "Sophia" has always adhered to the professional and dedicated expert spirit and frequently produced innovative designs to improve the living quality of users.

我选择的全屋定制家具的品牌是“索菲亚”，之所以选择他，是因为“索菲亚”一直秉持着专业、专注的专家精神，频频打出提升用户居住品质的创新设计。

Researcher

What do you think are the advantages of custom furniture over finished furniture?

您认为相比成品家具，定制家具的优势是什么?

Informant 024

Compared with finished furniture, customized furniture can be customized according to the user's special needs, for the user's house, for the user's ideas and needs, combined with the designer's experience and technology, a set of furniture suitable for the user. From these custom furniture, can reflect the style of the user.

相比成品家具，定制家具可以根据用户特殊需求，为用户的房子，为用户的想法和需求，结合设计师的经验和工艺，量身定做一套合适用户的家具。从这些定制家具上，可以反映出用户的风格。

Researcher

What do you think you should pay attention to when choosing custom furniture?

您觉得在选择定制家具时应该注意什么问题？

Informant 024

When choosing customized furniture, we should pay attention to the materials selected by customized furniture, whether it is safe and environmental protection, such as marble panel, whether it will release trace elements harmful to human body, whether the hole gap of furniture plate meets the national safety standards, whether there will be security risks.

在选择定制家具时应该注意定制家具所选择的材料，是否安全环保，例如大理石面板，是否会释放出对人体有害的微量元素，家具板材的孔间隙是否符合国家安全标准，是否会存在着安全隐患。

Researcher

How often do you use cabinets, closets, and other custom furniture?

您使用橱柜、衣柜、和其他定制的家具的频率是如何的？

Informant 024

I use it every day on average.

平均每天都会使用。

Researcher

Does the appearance of current custom furniture products meet your needs?

当前定制家具产品外观满足您的需求吗?

Informant 024

Basically satisfied, but I don't know whether I will get tired of looking at the color of this cabinet in the future.

基本可以满足，但不知道以后这个柜体的颜色我会不会看腻。

Researcher

Do current custom furniture products meet your needs with tactile details?
当前定制家具产品触觉细节满足您的需求吗?

Informant 024
Pretty much. It feels fine and smooth.
基本可以，摸上去挺细腻光滑的。

Researcher
Does the current custom furniture fit your functional needs? Which need is not being met?
当前的定制家具是否符合您对产品功能的需求？哪一个需求没有得到满足？

Informant 024
Pretty much. It would be better if custom furniture could be made smarter.
基本可以。如果定制家具可以再智能一些会更好。

Researcher
Does the current custom furniture meet your need for product audibility or smell?
当前定制家具是否符合您对产品可听性或气味的需求？

Informant 024
There are difficulties. At present, the audibility and smell of customized furniture products are not ideal. Perhaps some improvements can be made to give users a more comfortable experience.
存在困难。目前的定制家具产品的可听性和气味方面，都做的不够理想，或许可以进行一定改进，给予用户更加舒适的体验。

Researcher
How do you open and close your custom furniture? How do you like to open and close the door?

您家定制家具开关门方式是什么样的？您喜欢哪种开关门方式？

Informant 024

There are manual door opening and semi-automatic door opening. I prefer automatic door opening, which saves time and effort.

开关门方式主要有手动开门和半自动开门，我比较喜欢自动开门的方式，省时省力。

Researcher

Will you share your successful decorating experience with others?

您会与别人分享您的装修成功经验吗?

Informant 024

Yes, I am very happy to share my successful experience in decoration with others, so that I can provide good advice to my friends who are going to decorate. If there is a bad feeling in the decoration process, I can share it in time, so that my friends can avoid detours and lightning during decoration.

会，我非常乐意与别人分享我的装修成功经验，这样可以给即将进行装修的朋友提供良好的建议，如果有体验感不佳的装修过程，可以及时进行分享，朋友在进行装修时可以少走弯路，进行避雷。

Researcher

What do you think are the disadvantages of current custom furniture?

您觉得当前的定制家具的缺点是什么?

Informant 024

At present, the customization of customized furniture takes a long time. Users need to consume a lot of time from the communication with the designer, to the establishment of the scheme, to the contact of the factory for customization, and finally to the delivery to the customer. The conventional duration of a customized furniture project

is generally about 40 days. Private high quality requirements of the regular construction period: generally about 60 days; Specific because of the quantity, quality and complexity of the process. Traditional furniture users as long as they fancy, the day of the order, the next day can be shipped to the home, for installation.
当前的定制家具的定制耗时较长，用户从与设计师沟通，到方案的确立，到联系工厂进行定制，最后运输到客户手上，需要消耗大量的时间，定制家具工程单常规工期：一般 40 天左右；私宅高品质要求常规工期：一般 60 天左右；具体因数量、品质及工艺复杂程度。传统家具用户只要看中了，当天下单，第二天就可以运送到家里，进行安装。

Researcher
What other features do you think can be added to custom furniture?
您觉得定制家具可以添加什么其他功能？

Informant 024
The function of customized furniture can be started from the perspective of sustainability and growth. Take children's furniture in the children's bed for example, with the increase of children's age, the demand for children's bed is different, that is to say, children of different ages, if their parents choose customized children's bed, it may always need to be replaced, which will increase family expenses, and not environmental protection. Customized furniture can start from the growth of children, increase the function, design a growth type customized furniture suitable for children of all ages.
定制家具的功能方面，可以从可持续与成长性角度出发。以儿童家具中的儿童床为例，随着儿童年龄的增加，对于儿童床的需求也是不同的，也就是说不同年龄段的儿童，如果家长为其选择定制儿童床，可能会一直需要更换，这样既会增加家庭开支，又不环保。定制家具可以从儿童的成长性方面入手，对功能进行增加，设计出一款适合各个年龄段儿童的成长型定制家具。

Researcher

Okay, thank you for participating in this interview and have a great life.

好的，感谢您对本次访谈的参与，祝您生活愉快。
