# Supplementary material for: Building a 4E interview-grounded theory model: A case study of demand factors for customized furniture
Source: PLoS One. 2023 Apr 27;18(4):e0282956. doi: 10.1371/journal.pone.0282956 (PMC10138260; doi:10.1371/journal.pone.0282956)
Supplement: S1 File — (ZIP) [file pone.0282956.s001.zip › transcript/transcript 025.pdf]

Informant : 025

Please note that the original transcript is in Simplified Chinese. The English translation is for internal communication among the author of this research, and it is not proofread. Potential linguistic errors may exist in the English translation.

Thank you for your willingness to participate and be interviewed here. My name is XXX, and I'm a PhD in the XXX University. Currently, I am working on a research project that focuses on collecting information about user demand when purchasing and using customized furniture. Throughout the interview, I will ask you a series of questions and you are encouraged to express your opinions and views freely. During the interview, I will ask you if I have questions about what you have said or if I need you to clarify a topic or concept.

感谢您愿意参加并在此接受采访。我叫 XXX，是 XXX 大学的博士。目前，我正在开展一个研究项目，主要收集在使用定制家具时的用户体验资料。在整个访谈中，我会问您一系列问题，我们鼓励您自由表达您的意见和观点。在访谈过程中，如果我对你所说的内容有疑问或需要您澄清一个主题或概念，我会向您询问。

Researcher

Are you ready?

您准备好了吗？

Informant 025

Yes.

准备好了。

Researcher

First, some questions about yourself. How old are you now?

首先是关于您个人的一些问题。请问您现在的年龄是多少？

Informant 025

I am 23 years old.

我今年 23 岁。

Researcher

What kind of work are you doing now?

请问您现在从事什么工作呢？

Informant 025

I'm a student.

我是一名学生。

Researcher

What is the square footage of your house?

你的房子的面积是多少?

Informant 025

108 square meters.

108 平方米。

Researcher

How big is your family? What's the family structure like?

您的家庭人数？家庭结构是什么样的？

Informant 025

There are four people in my family, including my mother, brother, sister-in-law and myself.

家庭人数 4 人，母亲，哥哥，嫂子和我自己。

Researcher

What is the style of furniture in the home?

家中家具是什么样式的？

Informant 025

It's mainly Chinese furniture.

主要是中式家具。

Researcher

Where is the custom furniture placed? What are the main cabinets?

定制家具放置在哪里？主要是哪些柜体？

Informant 025

Where is the custom furniture placed? What are the main cabinets?

放在卧室，主要是嵌入式柜子。

Researcher

What is your custom furniture style? Is it consistent with the home decor?

您家定制家具风格是什么样？和家中装修风格一致吗？

Informant 025

Chinese furniture, consistent.

中式家具，一致。

Researcher
How much do you spend on custom furniture?
你花多少钱在定制家具上?

Informant 025

More than 10,000 yuan.

一万多元。

Researcher

What is your understanding of custom furniture?

您对定制家具的理解是什么？

Informant 025

Customized furniture is furniture enterprises on the basis of mass production, each consumer as a single customer, consumers according to their own requirements to design the furniture they want. Enterprises according to the design requirements of consumers, the manufacture of personal exclusive furniture.

定制家具是家具企业在大规模生产的基础上，将每一位消费者的视为单单独的客户，消费者根据自己的要求设计想要的家具。企业要根据消费者的设计要求，制造的个人专属家具。

Researcher
What do you know about custom furniture brand channels? (advertising or otherwise)
您了解定制家具品牌渠道是什么?（广告或其他）

Informant 025

Mainly through the public number advertising and wechat push to understand.

主要还是通过公众号广告和微信推送来了解。

Researcher
How do you know about custom furniture?
您是怎么了解定制家具相关内容?

Informant 025

Now with the development of technology, consumers can learn about customized furniture through a variety of channels. For example, you can search for relevant

information on the Internet, go to furniture fairs to listen to the introduction of furniture companies and so on.
现在科技发达，消费者可以通过多种渠道来了解定制家具相关内容。比如说，可以通过网上搜寻相关信息，到家具展览会现场听家具公司的介绍等等。

Researcher
What was your initial impression of the brand you chose? What was the initial understanding?
您对您选择的品牌最初印象是什么？最初的理解是什么？

Informant 025
My first choice of custom furniture brand left me with a simple, generous, simple, atmospheric, simple color impression. This style is particularly suitable for the decoration style of my home, and the pictures on the official website of the brand are also very beautiful.
我最初选择的品牌定制家具给我留下了简约，大方，简单，大气，颜色简约的印象。这种风格特别适合我家的装修风格，而且在品牌的官方网站看到的图片也非常精美。

Researcher
Why do you choose this brand of custom furniture?
您选择该品牌的定制家具的原因是什么？

Informant 025
The reason why I choose this brand of customized furniture is mainly because most of the furniture of this brand is simple and generous, simple and practical, and the color is also very eye-catching, which I like very much.
我之所以选择该品牌定制家具，主要是因为该品牌的家具大多数都是简约大方、简单实用，而且颜色也非常醒目，让我很喜欢。

Researcher
What do you think are the advantages of custom furniture over finished furniture?

您认为相比成品家具，定制家具的优势是什么?

Informant 025

The advantage of customized furniture compared with finished furniture is that you can customize the furniture you need according to your needs and hobbies, in line with your own mind. As a result, furniture styles, sizes and so on have more options to ensure that the final product meets the needs of the consumer.

定制家具相比成品家具的优势在于可以根据自己的需求和爱好来定制需要的家具，符合自己的心意。如此一来，家具的风格和尺寸等等都有了更多的选择，可以确保最终的产品满足消费者的需求。

Researcher

What do you think you should pay attention to when choosing custom furniture?

您觉得在选择定制家具时应该注意什么问题?

Informant 025

Customized furniture can make full use of the home space, and can be completely designed according to the actual situation of the owner, whether from the appearance or use can fully reflect the personalized needs, so, some such as wardrobe and other large furniture customization processing, should be a trend.

But the biggest risk of custom furniture is to buy can not fully foresee the finished product state, so when determining the design scheme must be carefully considered, the general business designers will give you a design drawing according to the size and your will, many people because they feel tired on the sloppy signature confirmation, to the later product installation only to find that there is something wrong.

定制家具可以充分的利用家中空间，并且可以完全的根据主人的实际情况来设计，无论从外观还是使用上都能充分体现个性化需求，所以，一些如衣柜等大件家具进行定制加工，应该是潮流趋势。

但定制家具最大的冒险就是购买时无法完全预见成品的状态，所以在确定设计方

案的时候一定要仔细斟酌，一般商家的设计师都会根据尺寸和你的意愿給你出设计图，很多人因为觉得看着累就草草的签字确认，到后来产品安装上才发现有不对的地方。

Researcher
How often do you use cabinets, closets, and other custom furniture?
您使用橱柜、衣柜、和其他定制的家具的频率是如何的？

Informant 025

I use it every day, about the same frequency.

每天都在使用，差不多一样的使用频率。

Researcher
Does the appearance of current custom furniture products meet your needs?
当前定制家具产品外观满足您的需求吗?

Informant 025

Yes, the basic look meets my needs.

满足，基本的外观就可以满足我的需求。

Researcher
Do current custom furniture products meet your needs with tactile details?
当前定制家具产品触觉细节满足您的需求吗?

Informant 025

Meet, the counter selected frosted texture material, very simple atmosphere.

满足，柜台选择了磨砂质感的材料，非常简约大气。

Researcher
Does the current custom furniture fit your functional needs? Which need is not being met?
当前的定制家具是否符合您对产品功能的需求？哪一个需求没有得到满足？

Informant 025

The current customized furniture is in line with my functional needs of the product, and I haven't found any shortcomings for the time being.

目前的定制家具符合我对产品功能的需求，暂时还没有发现任何不足之处。

Researcher

Does the current custom furniture meet your need for product audibility or smell?

当前定制家具是否符合您对产品可听性或气味的需求？

Informant 025

At present, the customized furniture in my home meets my demand for product audibility or smell. There is no odor or noise, and I am quite satisfied.

目前我家的定制家具符合我对产品可听性或气味的需求，没有任何异味和噪音存在，我还是比较满意的。

Researcher

How do you open and close your custom furniture? How do you like to open and close the door?

您家定制家具开关门方式是什么样的？您喜欢哪种开关门方式？

Informant 025

The custom furniture in my home opens and closes the door from the outside in. I especially like the shrinking opening and closing mode, which makes the space more tidy and beautiful.

我家的定制家具开关门方式是从外向里开门，我尤其喜欢收缩式的开关门方式，让空间显得更加整洁美观。

Researcher

Will you share your successful decorating experience with others?

您会与别人分享您的装修成功经验吗?

Informant 025

Yes, I often talk to my friends and family.

会的，经常和朋友和家人在聊天时说起。

Researcher

What do you think are the disadvantages of current custom furniture?

您觉得当前的定制家具的缺点是什么?

Informant 025

I think customized furniture is not equal to do what you want, sign the contract need to pay the full amount, custom furniture return difficult.

我认为定制家具不等于随心所欲，签合同需交全款，定制家具退货难。

Researcher

What other features do you think can be added to custom furniture?

您觉得定制家具可以添加什么其他功能？

Informant 025

Storage and lighting functions can be further optimized, and some functions can also be added according to the needs of the elderly and children. For the time being, I can think of adding the sterilization function of clothes for the elderly.

储物和照明功能还可以再进一步优化，也可以根据老人和儿童的需求增加一些功能，我暂时可以想到给老年人可以增加衣物杀菌功能。

Researcher

What aspects of custom furniture can provide more possibilities for users?

定制家具的哪些方面可以为用户提供更多的可能性?

Informant 025

Now the high sense of interior design often requires a unified home style, compared to spend time and energy to buy finished furniture everywhere, it is better to choose the unified customization of the whole house, to ensure that the style of furniture is always neat and unified, so that the interior space is more beautiful and harmonious.

The whole house custom home has its own planning style and main body, but still consider the overall planning, the whole home space environment is relatively coordinated.
现在高级感的室内设计往往要求统一家居风格，相比耗费时间和精力到处选购成品家具，不如选择全屋统一定制，确保家具的风格始终整齐统一，让室内空间更美观和谐。全屋定制家具有自己的规划风格和主体，但依然会考虑整体性的规划，整个居家空间环境是比较协调一致的。

Researcher

Okay, thank you for participating in this interview and have a great life.
好的，感谢您对本次访谈的参与，祝您生活愉快。
