# Supplementary material for: Building a 4E interview-grounded theory model: A case study of demand factors for customized furniture
Source: PLoS One. 2023 Apr 27;18(4):e0282956. doi: 10.1371/journal.pone.0282956 (PMC10138260; doi:10.1371/journal.pone.0282956)
Supplement: S1 File — (ZIP) [file pone.0282956.s001.zip › transcript/transcript 026.pdf]

Informant : 026

Please note that the original transcript is in Simplified Chinese. The English translation is for internal communication among the author of this research, and it is not proofread. Potential linguistic errors may exist in the English translation.

Thank you for your willingness to participate and be interviewed here. My name is XXX, and I'm a PhD in the XXX University. Currently, I am working on a research project that focuses on collecting information about user demand when purchasing and using customized furniture. Throughout the interview, I will ask you a series of questions and you are encouraged to express your opinions and views freely. During the interview, I will ask you if I have questions about what you have said or if I need you to clarify a topic or concept.

感谢您愿意参加并在此接受采访。我叫 XXX，是 XXX 大学的博士。目前，我正在开展一个研究项目，主要收集在使用定制家具时的用户体验资料。在整个访谈中，我会问您一系列问题，我们鼓励您自由表达您的意见和观点。在访谈过程中，如果我对你所说的内容有疑问或需要您澄清一个主题或概念，我会向您询问。

Researcher

Are you ready?

您准备好了吗？

Informant 026

Yes.

准备好了。

Researcher

First, some questions about yourself. How old are you now?

首先是关于您个人的一些问题。请问您现在的年龄是多少？

Informant 026

I am 25 years old.

我今年 25 岁。

Researcher

What kind of work are you doing now?

请问您现在从事什么工作呢？

Informant 026

I'm a vehicle engineer.

我是一名车辆工程师。

Researcher

What is the square footage of your house?

你的房子的面积是多少?

Informant 026

168 square meters.

168 平米。

Researcher

How big is your family? What's the family structure like?

您的家庭人数？家庭结构是什么样的？

Informant 026

Three people, with their children and husband.

三人、和孩子还有丈夫住在一起。

Researcher

What is the style of furniture in the home?

家中家具是什么样式的？

Informant 026

The furniture is mostly European style, mixed with a little Chinese furniture.

家具以欧式风格居多，混合少量中式家具。

Researcher

Where is the custom furniture placed? What are the main cabinets?

定制家具放置在哪里？主要是哪些柜体？

Informant 026

My custom furniture is mainly distributed in the bedroom, living room and kitchen three areas; They are large cabinet, wine cabinet, shoe cabinet and kitchen cabinet.

我家的定制家具主要分布于卧室、客厅厨房三个区域；分别是大立柜、酒柜、鞋柜和厨房柜。

Researcher

What is your custom furniture style? Is it consistent with the home decor?

您家定制家具风格是什么样？和家中装修风格一致吗？

Informant 026

Custom furniture is mostly European style, and home decoration style fit a higher degree.

定制家具大多是欧式风格、和家中装修风格契合度较高。

Researcher

How much do you spend on custom furniture?

你花多少钱在定制家具上?

Informant 026

Four custom-made models cost about 8,000 yuan.
定制四款共花了 8 千元左右。

Researcher
What is your understanding of custom furniture?
您对定制家具的理解是什么？

Informant 026
According to the area of each part of the family and the style of furniture decoration customization and design of different uses of furniture size and shape, at the same time can choose to meet the decoration budget of the board, hardware paint, to meet the owner's personalized needs.
根据家庭各部区域面积及家具摆设的风格定制和设计不通用途家具的尺寸与造型，同时可以选择符合装修预算的板材、五金件油漆，满足业主的个性化需求。

Researcher
What do you know about custom furniture brand channels? (advertising or otherwise)
您了解定制家具品牌渠道是什么?（广告或其他）

Informant 026
Through advertising and introductions from friends and family.
通过广告和亲友介绍。

Researcher
How do you know about custom furniture?
您是怎么了解定制家具相关内容?

Informant 026
Through the introduction materials of the store, the introduction of the web page

and the explanation of the shop assistant.

通过门店摆设的介绍资料、网页介绍和店员讲解了解。

Researcher

What was your initial impression of the brand you chose? What was the initial understanding?

您对您选择的品牌最初印象是什么？最初的理解是什么？

Informant 026

It is a large furniture customization brand, can provide professional furniture customization services for users.

他是一个较大型的家具定制品牌、可以专业地为用户提供家具的定制服务。

Researcher

Why do you choose this brand of custom furniture?

您选择该品牌的定制家具的原因是什么？

Informant 026

Brand benefit, cost performance and friends recommendation.

品牌效益、性价比和朋友的推荐。

Researcher

What do you think are the advantages of custom furniture over finished furniture?

您认为相比成品家具，定制家具的优势是什么?

Informant 026

Customized furniture can better fit the dimensions of each space in the house, so that the distance between the floor and the ceiling is appropriate and reasonable, at the same time can save most of the finished furniture is not the size of the empty area, to save the maximum amount of space;

Can design storage furniture according to user requirements of the block and internal modeling, more in line with the owner's storage and storage habits.
The price of customized furniture is lower than that of high-end finished furniture, and you can choose the quality of the board and the type of paint, which is convenient for each budget user group to customize to meet the psychological price of furniture.
Customized furniture can be changed according to the user's modeling needs, so that home decoration is more personalized.
The installation of customized furniture is convenient, can be assembled on site, in the process of transportation and assembly to avoid large areas of bump, save time and labor costs.
定制家具可以更加贴合房屋内各个空间的尺寸，让底板和天花板的距离恰当合理、同时能节约大部分因成品家具不合尺寸而空置的区域，最大程度地节约空间；
能根据用户的要求设计存储类家具的格挡和内部造型，更加贴合主人的存储和收纳习惯。
定制家具的价格相对于高端的成品家具较低，且可以选择板材的质量以及油漆种类，方便各个预算用户群体定制到符合心理价位的家具。
定制家具可以根据用户的造型需求改变，让家装更加个性化。
定制家具的安装便捷、可以现场拼装、在运输和装配的过程中避免了大面积磕碰、节约时间和人力成本。

Researcher
What do you think you should pay attention to when choosing custom furniture?
您觉得在选择定制家具时应该注意什么问题？

Informant 026
To ensure that the size measurement is accurate, before customization to a full range of reference to the field measurement room results and housing size map.
Select the appropriate furniture style of home decoration style, and consider the coordination of existing finished furniture; Make a list of your individual needs before

you communicate with the designer in case you miss anything.

Make layout location, quantity and budget assumptions before customization; Select the appropriate plate, hardware material.

After reviewing the design drawing, make suggestions for revision in time.

Before installation, we should communicate the time and place of the door.

要保证尺寸测量精准、定制前要全方位参考现场量房结果和房屋尺寸图。

挑选合适家装风格的家具样式，同时要考虑现有成品家具的配合；在与设计师沟通前列举好自己的个性化需求，以防遗漏。

定制前做好摆设位置、数量和预算设想；选择合适的板材、五金件的材料。

在看完设计图纸后及时提出修改意见。

安装前就要沟通好上门时间和地点。

Researcher

How often do you use cabinets, closets, and other custom furniture?

您使用橱柜、衣柜、和其他定制的家具的频率是如何的？

Informant 026

It's used every day.

每天都会使用。

Researcher

Does the appearance of current custom furniture products meet your needs?

当前定制家具产品外观满足您的需求吗?

Informant 026

I am quite satisfied.

还比较满意。

Researcher

Do current custom furniture products meet your needs with tactile details?

当前定制家具产品触觉细节满足您的需求吗?

Informant 026

Part of the waste material due to a long time will warping and wear, but does not affect the overall tactile effect.

部分边角料由于时间长了会起翘和磨损、但不影响整体触觉效果。

Researcher

Does the current custom furniture fit your functional needs? Which need is not being met?

当前的定制家具是否符合您对产品功能的需求？哪一个需求没有得到满足？

Informant 026

Better meet daily needs.

比较满足日常需求。

Researcher

Does the current custom furniture meet your need for product audibility or smell?

当前定制家具是否符合您对产品可听性或气味的需求？

Informant 026

Basically can meet.

基本可以满足。

Researcher

How do you open and close your custom furniture? How do you like to open and close the door?

您家定制家具开关门方式是什么样的？您喜欢哪种开关门方式？

Informant 026

Big cabinet is push-pull type cabinet door, wine cabinet, shoe cabinet and kitchen cabinet is switch cabinet door.

I prefer the switch type cabinet door, because it is easy to operate, each cabinet space will not affect each other when operating.

大立柜是推拉类柜门、酒柜、鞋柜和厨房柜是开关柜门。

我比较喜欢开关式柜门，因为操作简便，各个柜子空间在操作时不会相互影响。

Researcher

Will you share your successful decorating experience with others?

您会与别人分享您的装修成功经验吗?

Informant 026

Yes.

会。

Researcher

What do you think are the disadvantages of current custom furniture?

您觉得当前的定制家具的缺点是什么?

Informant 026

The production cycle is long and the production process is invisible.

The design process is complicated, and the time spent by the owner in understanding the material, size and communicating with the designer's shop assistants is too long, which is not suitable for the crowd with complicated work.

Materials, accessories, patterns and colors all need to be done by the owners themselves. The aesthetic degree has great fluctuation relative to the finished products, and the matching moderation after customization and addition can not be estimated in advance.

After customization, if the effect is not satisfied, it cannot be returned or replaced. It can only be customized again, which costs a considerable amount of time and

money.

制作的周期较长且生产过程不可见。

设计过程繁琐，业主花在了解材料、尺寸和与设计师店员沟通的时间过长，不适合工作繁琐的人群。

材料、配件、花纹、颜色都需要业主亲力亲为，美观度相对于成品家具有很大的波动性，且定制后与加装的配适度不能提前估量。

定制后如果效果不满意不能退换只能重新定制，耗费相当大时间金钱成本。

Researcher

What other features do you think can be added to custom furniture?

您觉得定制家具可以添加什么其他功能？

Informant 026

Switch automation, self cleaning, smart touch and voice.

开关自动化、自清洁、智能触摸和语音。

Researcher

What aspects of custom furniture can provide more possibilities for users?

定制家具的哪些方面可以为用户提供更多的可能性?

Informant 026

Combined with smart home, virtual reality can be used to simulate the effect of furniture placement in the house during customization. Use smart recommendation to modify unsuitable areas. Furniture customization can be involved in the ranks of smart home products.

结合智能家居，在定制时通过虚拟现实模拟家具在房屋内的摆放效果；通过智能推荐修改不适合的区域。可以让家具定制参与到智能家居产品的行列中。

Researcher

Okay, thank you for participating in this interview and have a great life.

好的，感谢您对本次访谈的参与，祝您生活愉快。
