# Supplementary material for: Building a 4E interview-grounded theory model: A case study of demand factors for customized furniture
Source: PLoS One. 2023 Apr 27;18(4):e0282956. doi: 10.1371/journal.pone.0282956 (PMC10138260; doi:10.1371/journal.pone.0282956)
Supplement: S1 File — (ZIP) [file pone.0282956.s001.zip › transcript/transcript 027.pdf]

Informant : 027

Please note that the original transcript is in Simplified Chinese. The English translation is for internal communication among the author of this research, and it is not proofread. Potential linguistic errors may exist in the English translation.

Thank you for your willingness to participate and be interviewed here. My name is XXX, and I'm a PhD in the XXX University. Currently, I am working on a research project that focuses on collecting information about user demand when purchasing and using customized furniture. Throughout the interview, I will ask you a series of questions and you are encouraged to express your opinions and views freely. During the interview, I will ask you if I have questions about what you have said or if I need you to clarify a topic or concept.

感谢您愿意参加并在此接受采访。我叫 XXX，是 XXX 大学的博士。目前，我正在开展一个研究项目，主要收集在使用定制家具时的用户体验资料。在整个访谈中，我会问您一系列问题，我们鼓励您自由表达您的意见和观点。在访谈过程中，如果我对你所说的内容有疑问或需要您澄清一个主题或概念，我会向您询问。

Researcher

Are you ready?

您准备好了吗？

Informant 027

Yes.

准备好了。

Researcher

First, some questions about yourself. How old are you now?

首先是关于您个人的一些问题。请问您现在的年龄是多少？

。

Informant 027

I am 29 years old.

我今年 29 岁。

Researcher

What kind of work are you doing now?

请问您现在从事什么工作呢？

Informant 027

I'm a freelancer.

我是一名自由职业者。

Researcher

What is the square footage of your house?

你的房子的面积是多少?

Informant 027

90 square meters.

90 平米。

Researcher

How big is your family? What's the family structure like?

您的家庭人数？家庭结构是什么样的？

Informant 027

Two people, me and my fiancee.

两人，我和我的未婚妻。

Researcher

What is the style of furniture in the home?

家中家具是什么样式的？

Informant 027

It's all reduced form.

都是简约式。

Researcher

Where is the custom furniture placed? What are the main cabinets?

定制家具放置在哪里？主要是哪些柜体？

Informant 027

In the living room, there are TV cabinets and display cabinets.

放在客厅，是电视柜和展示柜。

Researcher

What is your custom furniture style? Is it consistent with the home decor?

您家定制家具风格是什么样？和家中装修风格一致吗？

Informant 027

Simple, and home decoration style is consistent.

简约式，和家中装修风格一致。

Researcher

How much do you spend on custom furniture?

你花多少钱在定制家具上?

Informant 027

3000 to 5000 yuan.

3000-5000 元。

Researcher

What is your understanding of custom furniture?

您对定制家具的理解是什么？

Informant 027

Nowadays, the word "customized" has become a hot topic in the furniture market, and many consumers have experienced the advantages of customized furniture. So, for customized furniture, I think it can not only meet individual needs, integrate aesthetics and practicality, but also better reflect the space personality and unique style.

如今，“定制”一词成为了家具市场的热门话题，不少消费者体验了定制家具的优点。那么，对于定制家具，我认为它不仅能满足个性化需求，融汇美学和实用为一体，同时还能更好地体现出空间个性和独特风格。

Researcher

What do you know about custom furniture brand channels? (advertising or otherwise)

您了解定制家具品牌渠道是什么?（广告或其他）

Informant 027

In the custom furniture brand marketing channels, I think advertising and website search are the two most common ways. Many brands use the Internet and social media to keep in touch with consumers online and use advertising to increase brand awareness and influence.

在定制家具品牌的营销渠道中，我认为广告和网站搜索是两种最为常见的方式。许多品牌利用互联网和社交媒体，通过网络与消费者保持联系，并借助广告来提升品牌知名度和影响力。

Researcher

How do you know about custom furniture?

您是怎么了解定制家具相关内容?

Informant 027

I learn about customized furniture through short videos and website addresses, and judge the features and advantages of customized furniture brands by learning about some exclusive websites and platforms, browsing design cases, technological processes and customer comments.

我是通过短视频和网站网址来了解定制家具相关内容,通过了解一些专属网站和平台，浏览设计案例、工艺流程以及客户评价，来判断定制家具品牌的特点和优势。

Researcher

What was your initial impression of the brand you chose? What was the initial understanding?

您对您选择的品牌最初印象是什么？最初的理解是什么？

Informant 027

The initial impression of Shangpin House is that the company is well-known and easy to be trusted. The initial understanding of the brand is that it has rich experience and diverse styles.

对尚品宅配的最初印象是企业知名度比较高，易使人信任，对品牌的最初理解是经验丰富，风格多样。

Researcher

Why do you choose this brand of custom furniture?

您选择该品牌的定制家具的原因是什么？

Informant 027

The main reason why I choose customized furniture of this brand is that the

enterprise is well-known and diversified in style, which can meet my aesthetic needs. In addition, the brand team is professional and strong, which makes me more confident to choose this powerful brand.
我选择该品牌的定制家具的原因主要是因为企业知名度高，并且风格多样，能够满足我的审美需要。此外，品牌团队专业且实力雄厚，让我更加有信心选择这个拥有强大实力的品牌。

Researcher
What do you think are the advantages of custom furniture over finished furniture?
您认为相比成品家具，定制家具的优势是什么?

Informant 027
In contrast, custom furniture is more personalized, because the design of custom furniture can be tailored according to customer needs, to meet the personalized aesthetic and practical dual needs. And finished furniture is more common, from the design to the material, most furniture specifications and design are the same.
相比而言，定制家具更加个性化，因为定制家具的设计可以依据客户需求量身定制，满足个性化的美学与实用双重需求。而成品家具则显得较为普遍化，从设计到材料，多数家具的规格和设计都是一样的。

Researcher
What do you think you should pay attention to when choosing custom furniture?
您觉得在选择定制家具时应该注意什么问题?

Informant 027
In the selection of customized furniture, we should pay attention to the quality of materials, the designer's design concept and price factors, so as to avoid the omission of the design and the unreasonable choice of materials, leading to the dissatisfaction of customers.

在选择定制家具时，应该注意材料质量、设计师的设计理念以及价格因素等问题，以免出现设计上的疏漏和材料的不合理选择，导致客户心中的不满。

Researcher

How often do you use cabinets, closets, and other custom furniture?

您使用橱柜、衣柜、和其他定制的家具的频率是如何的？

Informant 027

Depending on my personal situation, I don't use cabinets, closets and other custom-made furniture very often, but these pieces of furniture better meet my specific needs and make my life more convenient.

根据个人情况，我使用橱柜、衣柜和其他定制的家具并不是特别频率，但是这些家具却能够更好地满足我的特殊需求，为我的生活提供了更多方便。

Researcher

Does the appearance of current custom furniture products meet your needs?

当前定制家具产品外观满足您的需求吗?

Informant 027

Current custom furniture products have met my needs in terms of appearance, design style and feel that satisfy my aesthetic concept.

当前的定制家具产品外观已经满足了我的需求，其设计风格和感觉都能够满足我的审美观念。

Researcher

Do current custom furniture products meet your needs with tactile details?

当前定制家具产品触觉细节满足您的需求吗?

Informant 027

It is OK. It feels warm and moist, but it is nearly so compared with solid wood

products.
还可以，摸起来手感比较温润，但和实木产品的比起来还是差点。

Researcher
Does the current custom furniture fit your functional needs? Which need is not being met?
当前的定制家具是否符合您对产品功能的需求？哪一个需求没有得到满足？

Informant 027
Basically, it is the feeling that the storage function of the existing customized furniture is relatively simple, and can not be adapted to their different functional storage environment.
基本上符合，就是感觉现有的定制家具的收纳功能比较单一，不能和自己不同功能收纳环境适配。

Researcher
Does the current custom furniture meet your need for product audibility or smell?
当前定制家具是否符合您对产品可听性或气味的需求？

Informant 027
General, generally satisfied, use does not feel particularly big taste and sound.
一般，大体上满足，使用起来没有感觉到特别大的味道和声音。

Researcher
How do you open and close your custom furniture? How do you like to open and close the door?
您家定制家具开关门方式是什么样的？您喜欢哪种开关门方式？

Informant 027
My custom home has single door, double door and sliding door type, I prefer the

double door mode.
我家的定制家具有单开门、双开门和推拉门式的，相比较而言我更喜欢双开门方式的。

Researcher
Will you share your successful decorating experience with others?
您会与别人分享您的装修成功经验吗?

Informant 027
Usually not. Everyone's home needs are different, but if someone asks, I'm happy to share.
一般不会，每个人家里所需要的都不太一样，但如果别人主动问我，我也是很乐意分享的。

Researcher
What do you think are the disadvantages of current custom furniture?
您觉得当前的定制家具的缺点是什么?

Informant 027
I think the customization time is relatively long, the design is complicated, and the price is relatively high.
我感觉是定制时间比较长，设计繁琐，价格也比较高。

Researcher
What other features do you think can be added to custom furniture?
您觉得定制家具可以添加什么其他功能？

Informant 027
I think the function of intelligent recommendation is quite useful. It can automatically recommend home design in line with my own aesthetic through

network intelligence, which saves a lot of time for me to talk with designers.

智能化推荐这一功能我感觉还挺有用的，可以通过网络智能自动为我推荐符合自身审美的家居设计，节省很多我与设计师的交谈时间。

Researcher

What aspects of custom furniture can provide more possibilities for users?

定制家具的哪些方面可以为用户提供更多的可能性?

Informant 027

For example, in the choice of materials, can increase the diversity; In space adaptation, some intelligent solutions can be provided. There are some more flexible ways to install to better meet the needs of users like me.

比如，在材料的选择上，可以增加多样性；在空间的适应上，可以提供一些智能化的方案；在安装方式上，可以提供一些更加灵活的方式，以更好地满足像我这样的使用者需求。

Researcher

Okay, thank you for participating in this interview and have a great life.

好的，感谢您对本次访谈的参与，祝您生活愉快。
