# Supplementary material for: Building a 4E interview-grounded theory model: A case study of demand factors for customized furniture
Source: PLoS One. 2023 Apr 27;18(4):e0282956. doi: 10.1371/journal.pone.0282956 (PMC10138260; doi:10.1371/journal.pone.0282956)
Supplement: S1 File — (ZIP) [file pone.0282956.s001.zip › transcript/transcript 028.pdf]

Informant : 028

Please note that the original transcript is in Simplified Chinese. The English translation is for internal communication among the author of this research, and it is not proofread. Potential linguistic errors may exist in the English translation.

Thank you for your willingness to participate and be interviewed here. My name is XXX, and I'm a PhD in the XXX University. Currently, I am working on a research project that focuses on collecting information about user demand when purchasing and using customized furniture. Throughout the interview, I will ask you a series of questions and you are encouraged to express your opinions and views freely. During the interview, I will ask you if I have questions about what you have said or if I need you to clarify a topic or concept.

感谢您愿意参加并在此接受采访。我叫 XXX，是 XXX 大学的博士。目前，我正在开展一个研究项目，主要收集在使用定制家具时的用户体验资料。在整个访谈中，我会问您一系列问题，我们鼓励您自由表达您的意见和观点。在访谈过程中，如果我对你所说的内容有疑问或需要您澄清一个主题或概念，我会向您询问。

Researcher

Are you ready?

您准备好了吗？

Informant 028

Yes.

准备好了。

Researcher

First, some questions about yourself. How old are you now?

首先是关于您个人的一些问题。请问您现在的年龄是多少？

Informant 028

I am 23 years old.

我今年 23 岁。

Researcher

What kind of work are you doing now?

请问您现在从事什么工作呢？

Informant 028

Still unemployed and preparing for the entrance exam.

还没有工作，正在准备升学考试。

Researcher

What is the square footage of your house?

你的房子的面积是多少?

Informant 028

105 square meters.

105 平方米。

Researcher

How big is your family? What's the family structure like?

您的家庭人数？家庭结构是什么样的？

Informant 028

Four, my parents, my sister and I.

4 口，父母我和妹妹。

Researcher

What is the style of furniture in the home?

家中家具是什么样式的？

Informant 028

Furniture at home is a very messy mix of styles. Different types and styles of furniture match each other, bringing a sense of randomness to the whole home space, which I like very much.

家中家具是一个非常杂乱无章的混合风格，不同种类和风格的家具相互搭配，给整个家居空间带来了一种随机的感觉，我非常喜欢。

Researcher

Where is the custom furniture placed? What are the main cabinets?

定制家具放置在哪里?主要是哪些柜体？

Informant 028

For custom furniture, we mainly consider placing in the bedroom, kitchen, wardrobe and cabinets and other furniture cabinets, because the space of these areas are relatively fixed, need to be tailored to create a more appropriate size and use effect.

对于定制家具，我们主要是考虑放置在卧室、厨房、衣柜和橱柜等家具柜体中，因为这些区域的空间都是相对固定的，需要通过量身定制的方式来打造出更加合适的尺寸和使用效果。

Researcher

What is your custom furniture style? Is it consistent with the home decor?

您家定制家具风格是什么样？和家中装修风格一致吗？

Informant 028

Our custom furniture style adopts a relatively simple style, which echoes the overall decoration style of the home, which can bring a more unified sense of the whole.

我们的定制家具风格采用了比较简约的风格，与家中整体的装修风格有所呼应，

可以带来一种更为统一的整体感。

Researcher

How much do you spend on custom furniture?

你花多少钱在定制家具上?

Informant 028

Basically the whole process down cost about 30,000.

基本整个流程走下来花费了三万左右。

Researcher

What is your understanding of custom furniture?

您对定制家具的理解是什么？

Informant 028

I think customized furniture is more tailored based on the needs of different families, in order to meet the special requirements of room specifications and home space layout, to create more perfect and suitable for living furniture.

我认为定制家具更多的是基于不同家庭的需求量身定制，以符合房间规格以及家居空间布局的特殊要求，打造出更加完美和适合居住的家具。

Researcher

What do you know about custom furniture brand channels?

您了解定制家具品牌渠道是什么?

Informant 028

We have learned that the brand channels of customized furniture are mainly through advertising and friends introduction, which can enable us to have a more comprehensive and in-depth understanding of the information and characteristics of different brands and products.

我们了解到定制家具的品牌渠道主要是通过广告和朋友介绍的方式，这些途径可以让我们更加全面和深入地了解到不同品牌以及产品的信息和特点。

Researcher

How do you know about custom furniture?

您是怎么了解定制家具相关内容?

Informant 028

By understanding some home custom furniture brand information, we can search online and other ways to deeply understand the relevant content, including brand characteristics, price, use effect and so on.

通过了解一些家居定制家具品牌的信息，我们可以通过网上搜索等方式去深度了解相关内容，包括品牌特点、价格、使用效果等方面。

Researcher

What was your initial impression of the brand you chose? What was the initial understanding?

您对您选择的品牌最初印象是什么?最初的理解是什么？

Informant 028

Our initial impression of the selected brand is very simple atmosphere, product design is very humanized and practical, can meet the needs of the majority of consumers.

我们最初对所选择的品牌的印象是非常简约大气，产品设计得十分人性化和实用，能够满足广大消费者的需求。

Researcher

Why do you choose this brand of custom furniture?

您选择该品牌的定制家具的原因是什么？

Informant 028

The main reason for choosing this brand is that its products are of high quality, beautiful and practical design, reasonable price, and after-sales service in place, which can meet our various needs and requirements.

最终选择该品牌的原因主要是因为它的产品质量很高，而且设计美观实用、价格合理、售后服务到位，可以满足我们的各种需求和要求。

Researcher

What do you think are the advantages of custom furniture over finished furniture?

您认为相比成品家具，定制家具的优势是什么?

Informant 028

There can be personalized customization, fully meet individual needs.

可以有个性化定制，完全符合个人需求。

Researcher

What do you think you should pay attention to when choosing custom furniture?

您觉得在选择定制家具时应该注意什么问题？

Informant 028

When choosing custom furniture, we should not only pay attention to the accuracy of specifications and dimensions, but also consider the compatibility with the overall space, the collocation of color style, the quality and reliability of material, so as to ensure the practicality and beauty of furniture.

在选择定制家具时，不仅要注意规格尺寸准确，还要考虑与整体空间的配合性、颜色风格的搭配、材质的质量和可靠性等问题，从而确保家具的实用性和美观性。

Researcher

How often do you use cabinets, closets, and other custom furniture?

您使用橱柜、衣柜、和其他定制的家具的频率是如何的？

Informant 028

I often use cabinets, closets, and other custom-made furniture because they help me better manage and utilize space and can meet my personal needs.

我经常使用橱柜、衣柜以及其他定制的家具，因为它们可以帮助我更好地管理和利用空间，并且能够满足我的个性化需求。

Researcher

Does the appearance of current custom furniture products meet your needs?

当前定制家具产品外观满足您的需求吗?

Informant 028

The appearance of the current customized furniture can meet my needs, because I can match different styles according to my own preferences and personality, so that the performance of the whole furniture is more diverse.

目前的定制家具外观可以满足我的需求，因为我可以根据自己的喜好和个性，搭配出不同的风格，使整个家具的表现更加丰富多样。

Researcher

Do current custom furniture products meet your needs with tactile details?

当前定制家具产品触觉细节满足您的需求吗?

Informant 028

The tactile details of the current customized furniture products also meet my needs, because I pay attention to the feel and texture of furniture, and like soft and comfortable materials and touch. The current customized furniture has done well in these points.

目前的定制家具产品触觉细节也比较满足我的需求，因为我注重家具的手感和质

感，喜欢柔和舒适的材质和触感，目前的定制家具这几点都做得不错。

Researcher

Does the current custom furniture fit your functional needs? Which need is not being met?

当前的定制家具是否符合您对产品功能的需求？哪一个需求没有得到满足？

Informant 028

The current customized furniture fully meets my demand for product functions, because I can customize furniture of different sizes and styles according to my own needs, which makes my home life more comfortable and convenient.

目前的定制家具完全符合我对产品功能的需求，因为我可以根据自己的需求定制出不同大小和风格的家具，让我的家居生活更加舒适和便捷。

Researcher

Does the current custom furniture meet your need for product audibility or smell?

当前定制家具是否符合您对产品可听性或气味的需求？

Informant 028

I don't have too many requirements for the audibility and smell of the product, as long as it doesn't affect the indoor air quality and comfort.

对于产品的可听性和气味需求，我没有太多的要求，只要不影响室内的空气质量和舒适感就可以。

Researcher

How do you open and close your custom furniture? How do you like to open and close the door?

您家定制家具开关门方式是什么样的？您喜欢哪种开关门方式？

Informant 028

Personally, I prefer the sliding mode of opening and closing doors, because it does not take up space and visually gives people a sense of overall harmony.
个人比较喜欢滑动形式的开关门方式，因为它不占用空间，视觉上给人一种整体和谐的感觉。

Researcher
Will you share your successful decorating experience with others?
您会与别人分享您的装修成功经验吗?

Informant 028
I would like to share my successful experience of decorating with others, because for me, it is an opportunity to learn and communicate, and can also help more people make their homes better.
我会非常乐意与别人分享我的装修成功经验，因为对我来说，这是一种学习和交流的机会，也可以帮助更多的人让自己的家居更加美好。

Researcher
What do you think are the disadvantages of current custom furniture?
您觉得当前的定制家具的缺点是什么?

Informant 028
At present, I have not found the disadvantages of the current custom furniture, and may need more detailed research and comparison to draw conclusions.
目前我还没有发现当前定制家具的缺点，可能需要更详细的研究和比较才能得出结论。

Researcher
What other features do you think can be added to custom furniture?
您觉得定制家具可以添加什么其他功能？

Informant 028

I think some intelligent elements can be added, such as intelligent induction and voice control, to make the furniture more intelligent and meet more needs.

我认为可以添加一些智能化的元素，比如智能感应、语音控制等，让家具更加智能化，并且可以满足更多的需求。

Researcher

What aspects of custom furniture can provide more possibilities for users?

定制家具的哪些方面可以为用户提供更多的可能性?

Informant 028

Or after sale, now buy things rather than buy things is to buy service, if the after-sales service is particularly good will attract a large number of people to buy.

还是售后吧，现在买东西与其说买东西不如说是买服务，如果售后服务特别好一定会吸引一大批复购人群。

Researcher

Okay, thank you for participating in this interview and have a great life.

好的，感谢您对本次访谈的参与，祝您生活愉快。
