# Supplementary material for: Building a 4E interview-grounded theory model: A case study of demand factors for customized furniture
Source: PLoS One. 2023 Apr 27;18(4):e0282956. doi: 10.1371/journal.pone.0282956 (PMC10138260; doi:10.1371/journal.pone.0282956)
Supplement: S1 File — (ZIP) [file pone.0282956.s001.zip › transcript/transcript 029.pdf]

Informant : 029

Please note that the original transcript is in Simplified Chinese. The English translation is for internal communication among the author of this research, and it is not proofread. Potential linguistic errors may exist in the English translation.

Thank you for your willingness to participate and be interviewed here. My name is XXX, and I'm a PhD in the XXX University. Currently, I am working on a research project that focuses on collecting information about user demand when purchasing and using customized furniture. Throughout the interview, I will ask you a series of questions and you are encouraged to express your opinions and views freely. During the interview, I will ask you if I have questions about what you have said or if I need you to clarify a topic or concept.

感谢您愿意参加并在此接受采访。我叫 XXX，是 XXX 大学的博士。目前，我正在开展一个研究项目，主要收集在使用定制家具时的用户体验资料。在整个访谈中，我会问您一系列问题，我们鼓励您自由表达您的意见和观点。在访谈过程中，如果我对你所说的内容有疑问或需要您澄清一个主题或概念，我会向您询问。

Researcher

Are you ready?

您准备好了吗？

Informant 029

Yes.

准备好了。

Researcher

First, some questions about yourself. How old are you now?

首先是关于您个人的一些问题。请问您现在的年龄是多少？

Informant 029

I am 25 years old.

我今年 25 岁。

Researcher

What kind of work are you doing now?

请问您现在从事什么工作呢？

Informant 029

Now I am an accountant in a bank.

现在是一家银行的会计师。

Researcher

What is the square footage of your house?

你的房子的面积是多少?

Informant 029

It's about 150 square meters.

约 150 平方米。

Researcher

How big is your family? What's the family structure like?

您的家庭人数？家庭结构是什么样的？

Informant 029

Four people, two kids and the father.

四个人，还有两个孩子和孩子爸爸。

Researcher

What is the style of furniture in the home?

家中家具是什么样式的？

Informant 029

Home furniture is mainly simple style, such a design style is more in line with the pursuit of modern families for cleanliness and comfort. The sofa in the living room, the dining room table and chairs, and the bed in the bedroom are designed in a simple style.

家中的家具是以简约风格为主，这样的设计风格更符合现代家庭对于整洁、舒适的追求。客厅的沙发、客餐厅的餐桌椅、以及卧室的床等等都是以简约风格来设计的。

Researcher

Where is the custom furniture placed? What are the main cabinets?

定制家具放置在哪里？主要是哪些柜体？

Informant 029

Custom furniture in the home is placed in some of the more important areas, including the room study, door, etc., the main cabinet including wardrobe, room shelves, bookcases and shoe cabinet, etc. This makes it easy to store and organize different items.

家中的定制家具都放置在一些比较重要的区域，包括房间内的书房、入户门边等等，主要的柜体包括衣柜、房间置物架、书柜以及鞋柜等等。这样方便储存和整理不同的物品。

Researcher

What is your custom furniture style? Is it consistent with the home decor?

您家定制家具风格是什么样？和家中装修风格一致吗？

Informant 029

Since we wanted to pursue the overall style of the home from the beginning, the

custom furniture also deliberately adopted a simple style, making the style of the entire home more consistent. Our custom furniture can be seen as an extension of the home style.
由于我们一开始就想要追求整体的家居风格，所以定制家具也特意采用简约风格，使得整个家居的风格更加一致。我们的定制家具可以看做是家装风格的延伸。

Researcher
How much do you spend on custom furniture?
你花多少钱在定制家具上?

Informant 029
We don't know exactly how much we spent on custom furniture, but we feel it was money well spent because of the space we got and the use we got.
关于花费在定制家具上的具体金额，我们已经不是很清楚了，感觉这些钱花得值得，因为我们所获得的空间和利用效果都很不错。

Researcher
What is your understanding of custom furniture?
您对定制家具的理解是什么？

Informant 029
Our understanding of customized furniture is: according to the specific needs of our family, in the appropriate place to consider the size, quality and other factors, and finally find furniture suitable for family style. In this way, the beauty and practicality of our home have been taken into account.
我们对于定制家具的理解是：根据我们家庭的具体需求，在合适的地方通盘考虑尺寸、品质等因素，最终找到适合家庭风格的家具。这样一来，我们家居的美观与实用性都得到了兼顾。

Researcher

What do you know about custom furniture brand channels? (advertising or otherwise)
您了解定制家具品牌渠道是什么?（广告或其他）

Informant 029
The brand channels we know are mainly recommended by relatives and friends, who have obtained satisfactory feelings from their own use of customized furniture, and hope that we can enjoy these benefits.
我们了解的品牌渠道主要是亲戚朋友推荐，他们从自己使用定制家具中获得了满意的感受，也希望我们能够享受到这些好处。

Researcher
How do you know about custom furniture?
您是怎么了解定制家具相关内容?

Informant 029
Through the development of the network, we have some basic concepts and knowledge of custom furniture have some understanding. We have learned some information about size, material, price and quality, which is of great help when choosing customized furniture.
通过网络的发展，我们对于定制家具的一些基本概念和知识已经有了一些了解。我们了解了一些尺寸、材质、价格以及质量等方面的信息，这些知识我们在选择定制家具的时候都有着很大的帮助。

Researcher
What was your initial impression of the brand you chose? What was the initial understanding?
您对您选择的品牌最初印象是什么？最初的理解是什么？

Informant 029

At first, the brand we choose has the impression of simplicity, practicality and high quality. When we see its advertisement, we feel that it is in line with our expectations.
最初对于我们选择的品牌有着简约、实用、高质的印象，看到它的广告时，就感觉它很符合我们的期望。

Researcher
Why do you choose this brand of custom furniture?
您选择该品牌的定制家具的原因是什么？

Informant 029
We choose this brand of custom furniture, mainly because it shows the advantages and services at a reasonable price, relatively more cost-effective. At the same time, it also provides a relatively comprehensive selection and customization scheme, which may be the decisive factor in our final choice.
我们选择这个品牌的定制家具，主要因为它将各项优点和服务以较合理的价格展现出来，相对来说更具有性价比。同时，它也提供了比较全面的选择和定制方案，可能是我们最终选择的决定性因素。

Researcher
What do you think are the advantages of custom furniture over finished furniture?
您认为相比成品家具，定制家具的优势是什么?

Informant 029
Compared with finished furniture, the advantage of customized furniture is that it can be customized according to the specific layout needs of the family, so it can make better use of space and achieve functional customization according to needs, such as layout porch, bedside table, window and other schemes. In addition, custom furniture can be designed according to individual aesthetic requirements, making the furniture better match the interior decoration style.

相较成品家具而言，定制家具的优势在于能够按照家庭的具体布局需求来定制家具，因此可以更好地利用空间，达到根据需要进行功能性定制，比如布置玄关、床头柜、窗户等方案。此外，定制家具可以按照个人的审美要求来设计，使家具更好地与室内装饰风格相匹配。

Researcher

What do you think you should pay attention to when choosing custom furniture?

您觉得在选择定制家具时应该注意什么问题？

Informant 029

When choosing custom furniture, we need to pay attention to the selection of high-quality raw materials, such as high-quality wood, plate, and so on. At the same time, we should pay attention to the practicality and durability of furniture, to see whether the process is fine and whether the goods are stable. In addition, the customization process needs to ensure the manufacturer's qualifications and reputation.

选择定制家具时，需要注意选用优质的原材料，比如优质的木材、板材等，同时要注重家具的实用性和耐久性，看看工艺是否精细，货品是否稳定。此外，定制过程需要确保产商的资质和信誉。

Researcher

How often do you use cabinets, closets, and other custom furniture?

您使用橱柜、衣柜、和其他定制的家具的频率是如何的？

Informant 029

Cabinets and wardrobes in my home are used very frequently, and the brand is a well-known brand, and the design style of the family is very coordinated. Shoe cabinet and bookcase are also used frequently, providing enough storage space and neatly placed, making life more comfortable and beautiful.

我家的橱柜和衣柜使用频率都很高，而且品牌是知名品牌，与家庭的设计风格十分协调。鞋柜和书柜的使用频率也很高，提供了足够的收纳空间，整齐摆放，使生活更加舒适美好。

Researcher
Does the appearance of current custom furniture products meet your needs?
当前定制家具产品外观满足您的需求吗?

Informant 029
The appearance of the current custom furniture products completely meets my needs. With independent design inspiration, based on the actual needs of families and decorative styles, to create products with personality and beauty.
目前定制家具产品的外观完全满足我的需求。拥有独立的设计灵感，以家庭的实际需求和装饰风格为基础，打造出具有个性和美感的产品。

Researcher
Do current custom furniture products meet your needs with tactile details?
当前定制家具产品触觉细节满足您的需求吗?

Informant 029
At present, the texture details and touch of customized furniture products fully meet my needs, such as the texture of the furniture surface and the gloss of the paint. The furniture cabinet handle feels comfortable, and it is light and smooth to use.
目前定制家具产品的质感细节和触感完全符合我的需求，比如家具表面的纹路和油漆的光泽，手握家具柜把感觉舒适，使用起来轻便流畅。

Researcher
Does the current custom furniture fit your functional needs? Which need is not being met?

当前的定制家具是否符合您对产品功能的需求？哪一个需求没有得到满足？

Informant 029

The current customized furniture fully meets my functional needs, such as the style, storage space and so on, and meets my expectations. In the furniture color and other aspects of the lack of a little, hope in this respect can provide more choices.

当前的定制家具完全满足我对产品功能的需求，比如款式，收纳空间等等方面都达到了我的期望。在家具色彩等方面略有欠缺，希望在这方面可以提供更多的选择。

Researcher

Does the current custom furniture meet your need for product audibility or smell?

当前定制家具是否符合您对产品可听性或气味的需求？

Informant 029

The current customized furniture fully meets my needs for product reliability and odor issues. My household customized furniture uses environmentally friendly raw materials, which will not harm indoor air quality.

目前的定制家具完全满足我对产品可靠性和气味问题的需求，我的家庭定制家具使用环保原材料，不会对室内空气质量造成伤害。

Researcher

How do you open and close your custom furniture? How do you like to open and close the door?

您家定制家具开关门方式是什么样的？您喜欢哪种开关门方式？

Informant 029

The custom furniture in our home has chosen different ways to open the door, such as the cabinets and bookcases are sliding door, while the wardrobe is moving door. I prefer push-pull doors because they don't take up space and are easier to use in

closets and shoe cabinets.
我们家的定制家具选择了不同的开门方式，如橱柜和书柜是推拉门的，而衣柜是移门的。我比较偏爱推拉式门，因为它不会占用空间，在使用衣柜和鞋柜时也更方便。
。
Researcher
Will you share your successful decorating experience with others?
您会与别人分享您的装修成功经验吗?

Informant 029
When someone asks me if I will share my decorating success, I am more than happy to share. In my opinion, sharing our experiences can help us gain more inspiration and knowledge so as to better plan and succeed in our renovation.
当有人问到我是否会分享我的装修成功经验时，我会非常乐意地分享。我认为，互相分享经验可以让我们吸取更多的灵感和知识，从而更好地规划装修计划和取得成功。

Researcher
What do you think are the disadvantages of current custom furniture?
您觉得当前的定制家具的缺点是什么?

Informant 029
One disadvantage of the current custom furniture is that some manufacturers use inferior panels in order to make more profit, which will affect the quality and time of the entire decoration. Therefore, when choosing the manufacturer, we should fully understand their products and production process to avoid unnecessary trouble and loss.
当前定制家具的一个缺点是有些厂家为了赚取更多的利润而使用劣质板材，这会影响整个装修的质量和时间。所以在选择厂家时要充分了解他们的产品和生产过

程，以避免不必要的麻烦和损失。

Researcher

What other features do you think can be added to custom furniture?

您觉得定制家具可以添加什么其他功能？

Informant 029

Customized furniture can add some intelligent functions, combined with smart home, such as smart home control system can be installed, control lighting, curtains and air conditioning, more convenient life for users.

定制家具可以添加一些智能化的功能，与智能家居相结合，例如可以安装智能家居控制系统，控制灯光、窗帘和空调等，更方便用户的生活。

Researcher

What aspects of custom furniture can provide more possibilities for users?

定制家具的哪些方面可以为用户提供更多的可能性?

Informant 029

The flexibility of custom furniture can provide users with more possibilities, enabling them to choose different shapes and styles according to their needs and preferences. For example, custom furniture can be selected according to the style and color of the home to create a unique home space.

定制家具的灵活性可以为用户提供更多的可能性，使他们可以根据自己的需求和喜好选择不同的造型和风格。例如，可以根据家居的格调和颜色选择合适的定制家具，以打造一个独具特色的家居空间。

Researcher

Okay, thank you for participating in this interview and have a great life.

好的，感谢您对本次访谈的参与，祝您生活愉快。
