# Supplementary material for: Building a 4E interview-grounded theory model: A case study of demand factors for customized furniture
Source: PLoS One. 2023 Apr 27;18(4):e0282956. doi: 10.1371/journal.pone.0282956 (PMC10138260; doi:10.1371/journal.pone.0282956)
Supplement: S1 File — (ZIP) [file pone.0282956.s001.zip › transcript/transcript 030.pdf]

Informant : 030

Please note that the original transcript is in Simplified Chinese. The English translation is for internal communication among the author of this research, and it is not proofread. Potential linguistic errors may exist in the English translation.

Thank you for your willingness to participate and be interviewed here. My name is XXX, and I'm a PhD in the XXX University. Currently, I am working on a research project that focuses on collecting information about user demand when purchasing and using customized furniture. Throughout the interview, I will ask you a series of questions and you are encouraged to express your opinions and views freely. During the interview, I will ask you if I have questions about what you have said or if I need you to clarify a topic or concept.

感谢您愿意参加并在此接受采访。我叫 XXX，是 XXX 大学的博士。目前，我正在开展一个研究项目，主要收集在使用定制家具时的用户体验资料。在整个访谈中，我会问您一系列问题，我们鼓励您自由表达您的意见和观点。在访谈过程中，如果我对你所说的内容有疑问或需要您澄清一个主题或概念，我会向您询问。

Researcher

Are you ready?

您准备好了吗？

Informant 030

Yes.

准备好了。

Researcher

How old are you now?

请问您现在的年龄是多少？

Informant 030

I am 31 years old.

我今年 25 岁。

Researcher

What kind of work are you doing now?

请问您现在从事什么工作呢？

Informant 030

I am now working as an engineer in a design institute.

我现在是在一家设计院做工程师。

Researcher

What is the square footage of your house?

你的房子的面积是多少?

Informant 030

The area of the house includes living room, dining area, kitchen and balcony, and the four bedrooms are about 100 square meters.

房子面积包含客厅、用餐区、厨房、阳台，4 间卧室大概有 100 平米。

Researcher

How big is your family? What's the family structure like?

您的家庭人数？家庭结构是什么样的？

Informant 030

The family size is six (parents and four children). The nuclear family consists mainly of parents and unmarried children.

家庭人数为六人（父母和 4 个小孩）核心家庭主要由父母和未婚子女组成。

Researcher

What is the style of furniture in the home?

家中家具是什么样式的？

Informant 030

There are mahogany, solid wood furniture. In the family furniture style mainly to American pastoral style furniture and old Chinese style. Mainly in accordance with the overall interior decoration style to buy furniture products.

有红木，实木家具为主。在家庭家具风格中主要以美式田园风格家具和旧中式风格为主。主要依照室内整体的装修风格选购家具产品。

Researcher

Where is the custom furniture placed? What are the main cabinets?

定制家具放置在哪里?主要是哪些柜体？

Informant 030

The kitchen and the study mainly take into account the high utilization rate of the two areas and the influence on the relationship between the living and eating habits of the householder. In this regard, parents will consider custom furniture. The main selection of particleboard medium fiber board moisture-proof board for the material of kitchen storage cabinets, top storage cabinets and vanity, bookshelf.

厨房和书房，主要考虑到两块区域利用率较高且对户主生活饮食习惯等关系影响。在这方面父母会考虑定制家具。主要选用刨花板中纤板防潮板为材料的厨房储物柜，顶部储物柜及洗手台，书架台。

Researcher

What is your custom furniture style? Is it consistent with the home decor?

您家定制家具风格是什么样？和家中装修风格一致吗？

Informant 030

Simple and generous style, combined with traditional Chinese style and pastoral style; Consistent.

简朴，大方的风格，复合传统中式和田园式风格；一致。

Researcher

How much do you spend on custom furniture?

你花多少钱在定制家具上?

Informant 030

10000 ~ 20000 yuan in custom furniture.

10000～20000 元在定制家具中。

Researcher

What is your understanding of custom furniture?

您对定制家具的理解是什么？

Informant 030

Tailored to meet the aesthetic needs of different users and other products. And through the way of customization design suitable for the interior furniture interior style space. For users to higher life experience and convenient living space.

量身定制，满足不同用户的生活审美需求等产品。并且通过定制的方式设计出适合户型内部的家具室内风格空间。为用户更高的生活体验和便利的生活空间。

Researcher

What do you know about custom furniture brand channels?

您了解定制家具品牌渠道是什么?

Informant 030

Online Zhihu XiaoHongshu bloggers recommend, friends, special furniture customization website to understand.

线上知乎小红书等博主推荐，朋友，专门的家具定制网站等进行了解。

Researcher

How do you know about custom furniture?

您是怎么了解定制家具相关内容?

Informant 030

The whole house is customized with some wardrobes, bookcases, wine cabinets, shoe cabinets and lockers, as well as many other different furniture products. Create independent, unified and personalized space for users to live and activity space. Meet the individual needs of different consumers.

全屋定制一些衣柜，书柜，酒柜，鞋柜储物柜为主的定制，还有更多其他不同的家具产品定制。为用户居住和活动空间创造出独立，统一，个性的空间。满足不同消费者的个性需求。

Researcher

What was your initial impression of the brand you chose? What was the initial understanding?

您对您选择的品牌最初印象是什么？最初的理解是什么？

Informant 030

To provide users with timely solutions and for the household owners concerned about the pain points to provide free personality and other services. Intimate, automatic solutions and budget, output modification suggestions one on one answer and service.

为用户提供及时的解决方案及为户主关心的痛点提供免费个性等服务建议。贴心，自动化提供解决方案及预算，产出修改建议一对一答疑及服务。

Researcher

Why do you choose this brand of custom furniture?

您选择该品牌的定制家具的原因是什么？

Informant 030

Reasonable budget, safe and reliable, around friends have customized this brand and the market reputation is good.

预算合理，安全可靠，身边朋友有定制过该品牌且市面上口碑较好。

Researcher

What do you think are the advantages of custom furniture over finished furniture?

您认为相比成品家具，定制家具的优势是什么?

Informant 030

Meet the personalized needs of users; Factory production workshop production to reduce the inconvenience caused by on-site production; Environmental protection and safety is easy to live a healthy body for users to create a more suitable space for users' living habits and aesthetic needs.

满足用户个性化需求；工厂生产车间生产减少现场制作带来的不便问题；环保安全有易于人生体健康健康为用户的活动空间打造更适合用户生活习惯及审美需求的空间。

Researcher

What do you think you should pay attention to when choosing custom furniture?

您觉得在选择定制家具时应该注意什么问题?

Informant 030

The advantages and disadvantages of price, material and furniture in practical use.

价格，材料以及家具实际运用中的利弊分析。

Researcher

How often do you use cabinets, closets, and other custom furniture?

您使用橱柜、衣柜、和其他定制的家具的频率是如何的？

Informant 030

Cabinets and wardrobes are used more frequently, mainly customized furniture in the kitchen is used more frequently, followed by seating areas, learning areas and tea drinking areas.

橱柜和衣柜较多使用，主要以厨房定制家具使用频率较高，其次便是餐座区域，学习区域，喝茶区域所涉及的座椅运用较多。

Researcher

Does the appearance of current custom furniture products meet your needs?

当前定制家具产品外观满足您的需求吗?

Informant 030

Barely can meet the coordination and unity of home decoration style.

勉强可以满足与家庭装修风格的协调统一。

Researcher

Do current custom furniture products meet your needs with tactile details?

当前定制家具产品触觉细节满足您的需求吗?

Informant 030

Barely satisfied, mainly in the hope of function and use comfort to meet more.

勉强满足，主要是希望功能和使用舒适上满足更多些。

Researcher

Does the current custom furniture fit your functional needs? Which need is not being met?

当前的定制家具是否符合您对产品功能的需求？哪一个需求没有得到满足？

Informant 030

The long service life of composite storage function is not satisfied.

复合储物功能使用寿命长的方面没有得到满足。

Researcher

Does the current custom furniture meet your need for product audibility or smell?

当前定制家具是否符合您对产品可听性或气味的需求？

Informant 030

No, there's some chemical smell in the cabinet.

不符合，柜体内有些化学药品的味道。

Researcher

How do you open and close your custom furniture? How do you like to open and close the door?

您家定制家具开关门方式是什么样的？您喜欢哪种开关门方式？

Informant 030

Flat door hidden, semi-hidden, flat door.

平开门隐藏式，半隐蔽式，平开门式。

Researcher

Will you share your successful decorating experience with others?

您会与别人分享您的装修成功经验吗?

Informant 030

Yes.

会。

Researcher

What do you think are the disadvantages of current custom furniture?

您觉得当前的定制家具的缺点是什么?

Informant 030

The appearance and function are more conventional, individuation degree is not high.

美观和功能上较为常规，个性化程度不高。

Researcher

What other features do you think can be added to custom furniture?

您觉得定制家具可以添加什么其他功能？

Informant 030

Hidden storage features and detail security considerations should be more comprehensive, and intelligent features can also be added.

隐藏收纳式功能以及细节安全方面的考虑应当更全面些，也可添加智能化功能。

Researcher

What aspects of custom furniture can provide more possibilities for users?

定制家具的哪些方面可以为用户提供更多的可能性?

Informant 030

Materials, comfort, cost-effective, unique private customization to provide more possibilities for users.

材料，舒适性，性价比高低，独特性私人定制打造性为用户提供更多可能性。

Researcher

Okay, thank you for participating in this interview and have a great life.

好的，感谢您对本次访谈的参与，祝您生活愉快。
