# Supplementary material for: Building a 4E interview-grounded theory model: A case study of demand factors for customized furniture
Source: PLoS One. 2023 Apr 27;18(4):e0282956. doi: 10.1371/journal.pone.0282956 (PMC10138260; doi:10.1371/journal.pone.0282956)
Supplement: S1 File — (ZIP) [file pone.0282956.s001.zip › transcript/transcript 031.pdf]

Informant : 031

Please note that the original transcript is in Simplified Chinese. The English translation is for internal communication among the author of this research, and it is not proofread. Potential linguistic errors may exist in the English translation.

Researcher

Thank you for your willingness to participate and be interviewed here. My name is XXX XXX, and I'm a PhD in the XXX University of XXX(XXX). Currently, I am working on a research project that focuses on collecting information about user demand when purchasing and using customized furniture. Throughout the interview, I will ask you a series of questions and you are encouraged to express your opinions and views freely. During the interview, I will ask you if I have questions about what you have said or if I need you to clarify a topic or concept.

感谢您愿意参加并在此接受采访。我叫 XXX，是 XXX 大学的博士。目前，我正在开展一个研究项目，主要收集在使用定制家具时的用户体验资料。在整个访谈中，我会问您一系列问题，我们鼓励您自由表达您的意见和观点。在访谈过程中，如果我对你所说的内容有疑问或需要您澄清一个主题或概念，我会向您询问。

Researcher

Are you ready?

您准备好了吗?

Informant 031

Yes.

准备好了。

Researcher

How old are you now?

首先是关于您个人的一些问题。请问您现在的年龄是多少？

Informant 031

I am 29 years old.

我今年 29 岁。

Researcher

What kind of work are you doing now?

请问您现在从事什么工作呢？

Informant 031

I am self-employed.

我是做个体经营的。

Researcher

What is the area of your house?

你的房子的面积是多少?

Informant 031

176 square meters

176 平方米

Researcher

How many people live in your house now?

您的房子现在又几个人在居住？

Informant 031

Two, me and my wife

两人，我和我的妻子

Researcher

What style of furniture is in the home?

家中家具是什么样式的？

Informant 031

Simple style, the whole is mainly white, the dining table is round, and the coffee table is square

简约风 整体以白色为主 餐桌为圆形，茶几为方形

Researcher

Where is the custom furniture placed? Which cabinets are the main ones?

定制家具放置在哪里？主要是哪些柜体？

Informant 031

The wardrobe is on one side of the bedroom, and the kitchen is a flat cabinet.

衣柜在卧室的一边，厨房里的是一字型的橱柜。

Researcher

What is your custom furniture style like? Is it consistent with the decoration style of the home?

您家定制家具风格是什么样？和家中装修风格一致吗？

Informant 031

They are all modern and simple, and the same style as home decoration.

都是现代简约风，和家里装修风格是一样的。

Researcher

How much do you spend on custom furniture?

你花多少钱在定制家具上?

Informant 031

I don't know, it was my parents who helped with the renovation.

不太清楚，是父母帮忙装修的。

Researcher

What is your understanding of custom furniture?

您对定制家具的理解是什么？

Informant 031

According to personal preference, space details, personalized furniture configuration, Furniture designed according to the size of the room type and the personal considerations, preferences, and financial resources of the residents.

根据个人喜好，空间细节，个性化的家具配置，按照本身房型的大小 以及住户的个人考虑、喜好、财力进行设计的家具。

Researcher

What do you know about the custom furniture brand channel?

您了解定制家具品牌渠道是什么?

Informant 031

Online inquiries and physical store knowledge

上网查询和实体店了解

Researcher

How did you learn about custom furniture?

您是怎么了解定制家具相关内容?

Informant 031

From the price, materials, other user feedback

从价格，用料，其他用户反馈

Researcher

What was your initial impression of the brand you chose? What was the initial understanding?

您对您选择的品牌最初印象是什么？最初的理解是什么？

Informant 031

Diversified styles, according to their own preferences customized unique furniture

款式多样化，可以根据自己的喜好定制独一无二的家具

Researcher

Why did you choose the brand's bespoke furniture?

您选择该品牌的定制家具的原因是什么？

Informant 031

Introduced by acquaintances, have a good reputation.

熟人介绍，口碑不错

Researcher

What do you think are the advantages of custom-made furniture over finished furniture?

您认为相比成品家具，定制家具的优势是什么?

Informant 031

The size of the furniture can be adjusted according to the details of the space, and the overall size layout can be adjusted in a regular and directional manner according to preferences. This design allows users to make better use of space and improve the quality of life. Through the combination of different materials, colors, layouts, etc., to create a harmonious living environment. For example, my wife prefers white, which can be matched with white cabinets or furniture, which also makes the arrangement of the home more orderly.

可以根据空间细节调整家具尺寸，根据喜好进行有规则、方向的调整整体的大小布局。这样的设计让用户可以更好的利用空间，提升生活品质。通过不同材质、颜色、布局等组合搭配，来营造一个和谐的居住环境。比如我妻子比较喜欢白色，就可以搭配白色的柜子或家具，这样的设计也让家居的布置更加有秩序感。

Researcher

What do you think you should pay attention to when choosing custom furniture?

您觉得在选择定制家具时应该注意什么问题？

Informant 031

Be sure to communicate the specific requirements of the furniture, such as the size, so as not to make it difficult to adjust later problems.

一定要沟通好家具的具体要求，比如尺寸这些，免得后期出现问题难以调节。

Researcher

How often do you use custom furniture?

您使用定制家具的频率是如何的？

Informant 031

High frequency.

较高频率。

Researcher

Does the current custom furniture product look meet your needs?

当前定制家具产品外观满足您的需求吗?

Informant 031

more satisfied

较为满足

Researcher

Does the current custom furniture fit your needs for product functionality?

当前的定制家具是否符合您对产品功能的需求？

Informant 031

In general, I think we can continue to achieve intelligent personalization, convenient for users to operate, and reduce the pressure of operation.

一般吧，我觉得还可以继续做到智能个性化，方便用户操作，减轻操作压力。

Researcher

What is the way your custom furniture opens and closes doors?

您家定制家具开关门方式是什么样的？

Informant 031

sliding door

推拉门

Researcher

Which way do you prefer to open and close doors?

您喜欢哪种开关门方式？

Informant 031

Electronic induction automatically opens the door

电子感应自动开门

Researcher

Will you share your renovation success with others?

您会与别人分享您的装修成功经验吗?

Informant 031

Yes

会

Researcher

What do you think are the disadvantages of current custom furniture?

您觉得当前的定制家具的缺点是什么?

Informant 031

One is that the price is not transparent. The material selection, interior design and style selection of furniture are all learning. If you don't understand, you may be trapped. The second is that the level of designers is uneven. The third is that the furniture production takes a long time, and the design and production time of customized furniture is too long. Our furniture has been waiting for more than half a year, so we must ask the merchants about the time it will take before purchasing.

一个是价格不透明，家具的材料选择、内部设计、风格选择，每一步都是学问，如果你不懂，很有可能被坑。二是设计师的水平参差不齐，三是家具制作时间久，定制家具的设计和制作时间太长，我们家的家具就等了半年多，所以购买前一定要提前询问一下商家所要花的时间。

Researcher

What other features do you think custom furniture can add?

您觉得定制家具可以添加什么其他功能？

Informant 031

Intelligent personalization

智能个性化

Researcher

Can you elaborate on your thoughts?

可以详细说一下您的想法吗？

Informant 031

For example, mold and dehumidification, disinfection and sterilization in the interior of the wardrobe shoe cabinet. Real-time monitoring, real-time monitoring of the amount of electricity and gas used in the kitchen. It can also remotely control the living environment in the user's home to improve the user's happiness in life.

比如除霉除湿，在衣柜鞋柜内部进行消毒杀菌。实时监控，实时监控厨房的用电、用气的量。还可以对用户家中的生活环境进行远程操控，提高用户生活的幸福感。

Researcher

Okay, thank you for this interview.

好的，谢谢您接受我们本次采访。
