# Supplementary material for: Building a 4E interview-grounded theory model: A case study of demand factors for customized furniture
Source: PLoS One. 2023 Apr 27;18(4):e0282956. doi: 10.1371/journal.pone.0282956 (PMC10138260; doi:10.1371/journal.pone.0282956)
Supplement: S1 File — (ZIP) [file pone.0282956.s001.zip › transcript/transcript 032.pdf]

Informant : 032

Please note that the original transcript is in Simplified Chinese. The English translation is for internal communication among the author of this research, and it is not proofread. Potential linguistic errors may exist in the English translation.

Researcher

Thank you for your willingness to participate and be interviewed here. My name is XXX , and I'm a PhD in the XXX University. Currently, I am working on a research project that focuses on collecting information about user demand when purchasing and using customized furniture. Throughout the interview, I will ask you a series of questions and you are encouraged to express your opinions and views freely. During the interview, I will ask you if I have questions about what you have said or if I need you to clarify a topic or concept.

感谢您愿意参加并在此接受采访。我叫 XXX，我是 XXX 大学的博士。目前，我正在开展一个研究项目，主要收集在使用定制家具时的用户体验资料。在整个访谈中，我会问您一系列问题，我们鼓励您自由表达您的意见和观点。在访谈过程中，如果我对你所说的内容有疑问或需要您澄清一个主题或概念，我会向您询问。

Researcher

Are you ready?

您准备好了吗?

Informant 032

Yes.

准备好了。

Researcher

First, some questions about yourself. How old are you now?

首先是关于您个人的一些问题。请问您现在的年龄是多少？

Informant 032

Informant 032

I am 20 years old.

我今年 20 岁。

Researcher

What kind of work are you doing now?

请问您现在从事什么工作呢？

Informant 032

I am a student.

我是一名学生。

Researcher

Who do you live with now?

您现在和谁一起居住？

Informant 032

Me and my parents.

我和父母。

Researcher

What is the area of your house?

您的房子的面积是多少?

Informant 032

103 ㎡.

103 ㎡。

Researcher

What style of furniture is in the home?

家中家具是什么样式的？

Informant 032

Minimalist style.

简约风格。

Researcher

Where is the custom furniture in your home placed?

您家的定制家具放置在哪里?

Informant 032

The room, kitchen and doorstep. Custom furniture are lockers, kitchen cabinets and shoe cabinets.

房间、厨房和家门口。柜子就是就是储物柜，橱柜和鞋柜。

Researcher

What is your custom furniture style like? Is it consistent with the decoration style of the home?

您家定制家具风格是什么样？和家中装修风格一致吗？

Informant 032

Yes, also minimalist style.

对，也是简约风格。

Researcher

What is your understanding of custom furniture?

您对定制家具的理解是什么？

Informant 032

Through the home company or factory, customize the size, color, style, etc. of the furniture according to the needs to meet the individual needs of panel cabinet furniture.

通过家居公司或工厂，根据需要定制家具的尺寸、颜色、风格等，满足个性化需求的板式柜体家具。

Researcher

What do you know about the custom furniture brand channel?

您了解定制家具品牌渠道是什么?

Informant 032

Advertisements, short videos, official account content. After buying a house and preparing to renovate, I will pay attention to these contents on my mobile phone at any time, so I will recommend content with similar themes later.

广告，短视频、公众号内容。买了房子，准备装修后，就会在手机上随时关注这些内容，所以后来都会推荐类似主题的内容。

Researcher

How did you learn about custom furniture?

您是怎么了解定制家具相关内容?

Informant 032

Online, I will go to the official website of relevant companies and Baidu Zhihu to learn about it, and offline, I will go to the home store to learn about it.线上的话，我会去相关公司的官网、百度知乎上了解，线下我会去家居店内了解。

Researcher

What was your initial impression of the brand you chose? What was the initial understanding?

您对您选择的品牌最初印象是什么？最初的理解是什么？

Informant 032

The style is simple, the board is more beautiful, textured, and looks good quality.

风格简约，板材比较美观，有质感，看上去质量不错。

Researcher

Why did you choose the brand's bespoke furniture?

您选择该品牌的定制家具的原因是什么？

Informant 032

Recommended by friends, big brand, feel good quality, moderate price, cost-effective, relatively healthy plate.

朋友推荐，大品牌，感觉质量不错，价格适中，性价比高，板材比较健康。

Researcher

What do you think are the advantages of custom-made furniture over finished furniture?

您认为相比成品家具，定制家具的优势是什么?

Informant 032

Compared with finished furniture, consumers can freely combine the products they need according to their own needs, which can not only meet individual needs, but also reduce costs. Moreover, it has a strong degree of flexibility and freedom, and its design can be personalized according to the consumer's preferences. It is customized according to the room type, which saves a lot of time and can maximize the use of space. You can customize the furniture according to your own likes and needs, regardless of the size and style of the furniture itself.

定制家具与成品家具相比，优消费者可以根据自身需要，通过自由组合自己需要

的产品，不仅能满足个性需求，还可以降低成本。而且它具有很强的灵活性和自由度，其设计上可以根据消费者的喜好进行个性化的改变。是根据房型来定制，省了很多时间，可以最大程度规划利用好空间。可以根据自己的喜欢和需求自定义家具，不受家具本身尺寸、风格的影响。

Researcher

What do you think you should pay attention to when choosing custom furniture?

您觉得在选择定制家具时应该注意什么问题？

Informant 032

First, whether the space arrangement is reasonable, whether the style matches and whether it is beautiful. To choose the right size of furniture according to the size of your home, you can see more and compare, choose the most suitable style, and match the decoration style of the home. Second, the structure is reasonable, choose the right plate hardware. Judging whether it is environmentally friendly from the thickness of the plate, formaldehyde emissions, etc., hardware accessories should be strictly checked. In addition, you must first communicate with the designer about the style and style you want before you decide on the furniture, do not blindly choose.

一是空间安排是否合理，风格匹配是否匹配，是否美观。要根据自己家的户型大小来选择合适尺寸的家具，可以多看多比较，选择最合适的款式，要跟家里的装修风格相搭配才可以。二是，结构合理，选择合适的板材五金件。从板材的厚度、甲醛释放量等方面判断是否环保，五金配件等要严格把关。另外还要先跟设计师沟通好自己想要的风格和样式之后再去确定家具，不要盲目的选择。

Researcher

How often do you use cabinets, wardrobes, and other custom furniture?

您使用橱柜、衣柜、和其他定制的家具的频率是如何的？

Informant 032

Wardrobe twice a day, cabinet three times a day， other no fixed use times

衣柜每日两次，橱柜每日三次，其他没有固定使用次数。

Researcher

Does the current custom furniture product look meet your needs?

当前定制家具产品外观满足您的需求吗?

Informant 032

It's OK

还可以

Researcher

Do the tactile details of current custom furniture products meet your needs?

当前定制家具产品触觉细节满足您的需求吗?

Informant 032

Nothing felt too much.

没有什么太大感觉。

Researcher

Does the current custom furniture fit your needs for product functionality? Which need is not being met?

当前的定制家具是否符合您对产品功能的需求？哪一个需求没有得到满足？

Informant 032

The feeling is general, and the cabinet storage is more cluttered. The cabinet storage area is not properly divided, making the whole kitchen look messy.

感受一般，橱柜收纳比较杂乱。橱柜收纳区域划分不合理，使得整个厨房显得凌乱不堪。

Researcher

What is the way your custom furniture opens and closes doors?

您家定制家具开关门方式是什么样的？

Informant 032

Wardrobe ——push and pull   cabinet ——flat open

衣柜——推拉   橱柜——平开

Researcher

Which way do you prefer to open and close doors?

您喜欢哪种开关门方式？

Informant 032

Flat open type

平开式

Researcher

Will you share your renovation success with others?

您会与别人分享您的装修成功经验吗?

Informant 032

Yes, if somebody asked.

会，如果有人询问的话

Researcher

What do you think are the disadvantages of current custom furniture?

您觉得当前的定制家具的缺点是什么?

Informant 032

High cost can not replace the work is difficult to ensure a long production cycle. The

storage space is too simple.
成本高，不可更换，做工难以保证，生产周期长。收纳空间太过单一。

Researcher

What other features do you think custom furniture can add?
您觉得定制家具可以添加什么其他功能？

Informant 032

Storage, decoration, storage space form can be changed. The change of space can be freely arranged according to the needs.
收纳，装饰，收纳空间形式可以变化。可以根据需求自由安排空间的变化。

Researcher

What aspects of custom furniture can provide users with more possibilities?
定制家具的哪些方面可以为用户提供更多的可能性?

Informant 032

Be smart. Realize the connection and interaction with other furniture, which can meet the needs of consumers at a certain time. There is also a storage space form that can be changed to meet the different forms of customer items placed in different forms, so that it looks more neat, not cluttered, and easy to take.
智能化吧。实现与其它家具之间的联系互动，可以满足消费者某一时间段多种需求。还有收纳空间形式可以变化，满足客户不同物品不同形态摆放空间，使其看上去更加整洁，不显凌乱，方便拿取。

Researcher

Okay, thank you for participating in this interview and have a great life.
好的，感谢您参与我们本次的访谈，祝您生活愉快。
