# Supplementary material for: Building a 4E interview-grounded theory model: A case study of demand factors for customized furniture
Source: PLoS One. 2023 Apr 27;18(4):e0282956. doi: 10.1371/journal.pone.0282956 (PMC10138260; doi:10.1371/journal.pone.0282956)
Supplement: S1 File — (ZIP) [file pone.0282956.s001.zip › transcript/transcript 033.pdf]

Informant : 033

Please note that the original transcript is in Simplified Chinese. The English translation is for internal communication among the author of this research, and it is not proofread. Potential linguistic errors may exist in the English translation.

Researcher

Thank you for your willingness to participate and be interviewed here. My name is XXX, and I’m a PhD in the XXX University. Currently, I am working on a research project that focuses on collecting information about user demand when purchasing and using customized furniture. Throughout the interview, I will ask you a series of questions and you are encouraged to express your opinions and views freely. During the interview, I will ask you if I have questions about what you have said or if I need you to clarify a topic or concept.

感谢您愿意参加并在此接受采访。我叫 XXX，是 XXX 大学的博士。目前，我正在开展一个研究项目，主要收集在使用定制家具时的用户体验资料。在整个访谈中，我会问您一系列问题，我们鼓励您自由表达您的意见和观点。在访谈过程中，如果我对你所说的内容有疑问或需要您澄清一个主题或概念，我会向您询问。

Researcher

Are you ready?

您准备好了吗?

Informant 033

Yes.

准备好了。

Researcher

First, some questions about yourself. How old are you now?

首先是关于您个人的一些问题。请问您现在的年龄是多少？

Informant 033

I am 42 years old.

我今年 42 岁。

Researcher

What kind of work are you doing now?

请问您现在从事什么工作呢？

Informant 033

A Company employee.

公司职员。

Researcher

What is the area of your house?

你的房子的面积是多少?

Informant 033

140 square meters

140 平米

Researcher

How many people live with your family now?

您家现在多少人一起居住？

Informant 033

5 people, me, my husband, daughter and my parents.

5 人，我、我丈夫、女儿还有我父母。

Researcher

What style of furniture is in the home?

家中家具是什么样式的？

Informant 033

Modern casual style, this style is all in the home.

现代休闲风，家里都是这种风格。

Researcher

Where is the custom furniture placed?

定制家具放置在哪里?

Informant 033

There is a wardrobe for clothes in the bedroom. In the kitchen is a storage cabinet for dishes and chopsticks

卧室内有放衣服的衣橱。厨房内是放置碗筷的收纳橱柜

Researcher

What is your understanding of custom furniture?

您对定制家具的理解是什么？

Informant 033

Customized furniture is "tailored" furniture according to the needs of users, and the rational use of space is carried out through design. It is well integrated with the indoor environment, and its form is simple and slightly decorated with patterns.

定制家具就是根据用户的需求进行“量身”打造的家具，通过设计进行空间的合理利用。与室内环境较好融合，其形式简洁，稍具纹样装饰点缀。

Researcher

What do you know about the custom furniture brand channel?

您了解定制家具品牌渠道是什么?

Informant 033

TV commercials, video sites

电视广告、视频网站

Researcher

How did you learn about custom furniture?

您是怎么了解定制家具相关内容?

Informant 033

Visit the Custom Furniture Factory website

访问定制家具工厂网站

Researcher

So will their official website image also influence your choice?

所以他们的官网形象也会影响您的选择吗？

Informant 033

Yes, I'll take a look at their excellent home cases to see if there's anything I can learn from.

是的，我会看看他们的优秀家居案例看看有没有什么可以借鉴的。

Researcher

What was your initial impression of the brand you chose?

您对您选择的品牌最初印象是什么?

Informant 033

The price is suitable for the people, the shape is simple, and there is no excessive decoration.

价格的适宜亲民，造型简洁，没有过分的装饰。

Researcher

So you prefer minimalist furniture?

所以您喜欢简约点的家具？

Informant 033

Yes, I like designs that are clean yet aesthetically pleasing. I think that too much decoration and cumbersome carvings often make the furniture look too gorgeous and complicated, but reduce the taste and style of the whole room.

是的，我喜欢那些造型简洁却不失美感的设计。我认为过多的装饰与繁琐的雕刻往往会使家具显得太过华丽和繁复，反而降低了整个房间的品味和格调。

Researcher

Why did you choose the brand's bespoke furniture?

您选择该品牌的定制家具的原因是什么？

Informant 033

Cheap, durable, simple and beautiful. Versatile and cost-effective furniture.

便宜，耐用，简约美观。百搭且性价比高的家具。

Researcher

您认为相比成品家具，定制家具的优势是什么?

Informant 033

Different from the fixed size and style of finished furniture, customized furniture can be freely combined according to the user's space utilization and preferences. This is something that finished furniture does not have. Customized furniture can be designed according to user preferences, and the overall home style is unified. In addition, the integrity of the customized furniture can be well integrated with the

interior space, giving people an overall comfortable feeling. High quality and low price, high quality and affordable, in the most economical situation to meet their own decoration requirements.

不同于成品家具固定的尺寸和样式，定制家具可根据用户对空间利用以及喜好等进行自由组合。这是成品家具所不具备的。定制家具可根据用户喜好进行设计，整体家居风格统一。此外定制家具的整体性能够与室内空间较好融合给人整体舒适的感觉。物美价廉、优质实惠，在最经济实惠情况下能够满足自身的装修要求。

Researcher

What do you think you should pay attention to when choosing custom furniture?

您觉得在选择定制家具时应该注意什么问题？

Informant 033

Price, decorative style, craftsmanship and durability. Style and durability are important to create a comfortable home environment. They can both provide the necessary functionality and serve as decorations in the home, creating an atmosphere unlike any other.

价格 装饰风格 工艺和耐用性。风格和耐久性对于打造一个舒适的家庭环境非常重要。它们既可以提供必要的功能性，又可以作为居室的装饰品，创造出一种与众不同的氛围。

Researcher

Craftsmanship is a thing that is difficult for ordinary consumers to understand, right?

工艺这个东西，普通消费者很难了解到吧？

Informant 033

Yes, so understand in advance, understand the plates and manufacturing processes, so as not to be deceived by the store.

是啊，所以提前了解，了解板材和制造工艺，免得被店家欺骗宰客。

Researcher

How often do you use custom furniture?

您使用定制家具的频率是如何的？

Informant 033

Kitchen cabinet are used every day, and wardrobes are used for changing seasons

橱柜天天用，衣柜换季用

Researcher

Does the current custom furniture product look meet your needs?

当前定制家具产品外观满足您的需求吗?

Informant 033

Okay, because we chose it.

尚可,因为是我们自己选择的呀。

Researcher

Do the tactile details of current custom furniture products meet your needs?

当前定制家具产品触觉细节满足您的需求吗?

Informant 033

To a certain extent, it is satisfied

一定程度上满足

Researcher

Does the current custom furniture fit your needs for product functionality?

当前的定制家具是否符合您对产品功能的需求？

Informant 033

Not very satisfied, no more features

不是很满意，没有更多的功能

Researcher

What other features do you think custom furniture can add?

您觉得定制家具可以添加什么其他功能？

Informant 033

Classify modules according to groups to store small items, such as clothes rails, clothes baskets and other small objects, modular design, according to your own needs to choose the required functional modules. In addition, the wardrobe can be folded and stored, and the clothes can be folded automatically, saving time and effort.

按组分类模块收纳小物件，如果挂衣杆、挂衣篮这些小物件，模块化设计，按照自己的需求选择需要的功能模块。另外，衣柜的话，可折叠收纳，自动折衣服，省时省力。

Researcher

What is the way your custom furniture opens and closes doors?

您家定制家具开关门方式是什么样的？

Informant 033

Hinged and mobile.

铰链式和移动式。

Researcher

Which way do you prefer to open and close doors?

您喜欢哪种开关门方式？

Informant 033

Push-pull type, relatively labor-saving.

推拉式，比较省力。

Researcher

Will you share your renovation success with others?

您会与别人分享您的装修成功经验吗?

Informant 033

Yes. I have shared my decoration experience in Xiaohong Book to remind others not to make the same mistakes as me.

会。我已经在小红书上分享了我的装修经验，提醒别人别和我踩一样的雷。

Researcher

What do you think are the disadvantages of current custom furniture?

您觉得当前的定制家具的缺点是什么?

Informant 033

First of all, the price, I think the current price of custom furniture is still inflated, and the price of furniture of big brands is not transparent. Secondly, many materials are not moisture-proof, easy to deform and difficult to clean, so the furniture cannot be used for a long time, and the durability is not enough.

首先是价格，我觉得目前定制家具的价格还是虚高的，大品牌的家具价格也不透明。其次，很多材料不防潮容易变形且难清洗，所以家具不能用很久，耐久性不够。

Researcher

What aspects of custom furniture can provide users with more possibilities?

定制家具的哪些方面可以为用户提供更多的可能性?

Informant 033

1. Users can design their own. For example, by importing floor plans in A PP, users

can freely choose the material, color, layout and so on of furniture. Customize furniture, such as customizing furniture according to ergonomics, and reasonably divide the space according to the size of the room to provide users with a more reasonable layout.

2. Barrier-free design, convenient for the elderly or children to operate the furniture. For example, the barrier-free design of the cabinets allows the elderly to complete cooking activities in the kitchen while ensuring basic safety.

1.可以让用户自己进行设计。比如在 APP 内导入户型图，用户可以自由选择家具的材质、颜色、布局等等。对家具进行定制，如根据人体工程学定制家具，根据房间面积大小，将空间进行合理划分，为用户提供更加合理的布局。

2.无障碍设计，方便老年人或者儿童对家具进行操作。比如橱柜的无障碍设计，让老年人在保证基本安全的状况下也可以在厨房完成烹饪活动。

Researcher

Okay, thank you for your valuable comments, and that's the end of our interview.

好的，感谢您提出的宝贵意见，我们的访谈就到此结束了。
