# Supplementary material for: Building a 4E interview-grounded theory model: A case study of demand factors for customized furniture
Source: PLoS One. 2023 Apr 27;18(4):e0282956. doi: 10.1371/journal.pone.0282956 (PMC10138260; doi:10.1371/journal.pone.0282956)
Supplement: S1 File — (ZIP) [file pone.0282956.s001.zip › transcript/transcript 034.pdf]

Informant : 031

Please note that the original transcript is in Simplified Chinese. The English translation is for internal communication among the author of this research, and it is not proofread. Potential linguistic errors may exist in the English translation.

Researcher

Thank you for your willingness to participate and be interviewed here. My name is XXX XXX, and I'm a PhD in the XXX University of XXX(XXX). Currently, I am working on a research project that focuses on collecting information about user demand when purchasing and using customized furniture. Throughout the interview, I will ask you a series of questions and you are encouraged to express your opinions and views freely. During the interview, I will ask you if I have questions about what you have said or if I need you to clarify a topic or concept.

感谢您愿意参加并在此接受采访。我叫 XXX，是 XXX 大学的博士。目前，我正在开展一个研究项目，主要收集在使用定制家具时的用户体验资料。在整个访谈中，我会问您一系列问题，我们鼓励您自由表达您的意见和观点。在访谈过程中，如果我对你所说的内容有疑问或需要您澄清一个主题或概念，我会向您询问。

Researcher

Are you ready?

您准备好了吗?

Informant 031

Yes.

准备好了。

Researcher

How old are you now?

首先是关于您个人的一些问题。请问您现在的年龄是多少？

Informant 031

I am 38 years old.

我今年 38 岁。

Researcher

What kind of work are you doing now?

请问您现在从事什么工作呢？

Informant 031

I am a van driver.

我是一名货车司机。

Researcher

What is the size of your house?

您的房子的面积为多少?

Informant 031

120 ㎡.

120 平方米。

Researcher

What is your current family like?

您目前的家庭是怎么样的？

Informant 031

6 people. We are husband and wife, two children, and my parents.

6 人。我们夫妻俩，两个孩子，还有我父母。

Researcher

What style of furniture is in the home?

家中家具是什么样式的？

Informant 031

Neoclassical

新古典式

Researcher

In which space is your home's custom furniture placed?

您家的定制家具放置在哪个空间里?

Informant 031

Kitchen, bedroom. Cabinets, wardrobes

厨房，卧室。橱柜、衣柜

Researcher

What is your custom furniture style like? Is it consistent with the decoration style of the home?

您家定制家具风格是什么样？和家中装修风格一致吗？

Informant 031

neoclassical style; unanimous

新古典式风格；一致

Researcher

How much do you spend on custom furniture?

你花多少钱在定制家具上?

Informant 031

Tens of thousands of yuan.

几万块。

Researcher

What is your understanding of custom furniture?

您对定制家具的理解是什么？

Informant 031

Based on the environment and size of the customer's home, combined with the customer's living habits, the merchant uses plates and hardware accessories, and uses relevant technology to customize personalized furniture that is extremely suitable for its home environment and people-oriented needs

商家以客户家里的环境和尺寸为基础，结合客户生活习惯，用板材和五金配件，运用相关工艺技术，定制出极度契合其家居环境与人本需求的个性化家具。

Researcher

What do you know about the custom furniture brand channel?

您了解定制家具品牌渠道是什么?

Informant 031

Ad. TV commercials, print ads, and more.

广告。电视广告、平面广告等等。

Researcher

How did you learn about custom furniture?

您是怎么了解定制家具相关内容?

Informant 031

Through online channels.

通过网上渠道。

Researcher

Can you elaborate?

可以详细说说吗？

Informant 031

It's almost a mobile phone video, and then the articles in APP will go to see.

差不多就是手机视频，然后 APP 内的文章都会去看一看。

Researcher

What was your initial impression of the brand you chose?

您对您选择的品牌最初印象是什么?

Informant 031

Elegant and meticulously crafted. Furniture specially tailored to the owner's home style and personal preferences.

典雅，精雕细琢。根据业主的家居风格和个人喜好进行特殊定制的家具。

Researcher

Why did you choose the brand's bespoke furniture?

您选择该品牌的定制家具的原因是什么？

Informant 031

Beautiful appearance, beautiful shape, cost-effective. It can be specially customized according to the owner's home style and personal preferences, and the colors and materials can be freely matched.

外形美观，造型优美，性价比高。可以根据业主的家居风格和个人喜好进行特殊定制，对色彩和材质进行随意搭配。

Researcher

What do you think are the advantages of custom-made furniture over finished

furniture?

您认为相比成品家具，定制家具的优势是什么?

Informant 031

1. Furniture that is easier to suit your own home situation and decoration style

2. Wood materials and sizes are available, and satisfaction is higher

1. 更加容易适合自己家情况和装修风格的家具

2. 木材材料和尺寸都可选，满意度更高

Researcher

What do you think you should pay attention to when choosing custom furniture?

您觉得在选择定制家具时应该注意什么问题？

Informant 031

Maximize the value of customized furniture according to the actual needs of your own family. The type and size of customized furniture products should be determined according to the area of the room.

根据自己家庭的实际需求，将定制家具的价值最大化。要根据居室面积确定定制家具产品的种类和大小。

Researcher

How often do you use cabinets, wardrobes, and other custom furniture?

您使用橱柜、衣柜、和其他定制的家具的频率是如何的？

Informant 031

The frequency of using cabinets and wardrobes is higher, and the rest is lower.

使用橱柜、衣柜频率较高，其它较低。

Researcher

Does the current custom furniture product look meet your needs?

当前定制家具产品外观满足您的需求吗?

Informant 031

Satisfy.

满足。

Researcher

Does the current custom furniture fit your needs for product functionality? Which need is not being met?

当前的定制家具是否符合您对产品功能的需求？哪一个需求没有得到满足？

Informant 031

Basic compliance. However, solid wood furniture is in a humid environment, which will cause the furniture to shrink, deform or crack.

基本符合。但实木家具处于潮湿环境，会造成家具收缩变形或开裂。

Researcher

What is the way your custom furniture opens and closes doors?

您家定制家具开关门方式是什么样的？

Informant 031

Swing doors, sliding doors.

平开门。移门。

Researcher

Will you share your renovation success with others?

您会与别人分享您的装修成功经验吗?

Informant 031

No. I will not actively share it with others.

不会。不会主动和别人分享。

Researcher

What do you think are the disadvantages of current custom furniture?

您觉得当前的定制家具的缺点是什么?

制作周期长，实际与想象落差大。定制家具需要一定的制作周期，部分因受特殊材质、工艺影响，无法定制或不建议定制。

Informant 031

The production cycle is long, and the gap between actual and imaginary is large. Custom furniture requires a certain production cycle, partly due to the influence of special materials and processes, can not be customized or is not recommended.

Researcher

What other features do you think custom furniture can add?

您觉得定制家具可以添加什么其他功能？

Informant 031

Personalized design according to the height and living habits of the occupants, and the layout of easy-to-use intelligent operation panels. Features such as wireless charging, environmental monitoring, and more can also be added. Through the environmental monitoring system, you can monitor the air quality, temperature, humidity and other environmental parameters in the cabinet, so as to make your home healthier and more comfortable. In addition, wireless charging can also be added to custom furniture. Wireless chargers can be installed on furniture such as bedside and desk, so that there is no need to use the charger cable, that is, there is no need to worry about the damage or loss of the charger cable. Custom furniture can also add some storage features. For example, installing a hidden storage space on a bookcase is not only aesthetically pleasing, but also improves storage efficiency.

根据居住者身高及生活习惯进行个性化设计，布局便于使用的智能操作面板。还可以添加无线充电，环境监测等功能。可以通过环境监测系统，监测柜内的空气质量、温度、湿度等环境参数，让您的家更加健康和舒适。此外，定制家具还可以添加无线充电功能。可以在床头、书桌上等家具上安装无线充电器，这样无需使用充电器线，也就是不需要担心充电器线的损坏或丢失的问题。定制家具也可以添加一些储藏功能。例如，在书柜上安装隐蔽的储藏空间，不仅美观，还能提高存储效率。

Researcher

What aspects of custom furniture can provide users with more possibilities?

定制家具的哪些方面可以为用户提供更多的可能性?

Informant 031

Customized furniture can provide users with more possibilities from: hardware, color matching, material, price, filling, maintenance, etc.

定制家具可以从：五金，配色，材质，价格，填充物，保养等方面为用户提供更多的可能性。

Researcher

Can you expand on that?

您可以展开说说吗？

Informant 031

Hardware refers to various accessories on furniture, such as handles, hinges, slide rails, etc. In the custom furniture, users can choose different materials and styles of hardware accessories according to personal preferences to meet their own needs. Color matching refers to the color matching of furniture, and furniture of different colors and materials can be selected according to home style and personal preferences, so as to create a unique personalized room. Material is the most important part of custom furniture, you can choose higher quality, more in line with

your own needs of materials, such as solid wood, panels, wood-based panels, etc. to make furniture. The price of custom furniture is also the most concerned issue of users, the price of customized furniture is relatively higher, but the degree of personalization is higher, which can meet the higher needs of users. Maintenance aspect is an important aspect of customized furniture, different materials and types of furniture need different maintenance methods, users can choose the appropriate maintenance method according to their own furniture to extend the service life of furniture.

五金方面指的是家具上的各种配件，如拉手、铰链、滑轨等。在定制家具中，用户可以根据个人喜好选择不同材质和风格的五金配件，以满足自己的需求。配色是指家具的颜色搭配，可以根据家居风格和个人喜好来选择不同颜色和材质的家具，从而打造出独具特色的个性化居室。材质方面则是定制家具中最重要的一个环节，可以选择更高品质，更符合自己需求的材质，如实木、板材、人造板等来制作家具。定制家具的价格也是一个用户最为关注的问题，定制家具的价格相对来说更高，但是个性化程度较高，可以满足用户更高的需求。保养方面是定制家具的一个重要方面，不同的材质和种类的家具需要不同的保养方式，用户可以根据自己的家具选择适合的保养方法，以延长家具使用寿命。

Researcher

Okay, thank you for accepting our interview and have a great life.

好的，谢谢您接受我们的采访，祝您生活愉快。
