# Supplementary material for: Building a 4E interview-grounded theory model: A case study of demand factors for customized furniture
Source: PLoS One. 2023 Apr 27;18(4):e0282956. doi: 10.1371/journal.pone.0282956 (PMC10138260; doi:10.1371/journal.pone.0282956)
Supplement: S1 File — (ZIP) [file pone.0282956.s001.zip › transcript/transcript 035.pdf]

Informant : 035

Please note that the original transcript is in Simplified Chinese. The English translation is for internal communication among the author of this research, and it is not proofread. Potential linguistic errors may exist in the English translation.

Researcher

Thank you for your willingness to participate and be interviewed here. My name is XXX, and I'm a PhD in the XXX University. Currently, I am working on a research project that focuses on collecting information about user demand when purchasing and using customized furniture. Throughout the interview, I will ask you a series of questions and you are encouraged to express your opinions and views freely. During the interview, I will ask you if I have questions about what you have said or if I need you to clarify a topic or concept.

感谢您愿意参加并在此接受采访。我叫 XXX，是 XXX 大学的博士。目前，我正在开展一个研究项目，主要收集在使用定制家具时的用户体验资料。在整个访谈中，我会问您一系列问题，我们鼓励您自由表达您的意见和观点。在访谈过程中，如果我对你所说的内容有疑问或需要您澄清一个主题或概念，我会向您询问。

Researcher

Are you ready?

您准备好了吗?

Informant 035

Yes.

准备好了。

Researcher

How old are you now?

首先是关于您个人的一些问题。请问您现在的年龄是多少?

Informant 035

I am 55 years old.

我今年 55 岁。

Researcher

What kind of work are you doing now?

请问您现在从事什么工作呢？

Informant 035

I am a housewife.

我是一名家庭妇女。

Researcher

What is the area of your house?

你的房子的面积是多少?

Informant 035

163 square meters.

163 平方米。

Researcher

How many people are in your household? What does the family structure look like?

您的家庭人数？家庭结构是什么样的？

Informant 035

The family size is 4 people and the family structure is a nuclear family. But now the two children are out of school and away from home.

家庭人数是 4 人，家庭结构是核心家庭，不过现在两个孩子外出上学了，不在家。

Researcher

What style of furniture is in the home?

家中家具是什么样式的？

Informant 035

The combination of Chinese and Western.

中西结合。

Researcher

Will the combination of Chinese and Western design at home be a bit obtrusive?

家中中西结合的设计会有点突兀吗？

Informant 035

No, the fusion is not bad, don't feel good.

不会，融合的还不错，别有一番感觉。

Researcher

Where is the custom furniture placed? Which cabinets are the main ones?

定制家具放置在哪里？主要是哪些柜体？

Informant 035

The study and the living room, the bookcase in the study and the coffee table in the living room are all custom furniture.

书房和客厅，书房的书柜和客厅的茶几都是定制家具。

Researcher

What is your custom furniture style like? Is it consistent with the decoration style of the home?

您家定制家具风格是什么样？和家中装修风格一致吗？

Informant 035

The combination of Chinese and Western, and home style similar.

中西结合，和家中风格相仿。

Researcher

How much do you spend on custom furniture?

你花多少钱在定制家具上?

Informant 035

About eighty thousand RMB.

八万左右。

Researcher

What is your understanding of custom furniture?

您对定制家具的理解是什么？

Informant 035

It is a furniture tailored to customers' needs and preferences, and is unique, belongs to itself and suits you.

是根据客户需求和喜好为客户量身定制的家具，是独一无二的、属于自己也适合自己的家具。

Researcher

What do you know about the custom furniture brand channel?

您了解定制家具品牌渠道是什么?

Informant 035

From shopping in furniture stores, seeing ads, reading tweets or watching videos.

逛家具城、看到广告、看推文或者刷视频了解到的。

Researcher

How did you learn about custom furniture?

您是怎么了解定制家具相关内容?

Informant 035

See similar furniture to consult information or to furniture city to consult.

看到类似的家具去查阅资料或者到家具城去咨询。

Researcher

What was your initial impression of the brand you chose? What was the initial understanding?

Informant 035

您对您选择的品牌最初印象是什么？最初的理解是什么？

Initial impression is very suitable for home style, also very beautiful, looks very practical and very suitable for themselves, the initial understanding is very in line with my preferences.

最初印象是很适合家中的风格，也很好看，看起来很实用也很适合自己，最初的理解就是十分符合我的喜好。

Researcher

So the brand's furniture that initially impressed you the most was the appearance, right?

所以该品牌的家具最初给您留下最深的印象就是外观是吗？

Informant 035

yes

是的

Researcher

Why did you choose the brand's bespoke furniture?

您选择该品牌的定制家具的原因是什么？

Informant 035

Because it's nice, it's in line with my tastes and preferences, and it looks very practical. The price was also within our expectations, so we chose it.

因为它好看，也符合我的品位和喜好，看起来也很实用。价格也在我们的预期之内，所以就选择了它。

Researcher

So the first impression of the brand is still very important, huh?

所以品牌的第一印象还是很重要的哈？

Informant 035

Yes.

是的。

Researcher

What do you think are the advantages of custom-made furniture over finished furniture?

您认为相比成品家具，定制家具的优势是什么?

Informant 035

The advantage of customized furniture is that it is unique. Customized furniture can be designed according to their own preferences and the space of the house. It is the furniture that they own only.

定制家具的优势是它的独一无二性，定制家具可以根据自己的喜好和房子的空间来设计，是只有自己拥有的家具，成品家具比较大众化，没有定制家具那么独特和适用。

Researcher

What do you think you should pay attention to when choosing custom furniture?

您觉得在选择定制家具时应该注意什么问题？

Informant 035

We should pay attention to the following problems: 1, whether it is suitable for the style of their own home; 2, to design the furniture suitable for their home space; 3, have their own characteristics.

应该注意的问题有：1，是否适合自己家中的风格；2，要设计合适自己家中空间的家具；3，拥有自己的特色。

Researcher

How often do you use the custom furniture?

您使用定制家具的频率是如何的？

Informant 035

Almost every day, three or four times a day.
几乎每天都会使用到，每天三四次。

Researcher

Do the tactile details of current custom furniture products meet your needs?

当前定制家具产品触觉细节满足您的需求吗?

Informant 035

Relatively satisfied.

比较满意。

Researcher

Does the current custom furniture fit your needs for product functionality? Which need is not being met?

当前的定制家具是否符合您对产品功能的需求？哪一个需求没有得到满足？

Informant 035

It is quite consistent, but most of the custom furniture in my home is relatively beautiful, if the unmet needs should be lack of more functionality.

还挺符合的，但我家的定制家具多数是比较好看的，如果说没有得到满足的需求应该是还缺乏更多的功能性。

Researcher

It sounds like you really care about the appearance of your furniture.

听起来，您是真的很在意家具的外观了。

Informant 035

Hahaha yes.

哈哈哈是的。

Researcher

What is the way your custom furniture opens and closes doors? Which way do you prefer to open and close doors?

您家定制家具开关门方式是什么样的？您喜欢哪种开关门方式？

Informant 035

Casement Windows, but I might prefer the sash Windows, because it saves space.

平开窗方式的，但我可能更喜欢推拉窗方式的，毕竟比较节约地方。

Researcher

Will you share your renovation success with others?

您会与别人分享您的装修成功经验吗?

Informant 035

Yes, I will be proud of the house I decorated successfully.

会的，我会为自己装修成功的房子而自豪。

Researcher

What do you think are the disadvantages of current custom furniture?

您觉得当前的定制家具的缺点是什么?

Informant 035

Either it can be completely customized, there are some design limitations, or there are some places can not be changed, for the pursuit of perfection people may need more choices.

不是可以完全自己定制的，有一些设计方面的限制，就是有一些地方是不能够更

改的，对于追求完美的人来说可能还需要更多选择。

Researcher

What other features do you think custom furniture can add?

您觉得定制家具可以添加什么其他功能？

Informant 035

It can be combined with AR and other technological means in advance to browse the effect of decoration.

可以结合 AR 之类的科技手段提前浏览装修好的效果。

Researcher

Well, this is a really good proposition so that we can imagine in advance what our house will look like after it is renovated.

唔，这确实是个很好的提议，这样我们就可以提前设想一下我们房子装修好后的样子了。

Informant 035

Yes, you can choose the right product for your room at this step.

是的，可以在这一步选择适合自己房间的产品。

Researcher

What aspects of custom furniture can provide users with more possibilities?

定制家具的哪些方面可以为用户提供更多的可能性?

Informant 035

Appearance and function, even according to user preferences to add some users like their own features.

外观和功能，甚至根据用户的喜好增添一些用户喜欢的自己的特色。

Researcher

For example, what are the features?

比如哪些特色呢？

Informant 035

I didn't think for a moment that the appearance was more personalized, not just the size customization.

我一时还想不到，就是外观更加的个性化吧，而不仅仅是尺寸的定制。

Researcher

Ok, thank you for accepting our interview.

好的，谢谢您接受我们的采访。
