# Supplementary material for: Building a 4E interview-grounded theory model: A case study of demand factors for customized furniture
Source: PLoS One. 2023 Apr 27;18(4):e0282956. doi: 10.1371/journal.pone.0282956 (PMC10138260; doi:10.1371/journal.pone.0282956)
Supplement: S1 File — (ZIP) [file pone.0282956.s001.zip › transcript/transcript 036.pdf]

Informant : 036

Please note that the original transcript is in Simplified Chinese. The English translation is for internal communication among the author of this research, and it is not proofread. Potential linguistic errors may exist in the English translation.

Researcher

Thank you for your willingness to participate and be interviewed here. My name is XXX, and I'm a PhD in the XXX University. Currently, I am working on a research project that focuses on collecting information about user demand when purchasing and using customized furniture. Throughout the interview, I will ask you a series of questions and you are encouraged to express your opinions and views freely. During the interview, I will ask you if I have questions about what you have said or if I need you to clarify a topic or concept.

感谢您愿意参加并在此接受采访。我叫 XXX，是 XXX 大学的博士。目前，我正在开展一个研究项目，主要收集在使用定制家具时的用户体验资料。在整个访谈中，我会问您一系列问题，我们鼓励您自由表达您的意见和观点。在访谈过程中，如果我对你所说的内容有疑问或需要您澄清一个主题或概念，我会向您询问。

Researcher

Are you ready?

您准备好了吗?

Informant 036

Yes.

准备好了。

Researcher

How old are you now?

请问您现在的年龄是多少?

Informant 036

I am 46 years old.

我今年 46 岁。

Researcher

What kind of work are you doing now?

请问您现在从事什么工作呢？

Informant 036

I am a construction engineer.

我是一名建筑工程师。

Researcher

What is the area of your house?

你的房子的面积是多少?

Informant 036

140 square meters

140 平方

Researcher

How many people are in your household? What does the family structure look like?

您的家庭人数？家庭结构是什么样的？

Informant 036

There are 3 people in our family and we are a nuclear family

我们家有 3 人，是核心家庭

Researcher

What style of furniture is in the home?

家中家具是什么样式的？

Informant 036

Modern simple furniture

现代简约家具

Researcher

Where is the custom furniture placed? Which cabinets are the main ones?

定制家具放置在哪里？主要是哪些柜体？

Informant 036

Our custom furniture is in the living room and kitchen, the living room has a solid wood cabinet, and the kitchen is an ordinary cabinet.

我们家的定制家具在客厅和厨房，客厅有个实木柜子，厨房就是普通的橱柜。

Researcher

What is your custom furniture style like? Is it consistent with the decoration style of the home?

您家定制家具风格是什么样？和家中装修风格一致吗？

Informant 036

All are modern minimalist style, the style is unified.

都是现代简约风格，风格是统一的。

Researcher

What is your understanding of custom furniture?

您对定制家具的理解是什么？

Informant 036

It can meet the individual needs of consumers for furniture and meet personal preferences. Make the most of the space in the house to maximize the storage area.
可以满足消费者对家具的个性需求，满足个人喜好。充分利用房屋空间，最大程度的增大收纳面积。

Researcher

What do you know about the custom furniture brand channel?

您了解定制家具品牌渠道是什么?

Informant 036

Advertising, offline physical stores, Internet search

广告、线下实体店、互联网搜索

Researcher

How did you learn about custom furniture?

您是怎么了解定制家具相关内容?

Informant 036

Baidu

百度

Researcher

What was your initial impression of the brand you chose? What was the initial understanding?

您对您选择的品牌最初印象是什么？最初的理解是什么？

Informant 036

The brand we chose is Sophia. The initial understanding is that the furniture configuration can be personalized according to personal preferences, space details.

我们选择的品牌是索菲亚。最初对它的理解就是可以根据个人喜好、空间细节，个性化的家具配置。

Researcher

Why did you choose the brand's bespoke furniture?

您选择该品牌的定制家具的原因是什么？

Informant 036

Rich experience in customized furniture and beautiful design products. Big brands are trustworthy.

对于定制家具相关经验丰富、设计产品较为美观。大品牌值得信赖。

Researcher

What do you think are the advantages of custom-made furniture over finished furniture?

您认为相比成品家具，定制家具的优势是什么?

Informant 036

Meet everyone's personalized needs

满足每个人个性化需求

Researcher

What do you think you should pay attention to when choosing custom furniture?

您觉得在选择定制家具时应该注意什么问题？

Informant 036

Quality and the realization of and customer needs

质量和客户需求的实现问题

Researcher

How often do you use cabinets, wardrobes, and other custom furniture?

您使用橱柜、衣柜、和其他定制的家具的频率是如何的？

Informant 036

High frequency, often used in daily life

频率高，日常经常使用

Researcher

Does the current custom furniture product look meet your needs?

当前定制家具产品外观满足您的需求吗?

Informant 036

The appearance was chosen by ourselves, so we are satisfied.

外观是我们当初自己挑选的，所以我们很满意。

Researcher

Do the tactile details of current custom furniture products meet your needs?

当前定制家具产品触觉细节满足您的需求吗?

Informant 036

You can continue to optimize

还可以继续优化

Researcher

Can you elaborate?

您可以详细点说吗？

Informant 036

It is to make the touch of the product better, such as making ordinary boards into the texture and haptics of solid wood.

就是把产品的触感做的更好一点，例如把普通板材做出实木的纹理与触感。

Researcher

Does the current custom furniture fit your needs for product audibility or smell?

当前定制家具是否符合您对产品可听性或气味的需求？

Informant 036

At the beginning, the furniture still had some smell, I don't know if it was the smell of glue or the smell of the board, and then it took half a year to open the window to breathe better. But after a long time of furniture use, some hardware will rust, and pulling it will make a sharp sound.

刚开始的时候，家具还是有些味道的，不知道是胶水的味道还是板材散发的味道，后来开窗透气了半年才好点。但是家具用久了，有些五金就会上锈了，拉动它就会发出尖锐的声音。

Researcher

What is the way your custom furniture opens and closes doors? Which way do you prefer to open and close doors?

您家定制家具开关门方式是什么样的？

Informant 036

The furniture doors of our home are mainly sliding doors, ordinary double doors and pull-out drawers.

我们家的家具的门是主要就是推拉门、普通双开门和抽拉式的抽屉。

Researcher

Which way do you prefer to open and close doors?

您喜欢哪种开关门方式？

Informant 036

I think different spaces can use different ways to open and close doors, if the space is small, try to choose sliding doors, do not occupy space. Drawers can be selected on the upper floor of the floor cabinet in the kitchen.
我觉得不同的空间可以采用不同的开关门方式，空间小的话，就尽量选择推拉门，不占空间。厨房内的地柜上层就可以选择抽屉。

Researcher

Will you share your renovation success with others?

您会与别人分享您的装修成功经验吗?

Informant 036

yes

会

Researcher

What do you think are the disadvantages of current custom furniture?

您觉得当前的定制家具的缺点是什么?

Informant 036

Not smart enough

不够智能

Researcher

What other features do you think custom furniture can add?

您觉得定制家具可以添加什么其他功能？

Informant 036

Cabinet drawers can be set up as tactile or push-to-switch switches so that hands covered with oil or water can open the door without touching the handle. Intelligent service

橱柜的抽屉可以设置为触碰式或者按压式开关，这样沾满油或水的手就可以不碰到把手就可以把门打开了。智能化服务

Researcher

What aspects of custom furniture can provide users with more possibilities?

定制家具的哪些方面可以为用户提供更多的可能性?

Informant 036

Intelligent personality

个性化智能化

Researcher

Can you elaborate on the intelligence you need?

您可以详细说一下您所需要的智能化吗？

Informant 036

For example, the automatic sanitize function of wardrobes and shoe cabinets. There is also a smart recommendation function of the wardrobe, which automatically matches and recommends clothes according to the daily temperature. These smart home applications make our lives more convenient and comfortable.

比如衣柜和鞋柜的自动消杀功能。还有衣柜的智能推荐功能，根据每天的温度，对衣物进行自动搭配与推荐。这些智能家居的应用汇让我们的生活变得更加方便与舒适。

Researcher

Uh-huh, got it, then we still have a long way to go.

嗯嗯，了解了，那我们还有很长的一段距离要走啊。

Y

Informant 036

es, I hope that these intelligences can be popularized as soon as possible, so that

ordinary people can also experience these functions as soon as possible.
是啊，希望这些智能化能够早点实现普及，让我们这些普通人也可以早日体验这些功能。

Researcher

Okay, thank you for receiving our interview.
好的，谢谢您接收我们的访谈。
